# Supplementary material for: High‐Throughput Synthesis and Screening of a Cyanimide Library Identifies Selective Inhibitors of ISG15‐Specific Protease mUSP18
Source: Angew Chem Int Ed Engl. 2025 Oct 7;64(49):e202510941. doi: 10.1002/anie.202510941 (PMC12668310; doi:10.1002/anie.202510941)
Supplement: Supplementary file 1 — Supporting Information [file ANIE-64-e202510941-s003.pdf]

SUPPORTING INFORMATION

High-Throughput Synthesis and Screening of a Cyanimide Library Identifies Selective Inhibitors of ISG15-specific Protease mUSP18

Raymond Kooij,[a]+ Vito Pol,[a]+ Jin Gan,[a]+ Bjorn R. van Doodewaerd,[a] Aysegul Sapmaz,[a] Marta Campos Alonso,[b] Günter Fritz,[c] Klaus-Peter Knobeloch,[b,d] and Paul P. Geurink[a]*

[a] Department of Cell and Chemical Biology, Division of Chemical Biology and Drug Discovery, Leiden University Medical Center, Einthovenweg 20, 2333 ZC, Leiden, The Netherlands.

[b] Institute of Neuropathology, Faculty of Medicine, University of Freiburg, 79106 Freiburg, Germany.

[c] Department of Cellular Microbiology, University of Hohenheim, 70599 Stuttgart, Germany.

[d] Signalling Research Centres BIOSS and CIBSS, University of Freiburg, 79106 Freiburg, Germany

[+] Equal contribution

* Corresponding author: p.p.geurink@lumc.nl

Contents

Figure S1. IC_{50} determination of compounds **1-5**…………………………………………………..S3

Figure S2. Full spectrum LC-MS analysis of the model reaction shown……………………...S4

Figure S3. LCMS analysis of the first generation compound library………………………..…S5

Figure S4. Plate outline of all 1,536 well synthesis plates…………………………………...S6

Figure S5. LCMS analysis of plate NCN-4……………………………………………………….S7

Figure S6. LCMS analysis of plate NCN-5……………………………………………………….S8

Figure S7. IC_{50} determination of hit compounds for USP30, papain, and hUSP18..…...…...S9

Figure S8. Determination of k_{inact} and K_i values for hit compounds on mUSP18…………..S10

Figure S9. LC-MS analysis of USP18-BB07CA902 covalent complex……………………...S11

Figure S10. Gel-based ABPP selectivity assessment using Rho-Ub-PA…………………...S12

Figure S11. Full image scan of Rho-mISG15ct-PA probe labelled enzymes……………….S13

Figure S12. ISGylation assay in IFN-β-stimulated SUP-T1 cells…………………….………S14

Figure S13. NanoBRET assays to assess mUSP18 interaction with ISG15 and STAT2…S15

Figure S14. NanoBRET assays to assess p53/MDM2 interaction and cell viability assay..S16

Figure S15. NanoBRET assays to assess hUSP18 interaction with ISG15 and STAT2….S17

Synthesis………………………………………………………………………………………...S18

Echo synthesis and analysis…………………………………………………………………..S31

Biochemistry methods…………………………………………………………………………S35

References……………………………………………………………………………………...S43

NMR and LC-MS spectra……………………………………………………………………..S44

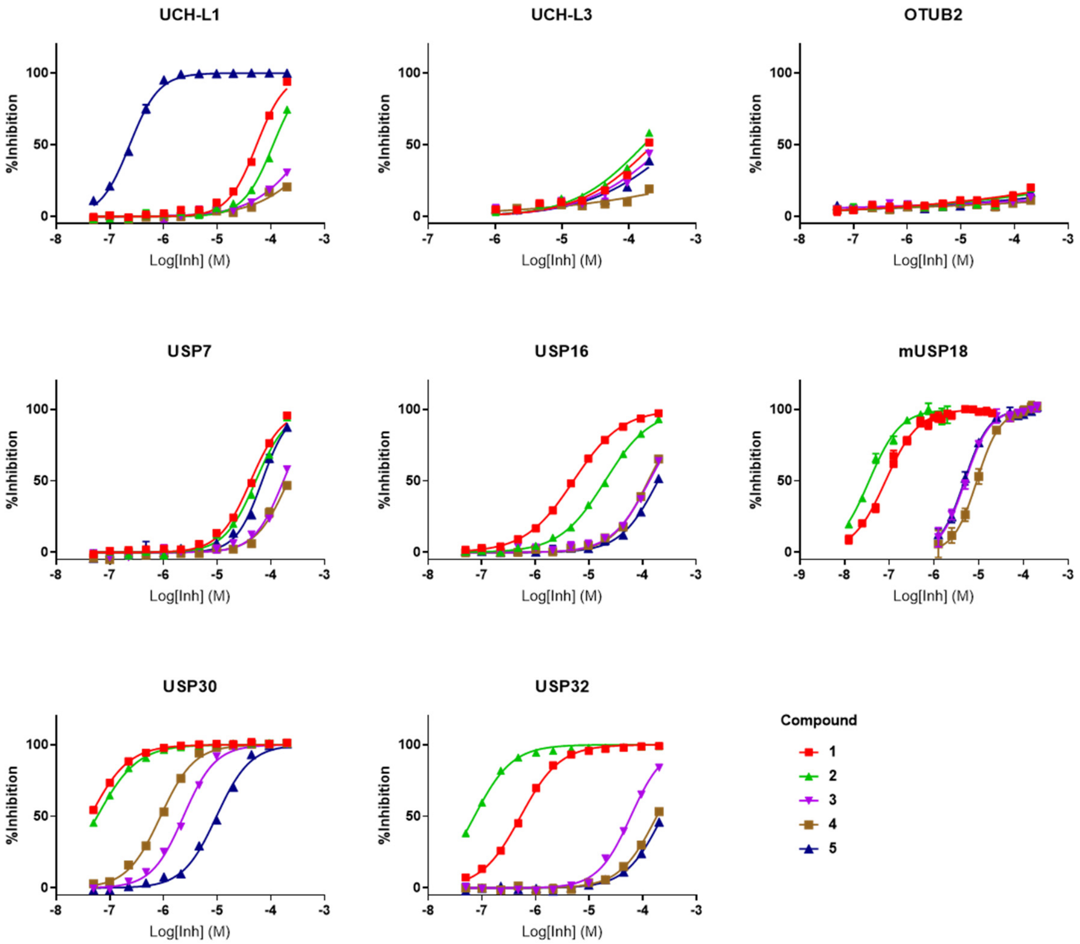

Figure S1. IC$_{50}$ determination of compounds 1-5 for different Ub(like) proteases. Enzymes were pre-incubated with a concentration series of the compounds for 30 minutes at room temperature, before addition of the substrate and direct read-out by fluorescence intensity. IC$_{50}$ values are shown in Figure 1. Data is presented as mean ± SD, *n*=3.

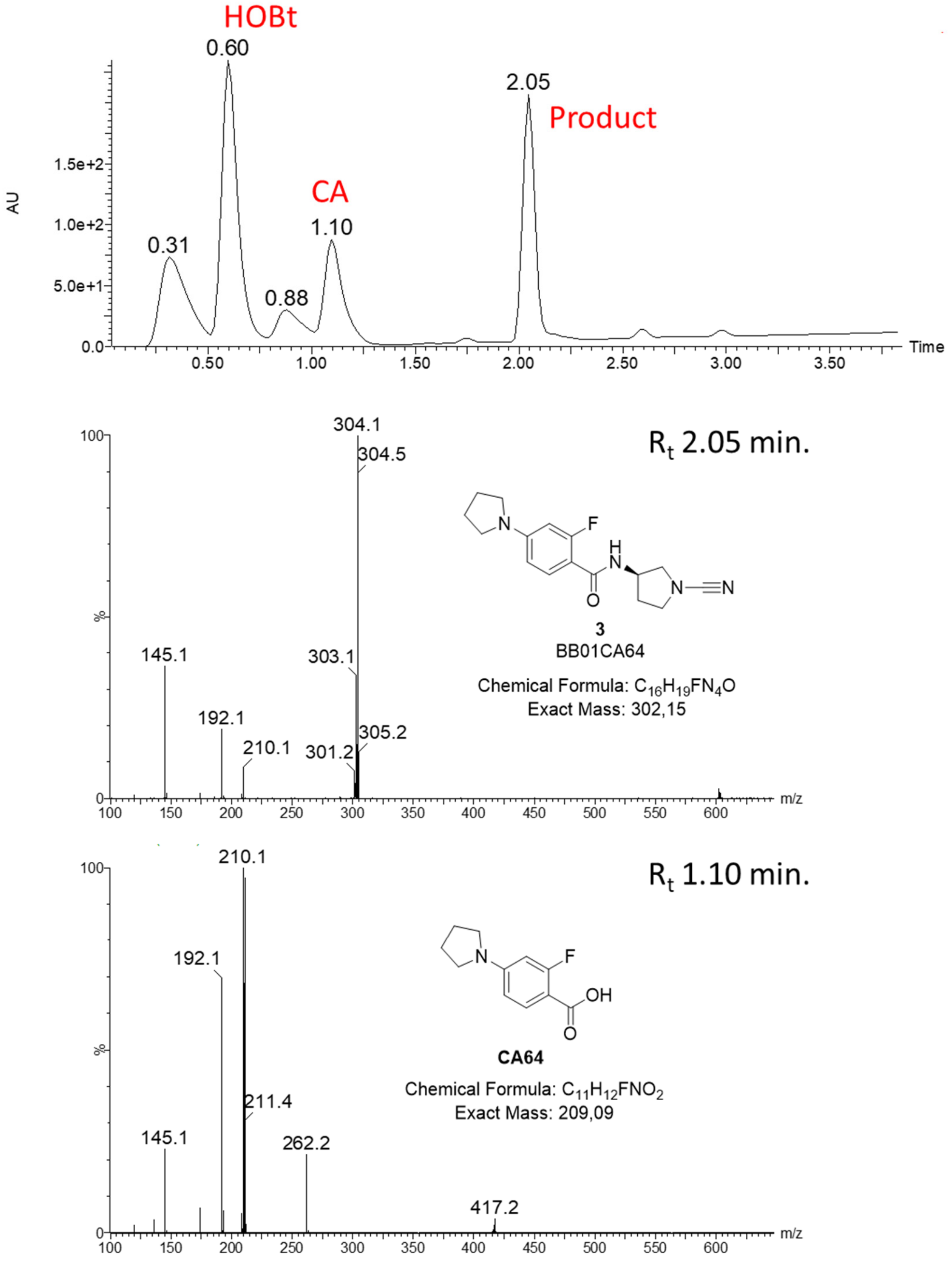

Figure S2. Full spectrum LC-MS analysis of the model reaction shown in Figure 2C,D.

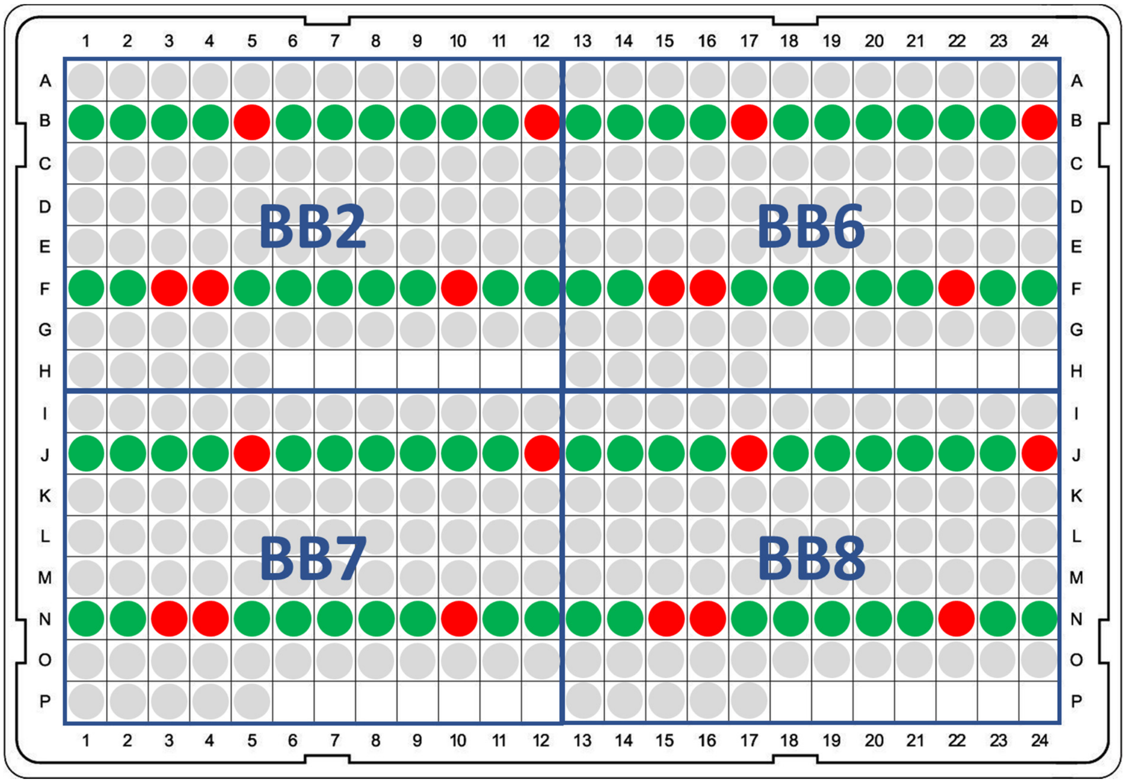

Figure S3. Plate layout and LCMS analysis of the first generation compound library in 384-well format. All compounds from the indicated wells were analyzed. Colors represent product quality. Green: main product formation with (almost) no side products; red: little product formation or no product observed; grey: not analyzed; white: empty wells.

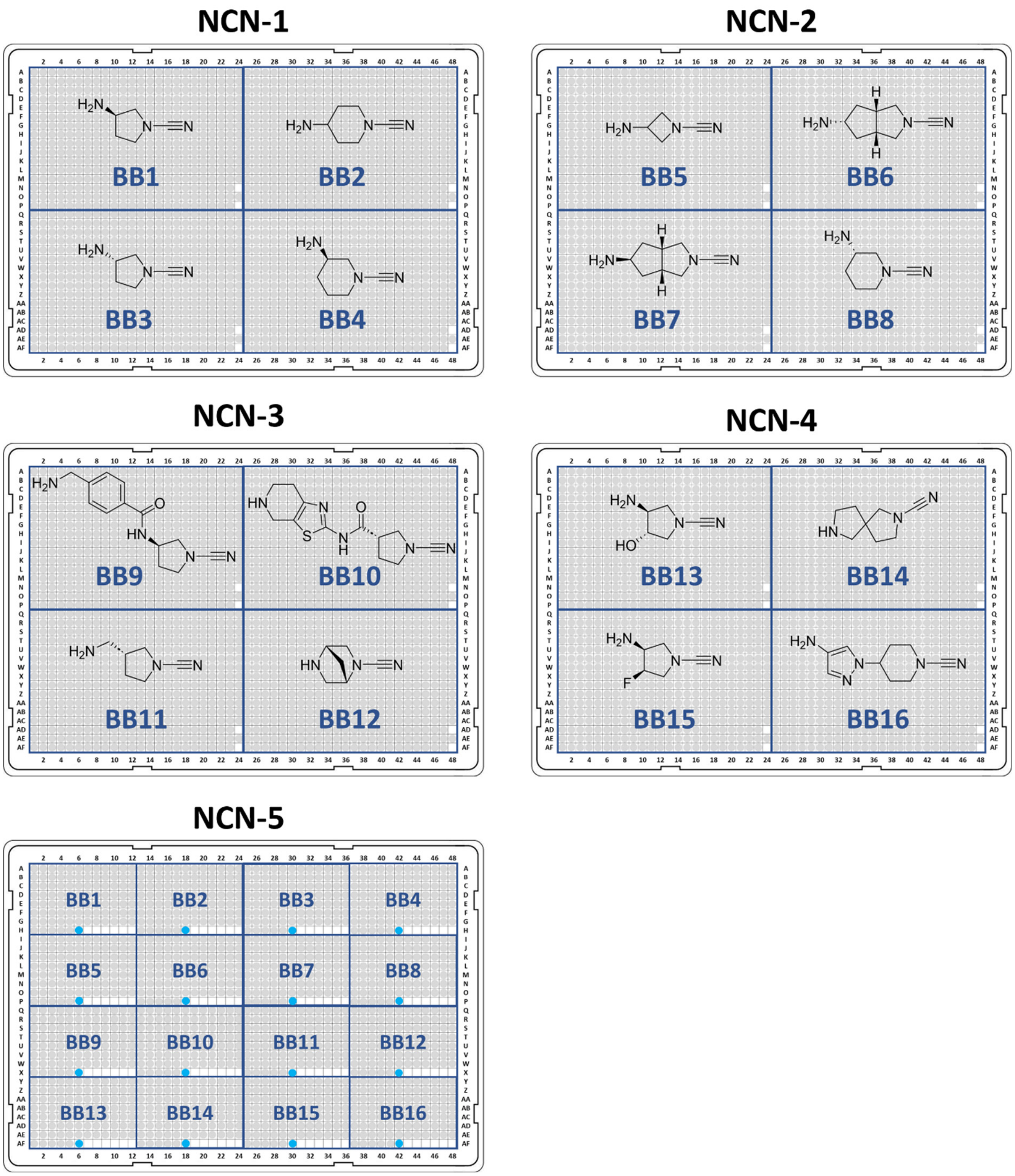

Figure S4. Plate outline of all 1,536-well synthesis plates. Plate numbers are indicated and correspond to the supplementary data file. Locations of amine BB's are indicated. Plates NCN-1-4 were synthesized with 382 Enamine CA's, plate NCN-5 was synthesized with 89 in-house selected CA's. Grey-colored wells contain compounds. White-colored wells are empty. Blue-colored wells contain BB only.

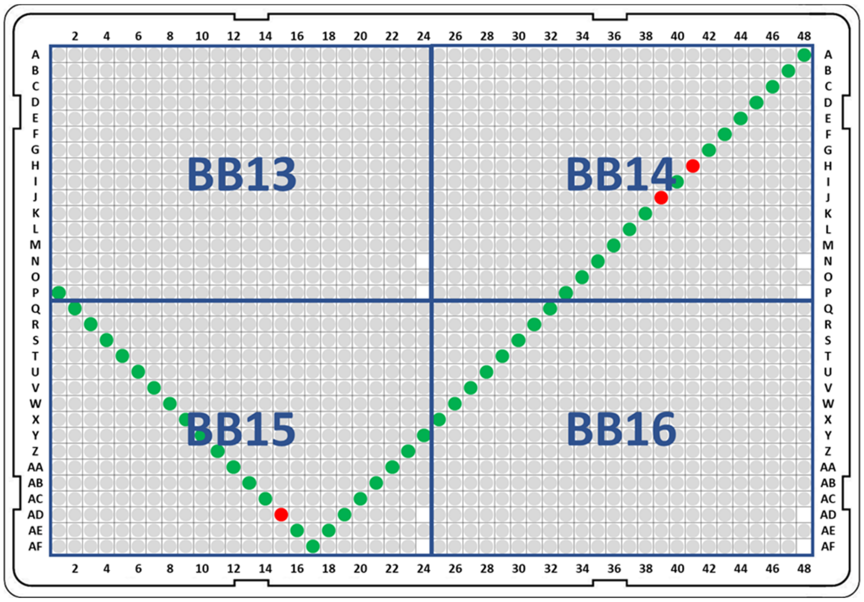

Figure S5. LCMS analysis of the second-generation compound library in 1,536-well format – plate NCN-4. A representative number of compounds covering all rows and columns in the plate were analyzed as indicated. Colors represent product quality. Green: main product formation with (almost) no side products; red: little product formation or no product observed; grey: not analyzed.

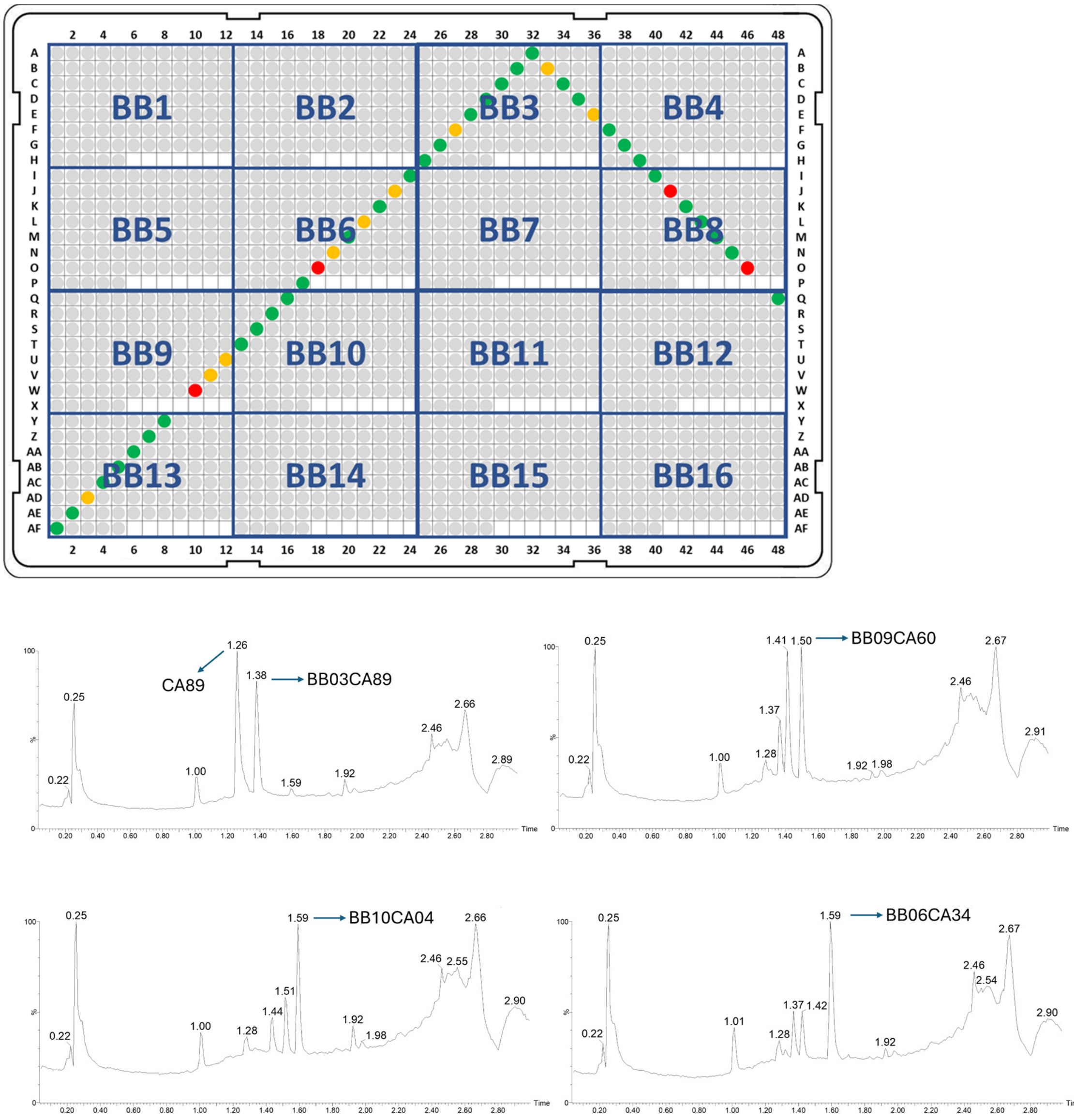

Figure S6. LCMS analysis of the second-generation compound library in 1,536-well format – plate NCN-5. Top: A representative number of compounds covering all rows and columns in the plate were analyzed as indicated. Colors represent product quality. Green: main product formation with (almost) no side products; yellow: medium product formation with some side products observed; red: little product formation or no product observed; grey: not analyzed. Bottom: Examples of LC-MS analysis (UV traces) of four compounds from NCN-5. Compound codes are indicated.

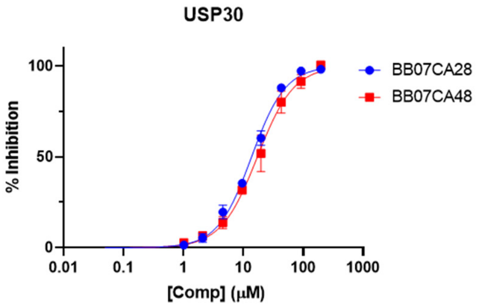

Compound	IC_{50} (μM)
BB07CA28	13.7 ± 0.74
BB07CA48	17.0 ± 1.0

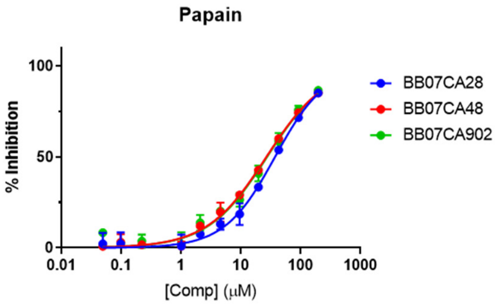

Compound	IC_{50} (μM)
BB07CA28	37.2 ± 2.18
BB07CA48	26.6 ± 1.23
BB07CA902	27.3 ± 1.93

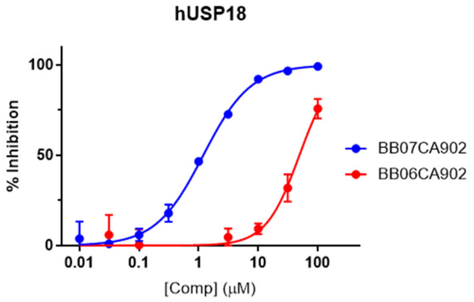

Compound	IC_{50} (μM)
BB07CA902	1.20 ± 0.063
BB06CA902	49.2 ± 3.90

Figure S7. IC_{50} determination of hit compounds for USP30, papain, and hUSP18. Enzymes were pre-incubated with a concentration series of the compounds for 30 minutes at room temperature, followed by the addition of substrate and direct read-out by fluorescence intensity. Data is presented as mean ± SD, *n*=3.

A

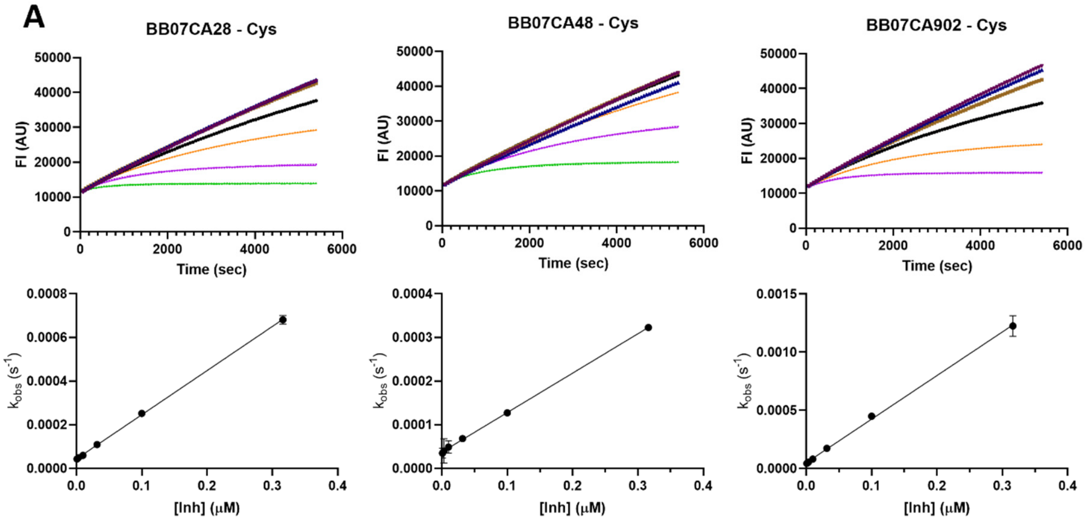

Compound	k_{inact}/K_i ($M^{-1}s^{-1}$)
BB07CA28	2023 ± 15
BB07CA48	903 ± 88
BB07CA902	3752 ± 57

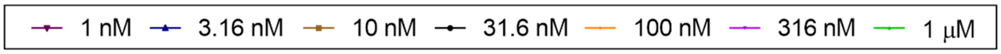

B

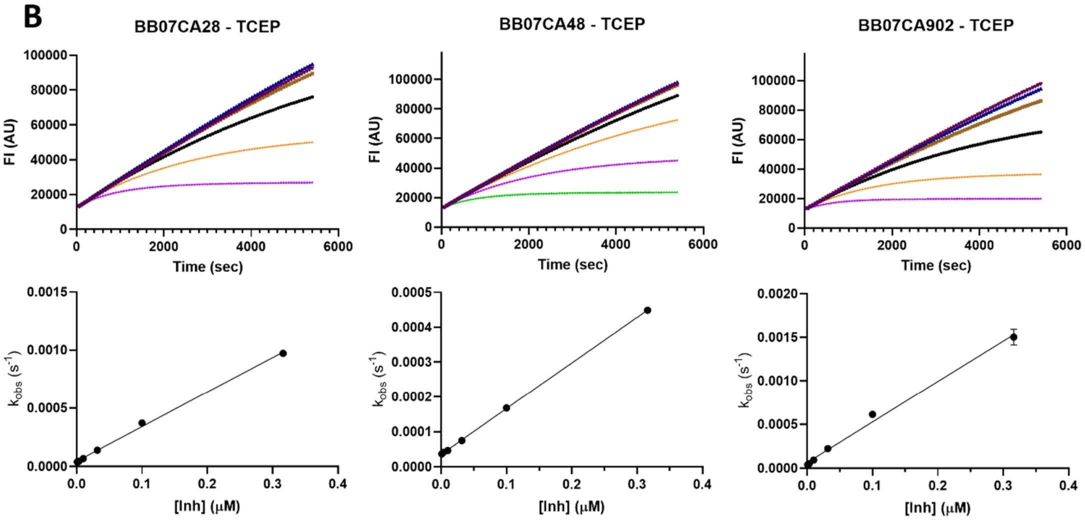

Compound	k_{inact}/K_i ($M^{-1}s^{-1}$)
BB07CA28	2972 ± 63
BB07CA48	1311 ± 64
BB07CA902	4646 ± 182

Figure S8. Determination of k_{inact}/K_i values for hit compounds on mUSP18. The assay was performed in the presence of A) 2 mM cysteine or B) 1 mM TCEP as reducing agent. Data is presented as mean ± SD, *n*=3.

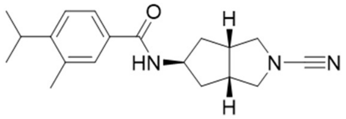

BB07CA902

Chemical Formula: $C_{19}H_{25}N_3O$

Exact Mass: 311,2

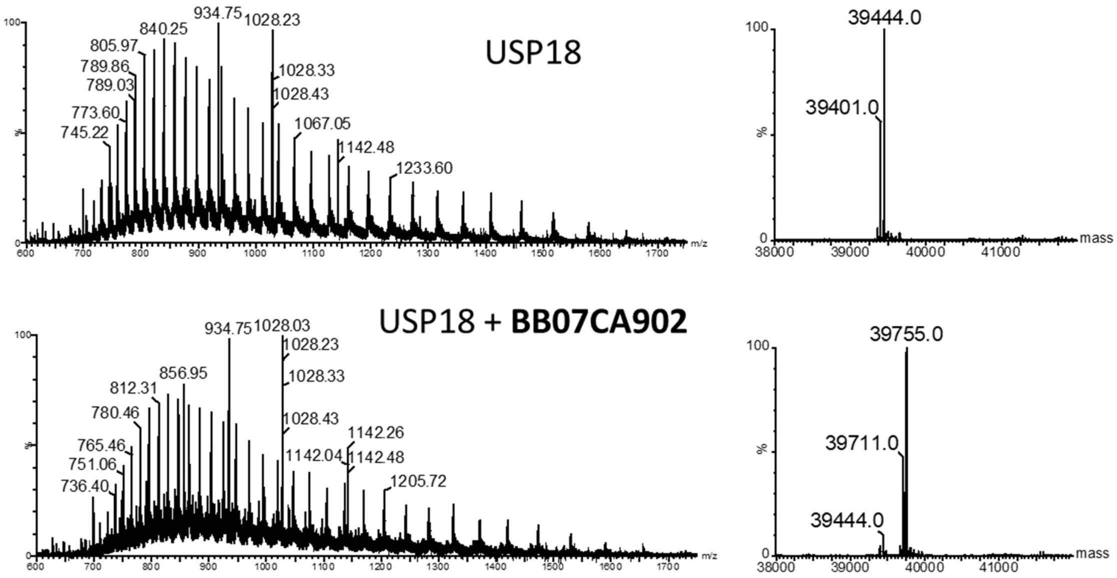

Figure S9. LC-MS analysis confirms formation of a covalent complex of USP18 with one molecule of BB07CA902. Ion traces (left) and deconvoluted mass (right) of USP18 before (top) and after (bottom) incubation with BB07CA902. The mass difference of 311 Da corresponds to the addition of exactly one molecule of BB07CA902. This figure is connected to Figure 5D.

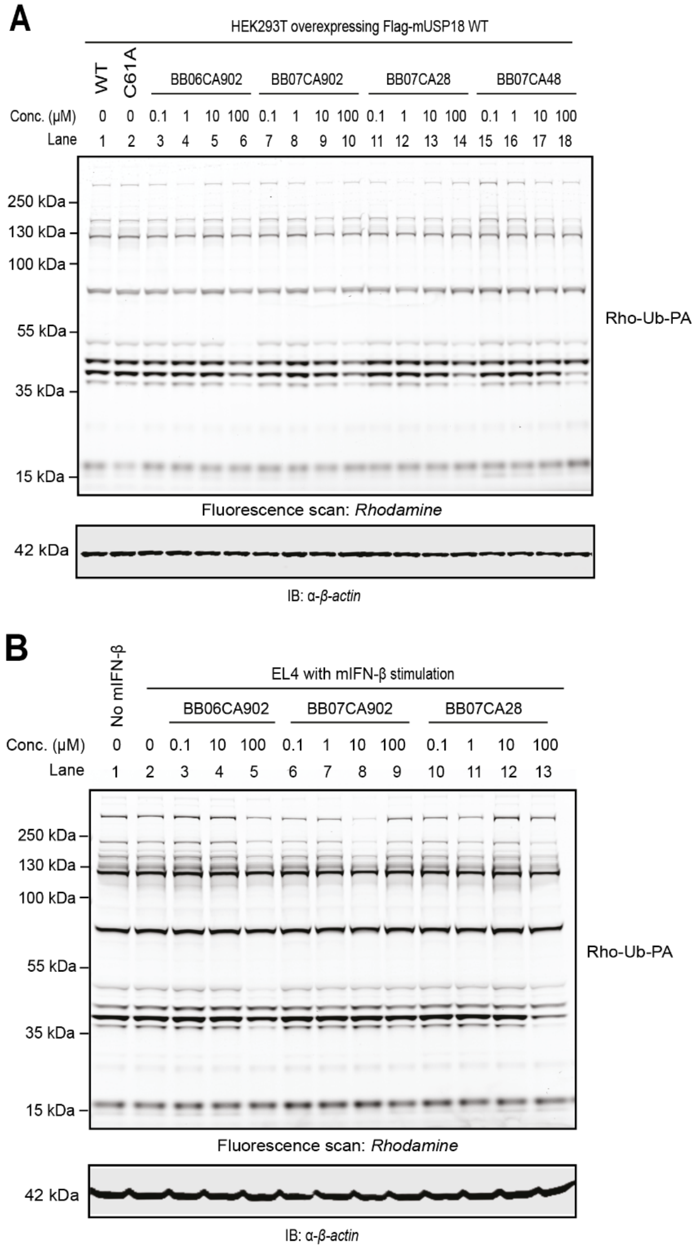

Figure S10. Gel-based ABPP selectivity assessment using Rho-Ub-PA probe. A) ABPP in HEK293 cells overexpressing Flag-mUSP18. At 24 hours following transfection, cells were incubated with DMSO or increasing concentrations of indicated inhibitors for 4 hours, lysed, and treated with Rho-Ub-PA. Top: fluorescence scan of Rho-Ub-PA probe labelled human DUBs. Bottom: anti-β-actin western blot confirming equal loading of each sample. B) ABPP in mIFN-β stimulated mouse EL4 cells. At 24 hours following transfection, cells were incubated with DMSO or increasing concentrations of indicated inhibitors for 4 hours, lysed and treated with Rho-Ub-PA. Top: fluorescence scan of Rho-Ub-PA probe labelled mouse DUBs. Bottom: anti-β-actin western blot confirming equal loading of each sample.

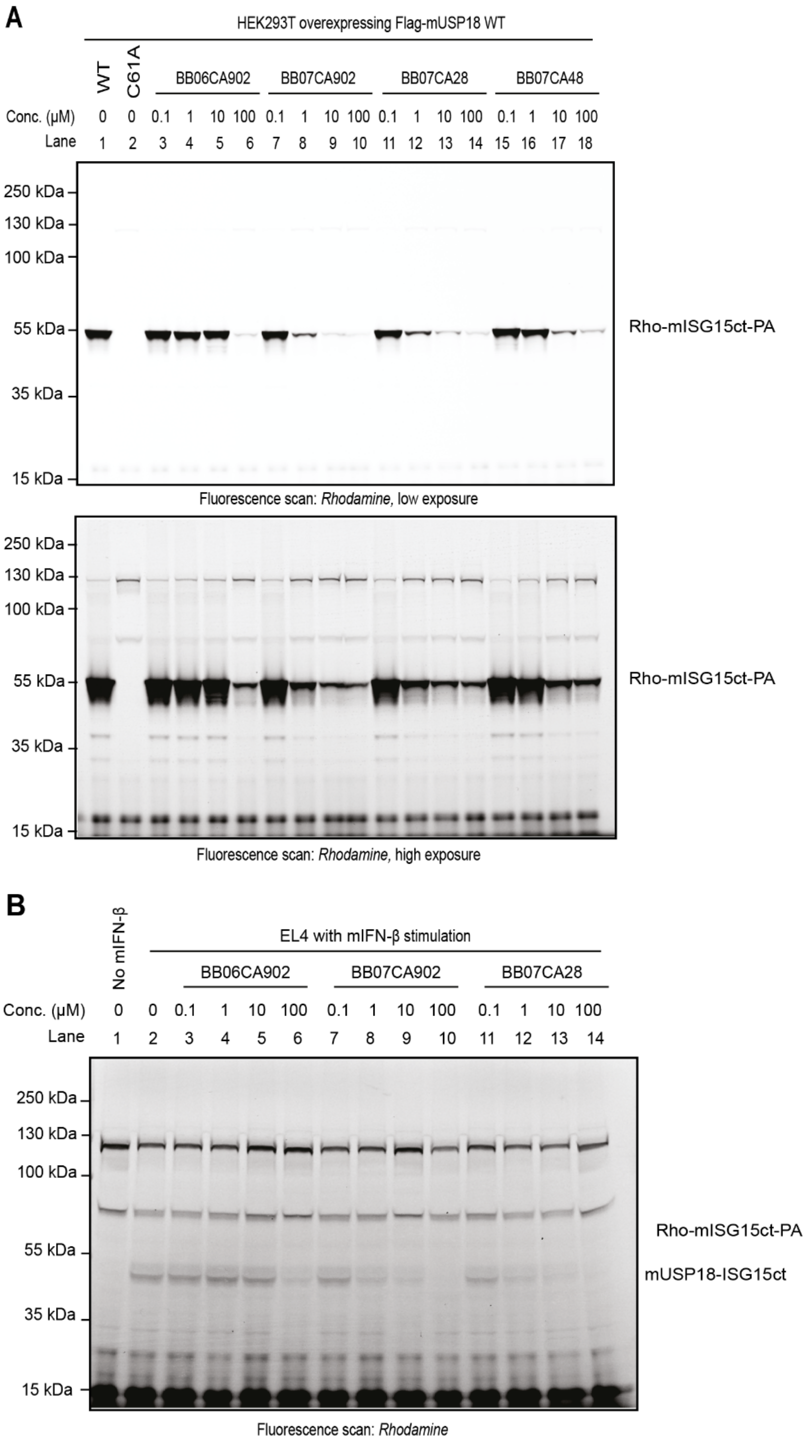

Figure S11. Full image scan of Rho-mISG15ct-PA probe labelled enzymes. A) Gel-based ABPP assessment using a Rho-mISG15ct-PA probe in HEK293 cells overexpressing Flag-mUSP18, corresponding to top panel in Figure 6A. Top panel: fluorescence scan in low exposure; bottom panel: fluorescence scan in high exposure. B) Gel-based ABPP assessment using a Rho-mISG15ct-PA probe in mIFN-β stimulated mouse EL4 cells, corresponding to middle panel in Figure 6B.

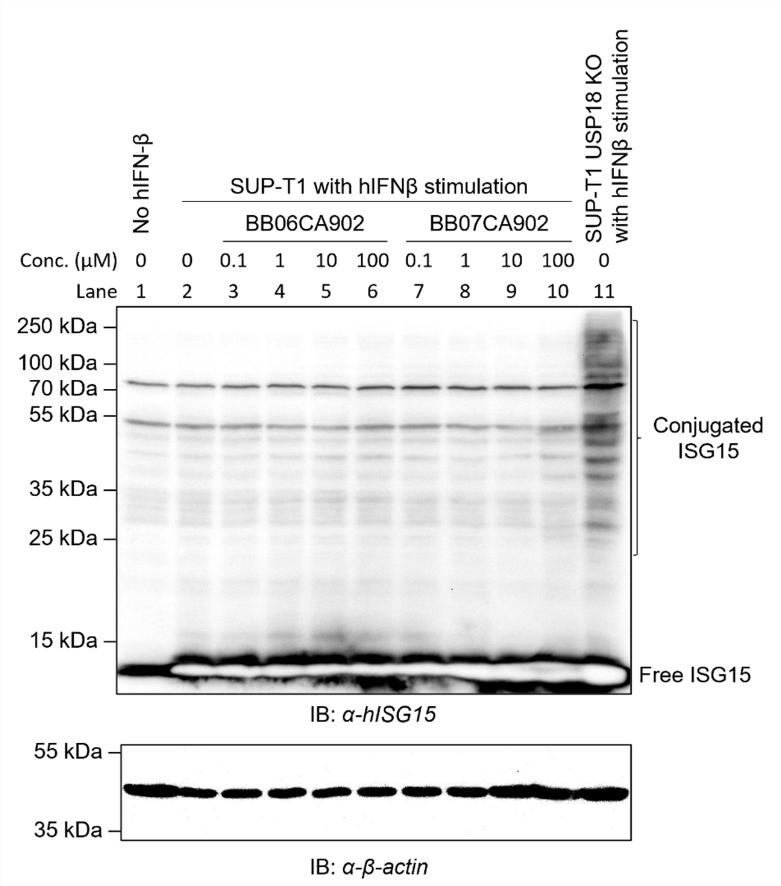

Figure S12. ISGylation in human IFN-β-stimulated SUP-T1 cells is not increased upon compound treatment. At 24 hours following IFN-β treatment, SUP-T1 cells were incubated with DMSO or increasing concentrations of BB07CA902 and BB06CA902 for 4 hours prior to cell lysis. USP18 KO cells show accumulation of ISGylation upon IFN-β stimulation (lane 11). Top panel: anti-hISG15 western blot. Bottom panel: anti-β-actin western blot confirms equal loading.

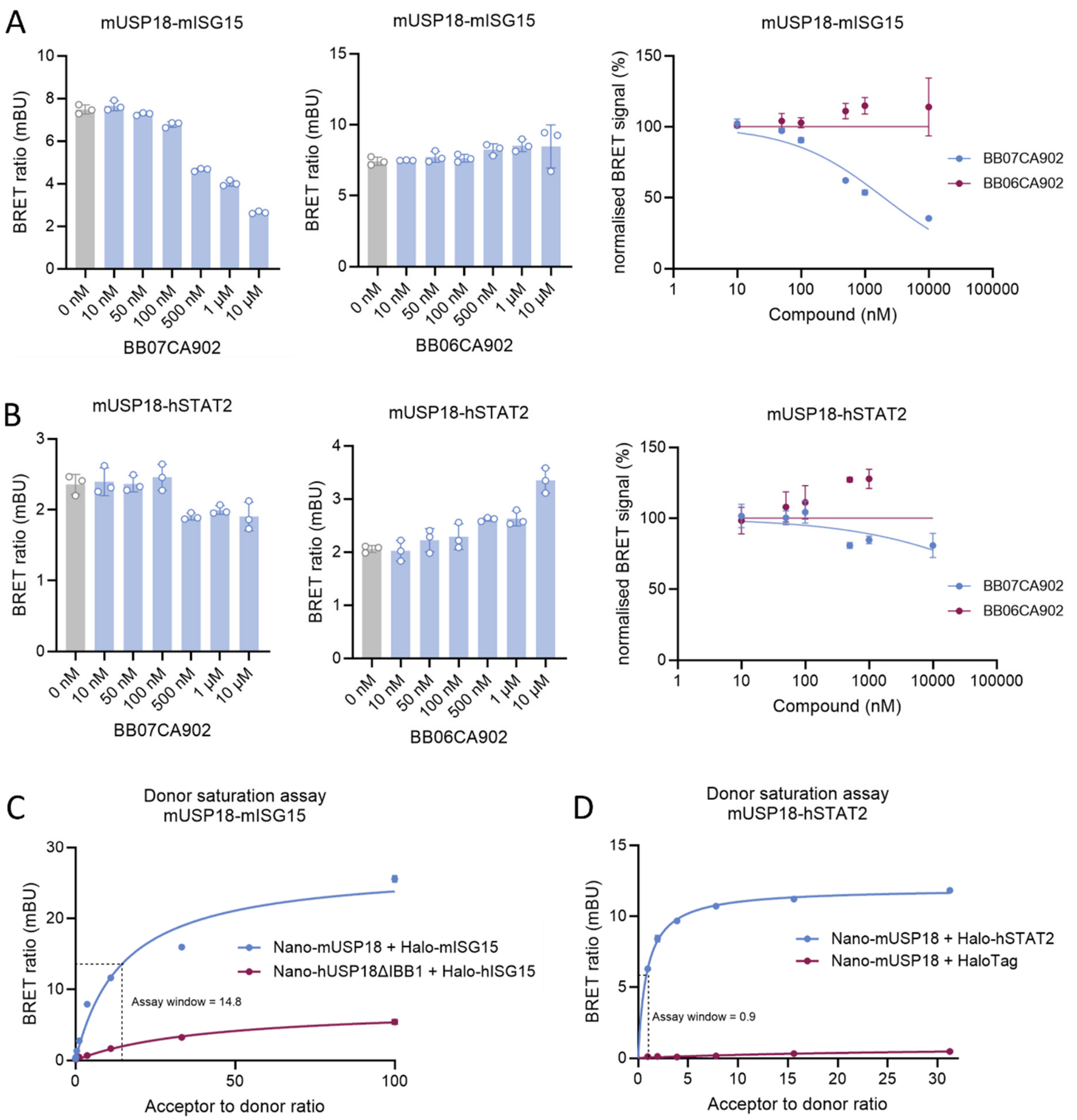

Figure S13. NanoBRET assays to assess the effect of the compounds on the interactions of mUSP18 with ISG15 and STAT2. BRET ratio of A) murine USP18/ISG15 interaction, and B) murine USP18/human STAT2 interaction upon treatment with different concentrations of inhibitor BB07CA902 and control compound BB06CA902. BRET ratios were normalised to the DMSO control and inhibition was assessed by fitting the data to a dose-response model. C) Donor saturation assay for the USP18/ISG15 protein pair using nano-hUSP18ΔIBB1 as a negative control. D) Donor saturation assay for the USP18/STAT2 protein pair using unfused HaloTag as a negative control. Curve fitting was performed using a one-site specific binding model. Data are shown as mean ± SD from one biological replicate with three technical replicates.

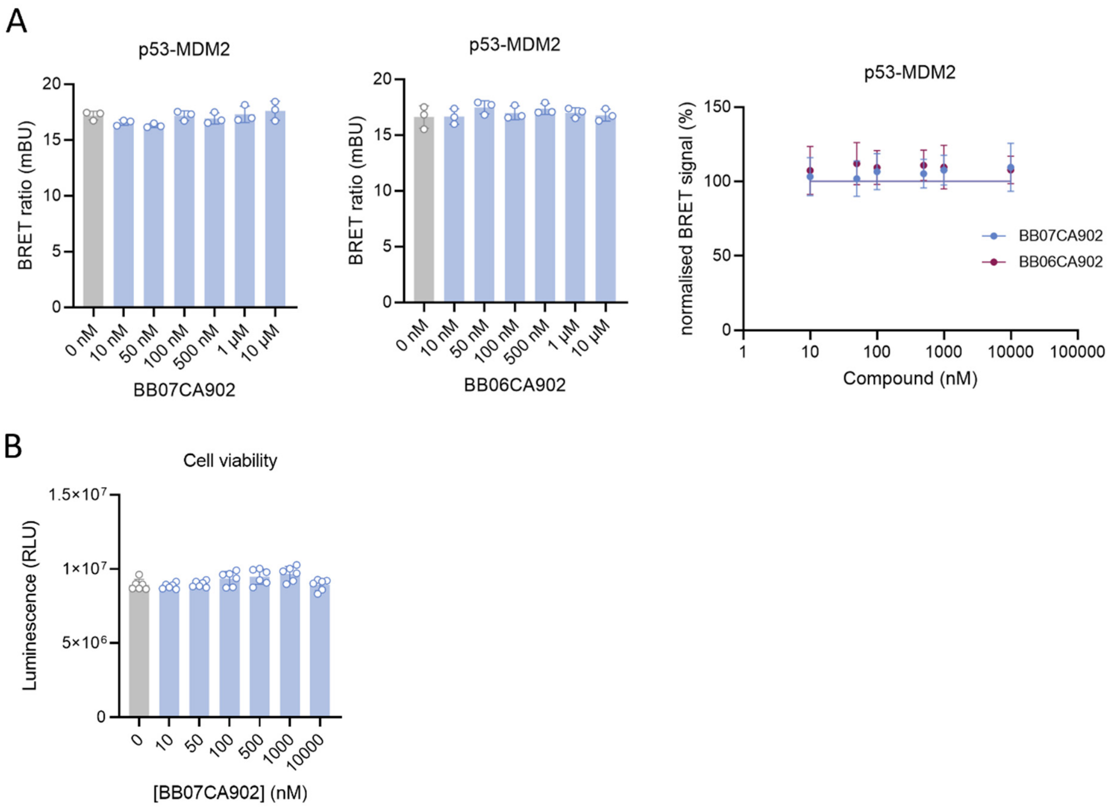

Figure S14. NanoBRET control assay to assess the effect of the compounds on the interaction of p53 with MDM2, and cell viability assay. A) BRET ratio of p53/MDM2 interaction upon treatment with different concentrations of inhibitor BB07CA902 and control compound BB06CA902. BRET ratios were normalised to the DMSO control and inhibition was assessed by fitting the data to a dose-response model. B) Cell viability assay with inhibitor BB07CA902 in HEK293T cells. Data are shown as mean ± SD from one biological replicate with three technical replicates.

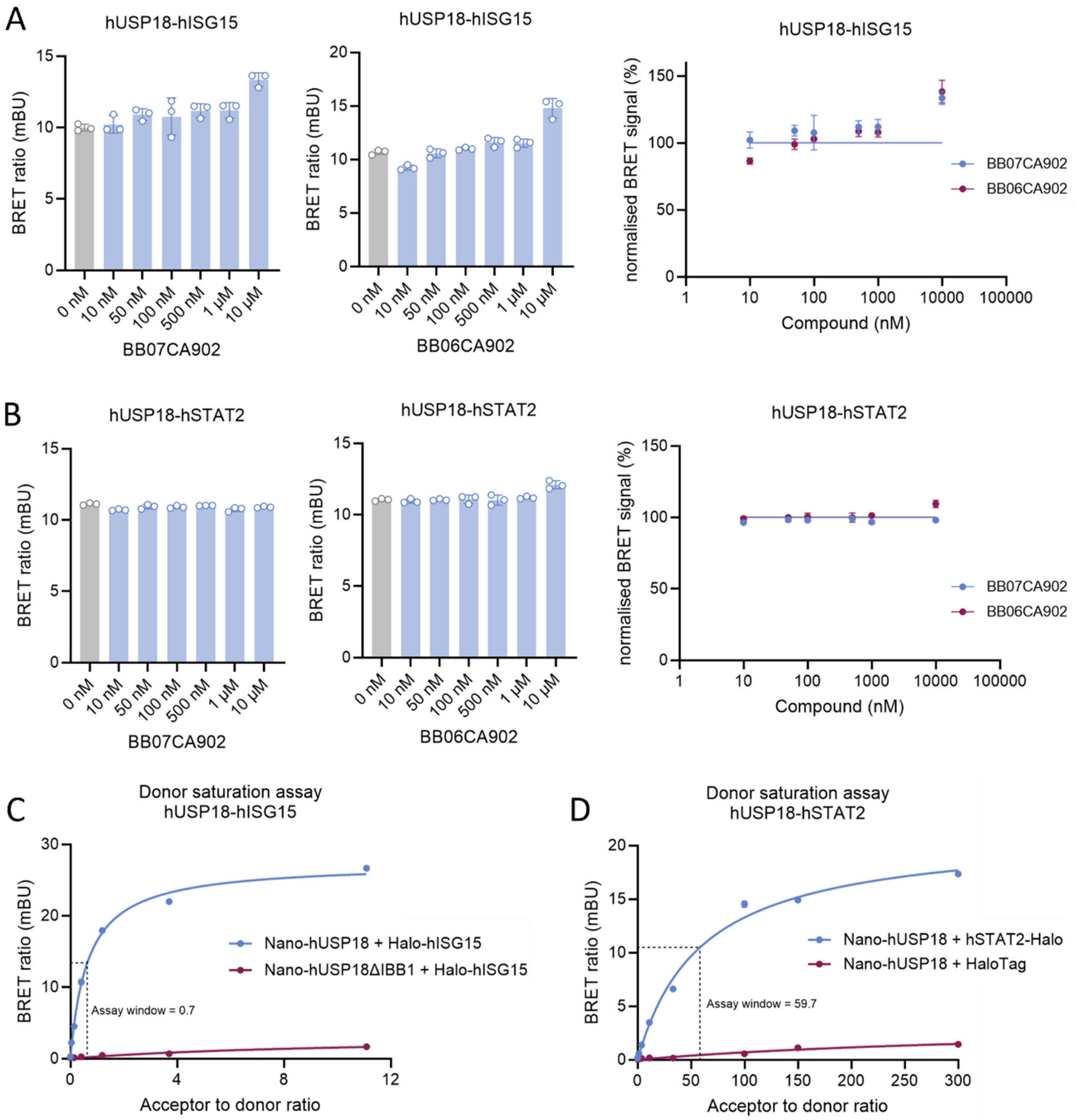

Figure S15. NanoBRET assays to assess the effect of the compounds on the interactions of hUSP18 with ISG15 and STAT2. BRET ratio of A) human USP18/ISG15 interaction, and B) human USP18/STAT2 interaction upon treatment with different concentrations of inhibitor BB07CA902 and control compound BB06CA902. BRET ratios were normalised to the DMSO control and inhibition was assessed by fitting the data to a dose-response model. C) Donor saturation assay for the USP18/ISG15 protein pair using nano-hUSP18ΔIBB1 as a negative control. D) Donor saturation assay for the USP18/STAT2 protein pair using unfused HaloTag as a negative control. Curve fitting was performed using a one-site specific binding model. Data are shown as mean ± SD from one biological replicate with three technical replicates.

Synthesis

General

Reagents, solvents and solutions. General reagents were purchased from Sigma-Aldrich, Acros and Combi-Blocks, Inc. and were used as received. Solvents were purchased from VWR, Biosolve or Sigma-Aldrich. Thin Layer Chromatography (TLC) was performed on Merck aluminum sheets (pre-coated with silica gel 60 F_{254}). Compounds were visualized by UV adsorption (254 nm) and by using a solution of $KMnO_4$ (7.5 g L^{-1}) and K_2CO_3 (50 g L^{-1}) in H_2O or a solution of ninhydrin (15 g L^{-1}) in 3% AcOH/EtOH v/v.

Purification Techniques. Compounds (unless stated otherwise) were purified on a Büchi Sepacore automatic flash chromatography system X10/X50. The Büchi Sepacore system was equipped with two Büchi pump modules C-605, a Büchi control unit C-620, Büchi fraction collector C-660 and a Büchi UV Photometer C-640. The silica columns were purchased at GraceResolv™ and were packed with a grade of Davisil® silica.

Instrumentation for Compound Characterization. NMR spectra (^1H, ^{13}C) were recorded on a Bruker Ultrashield 300 MHz spectrometer at 298 K. Resonances are indicated with symbols 'd' (doublet), 's' (singlet), 't' (triplet) and 'm' (multiplet). Chemical shifts (δ) are given in ppm relative to $CDCl_3$, DMSO-d_6 or CD_3OD as an internal standard and coupling constants (*J*) are quoted in hertz (Hz). LC-MS measurements were performed on an LC-MS system equipped with a Waters 2795 Separation Module (Alliance HT), a Waters 2996 Photodiode Array Detector (190–750 nm), an Xbridge C18 column (2.1 × 100 mm, 3.5 μm) and an LCT ESI-Orthogonal Acceleration Time of Flight Mass Spectrometer. Samples were run using 2 mobile phases: A = 1% CH_3CN and 0.1% formic acid in H_2O and B = 1% H_2O and 0.1% formic acid in CH_3CN. Data processing was performed using Waters MassLynx Mass Spectrometry Software 4.1. LC-MS Program: Waters Xbridge C18 column (2.1 × 100 mm, 3.5 μm); flow rate = 0.4 mL min^{-1}, runtime = 13 min, column T = 40 °C, mass detection: 100–1500 Da. Gradient: 0–0.4 min: 5% B; 0.4–9.0 min: 5% → 95% B; 9.0–11.2 min: 95% B; 11.2–11.3 min: 95% → 5% B; 11.3–13.00 min: 5% B. Electrospray Ionization (ESI) high-resolution mass spectrometry was carried on a Waters XEVO-G2 XS Q-TOF mass spectrometer equipped with an electrospray ion source in positive mode (capillary voltage: 3.0 kV, desolvation gas flow: 900 L h^{-1}, temperature: 60 °C) with a resolution R = 22,000 using 200 pg μL^{-1} Leu-Enk (*m/z* = 556.2771) as a "lock mass". Samples were run using 2 mobile phases: A = 0.1% formic acid in H_2O and B = 0.1% formic acid in CH_3CN on a Waters Acquity UPLC BEH C18 column (2.1 × 50 mm, 1.7 μm); flow rate = 0.6 mL min^{-1}, runtime = 3.00 min, column T = 60 °C, mass

detection: 50–1500 Da. Gradient: 0–0.15 min: 2% B; 0.15–1.85 min: 2% → 100% B; 1.85–2.05: 100% B; 2.05–2.10 min: 100% → 2% B; 2.10–3.00 min: 100% B. Data processing was performed using Waters MassLynx Mass Spectrometry Software 4.1.

Synthesis of ISG15-C-terminal domain-Rhodamine-morpholine (ISG15$_{CT}$-Rho-MP)

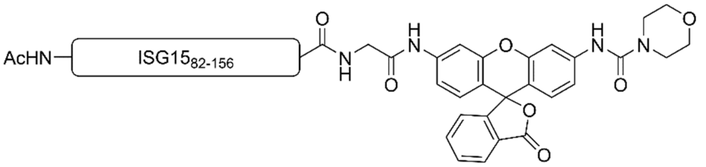

Protected Ac-ISG15$_{82-156}$ (20 µmol) was synthesized by Fmoc SPPS on trityl-resin as described.[1] It was dissolved in DMF (7 mL) and to this were added EDC.HCl (11.5 mg, 60 µmol 3.0 eq.), HOBt (8 mg, 60 µmol, 3.0 eq.) and Gly-Rho-morpholine[2] (30 mg, 60 µmol, 3.0 eq.) and the mixture was stirred at rt for 5 hours before being concentrated to dryness under reduced pressure. The resulting residue was dissolved in a mixture of 90.5/5/2/2.5 v/v/v/v TFA/H_2O/TIPS/phenol (7 mL) and stirred for 3 hours at rt. The protein was precipitated from ice-cold Et_2O/*n*-pentane (1/1; v/v; 30 mL). The solution was centrifuged (5 min. at 1500 rpm) and the supernatant was removed by decanting. The pellet was washed with Et_2O (20 mL), the solution was vortexed, the suspension was centrifuged and Et_2O was removed by decanting. The wash step was repeated twice. The pellet was dissolved in H_2O/CH_3CN/AcOH (65/25/10; v/v/v; 15 mL) and lyophilized. The protein was subsequently purified using RP-HPLC and the product was obtained as a white solid (55.4 mg, 6.1 µmol, 31%). LC-MS (10 min. run): R_t (min.) 3.90; deconvoluted mass: 9008.0.

(*R*)-3-(3-chlorophenyl)-*N*-(1-cyanopyrrolidin-3-yl)isoxazole-5-carboxamide (1)

Synthesized as described in literature.[3]

^{1}H NMR (300 MHz, DMSO-*d*$_6$) δ 9.31 (d, *J* = 6.5 Hz, 1H), 8.00 (t, *J* = 1.9 Hz, 1H), 7.91 (dt, *J* = 7.0, 1.7 Hz, 1H), 7.65 – 7.54 (m, 2H), 4.56 – 4.42 (m, 1H), 3.65 (dd, *J* = 9.8, 6.4 Hz, 1H), 3.56 (dt, *J* = 9.1, 7.4 Hz, 1H), 3.46 (ddd, *J* = 9.0, 8.0, 5.4 Hz, 1H), 3.39 – 3.31 (m, 2H), 2.22 – 2.06 (m, 1H), 1.97 (ddt, *J* = 12.7, 7.5, 5.2 Hz, 1H). HR-MS calculated for $C_{15}H_{13}N_4O_2Cl$ $[M+H]^+$ 317.0805, found 317.0797.

(*R*)-*N*-(1-cyanopyrrolidin-3-yl)-5-(2-methoxyphenyl)-1*H*-pyrazole-3-carboxamide (2)

Synthesized as described in literature.[3]

^{1}H NMR (300 MHz, DMSO-*d*$_6$) δ 13.33 (s, 1H), 8.39 (s, 1H), 7.72 (s, 1H), 7.47 – 7.28 (m, 1H), 7.15 (d, *J* = 8.3 Hz, 1H), 7.04 (t, *J* = 7.4 Hz, 2H), 4.50 (td, *J* = 6.7, 5.3 Hz, 1H), 3.90 (s, 3H), 3.68 – 3.50 (m, 2H), 3.44 (ddd, *J* = 9.0, 7.6, 6.4 Hz, 1H), 3.38 – 3.29 (m, 2H), 2.18 – 2.05 (m, 1H), 2.05 – 1.92 (m, 1H). HRMS calculated for $C_{16}H_{17}N_5O_2$ $[M+H]^+$ 312.1461, found 312.1428.

(*R*)-*N*-(1-cyanopyrrolidin-3-yl)-2-fluoro-4-(pyrrolidin-1-yl)benzamide (3)

Synthesized as described in literature.[3]

^{1}H NMR (300 MHz, DMSO-*d*$_6$) δ 8.56 (d, *J* = 6.4 Hz, 1H), 7.21 – 7.12 (m, 1H), 6.52 – 6.34 (m, 2H), 4.35 (tdd, *J* = 6.2, 4.1, 2.0 Hz, 1H), 3.58 (dd, *J* = 9.8, 6.0 Hz, 1H), 3.53 – 3.39 (m, 2H), 3.33 (s, 1H), 3.28 (ddd, *J* = 9.8, 3.9, 0.8 Hz, 1H), 3.22 – 3.12 (m, 4H), 2.08 (dtd, *J* = 12.7, 8.0, 6.1 Hz, 1H), 1.87 (td, *J* = 6.7, 5.9, 2.5 Hz, 5H). HRMS calculated for $C_{16}H_{19}N_4OF$ $[M+H]^+$ 303.1621, found 303.1615.

(*R*)-*N*-(1-cyanopyrrolidin-3-yl)-3,4-difluorobenzamide (4)

Synthesized as described in literature.[3]

^{1}H NMR (300 MHz, DMSO-*d*$_6$) δ 8.71 (s, 1H), 7.92 (ddd, *J* = 11.7, 7.8, 2.2 Hz, 1H), 7.76 (dddd, *J* = 8.2, 4.6, 2.2, 1.3 Hz, 1H), 7.57 (dt, *J* = 10.5, 8.3 Hz, 1H), 4.46 (tt, *J* = 6.3, 4.5 Hz, 1H), 3.63 (dd, *J* = 9.8, 6.4 Hz, 1H), 3.55 (dt, *J* = 9.1, 7.4 Hz, 1H), 3.45 (ddd, *J* = 9.1, 8.0, 5.3 Hz, 1H), 3.31 (dd, *J* = 9.8, 4.1 Hz, 1H), 2.22 – 2.05 (m, 1H), 1.94 (ddt, *J* = 12.6, 7.4, 5.1 Hz, 1H). HRMS calculated for $C_{12}H_{11}N_3OF_2$ $[M+H]^+$ 252.0948, found 252.0952.

(*S*)-1-cyano-*N*-(5-morpholinothiazol-2-yl)pyrrolidine-3-carboxamide (5)

Synthesized as described in literature.[4]

^{1}H NMR (300 MHz, DMSO-*d*$_6$) δ 11.96 (s, 1H), 6.68 (s, 1H), 3.75 – 3.66 (m, 4H), 3.59 (dd, *J* = 9.5, 7.8 Hz, 1H), 3.52 – 3.35 (m, 3H), 3.31 – 3.20 (m, 1H), 3.02 – 2.94 (m, 4H), 2.22 – 2.09 (m, 1H), 2.09 – 1.95 (m, 1H). HRMS calculated for $C_{13}H_{17}N_5O_2S$ $[M+H]^+$ 308.1181, found 308.1170.

General procedure for the synthesis of cyanimide building blocks (BB1-16)

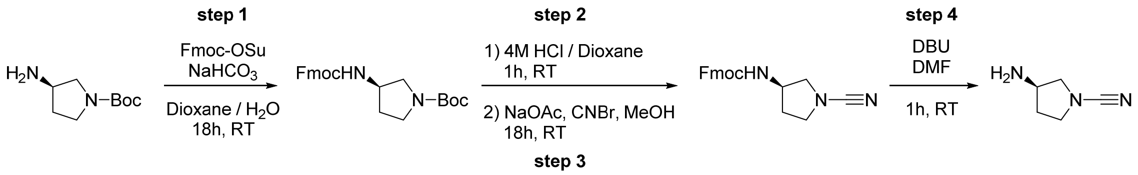

Step 1: Fmoc protection. Boc-protected diamine (500 mg, 1.0 eq.) was suspended in H_2O (18 mL) and Fmoc-OSu (1.1 eq.) and $NaHCO_3$ (2.0 eq.) were added. Dioxane (9 mL) was added and the resulting reaction mixture was stirred at rt for 18h. TLC analysis indicated complete consumption of the starting material with formation of a less polar product. The reaction mixture was diluted with H_2O (22 mL) and EtOAc (40 mL) was added. The layers were separated and the organic layer was washed with H_2O (1 x 20 mL) and brine (1 x 20

mL). The organic layer was dried over Na_2SO_4, filtered and concentrated *in vacuo* to obtain orthogonally protected diamine.

Step 2: Boc deprotection. Orthogonally protected diamine (1.0 eq.) was treated with 4M HCl / Dioxane (30 mL) and the resulting reaction mixture was stirred at rt for 1h. TLC analysis indicated complete consumption of the starting material with formation of a more polar product. The reaction mixture was concentrated *in vacuo* and the remaining residue was co-concentrated with DCM (3 x 30 mL) to yield mono-Fmoc protected diamine as the HCl salt.

Step 3: Cyanimide synthesis. Mono-Fmoc protected diamine (HCl salt) (1.0 eq) was dissolved in MeOH (15 mL) and NaOAc (5.0 eq.) and cyanogen bromide (4.0 eq.) were added. The resulting reaction mixture was stirred at rt for 18h. The reaction mixture was concentrated *in vacuo* and the crude residue was taken up in EtOAc (50 mL). The organic layer was washed with H_2O (1 x 25 mL) and brine (1 x 25 mL). The organic layer was dried over Na_2SO_4, filtered and concentrated *in vacuo*. The product was isolated *via* Büchi flash chromatography (Heptane → EtOAc) to yield the mono-Fmoc protected cyanimide.

Step 4: Fmoc deprotection. Mono-Fmoc protected cyanimide (1.0 eq.) was dissolved in DMF (140 mM) and 1,8-Diazabicyclo[5.4.0]undec-7-ene (0.5 eq.) was added. The resulting reaction mixture was stirred at rt for 1h until TLC analysis indicated complete consumption of the starting material with formation of a more polar product. The product was isolated *via* Büchi flash chromatography (DCM → 5% MeOH, 0.7M NH_3) to yield the cyanimide building block.

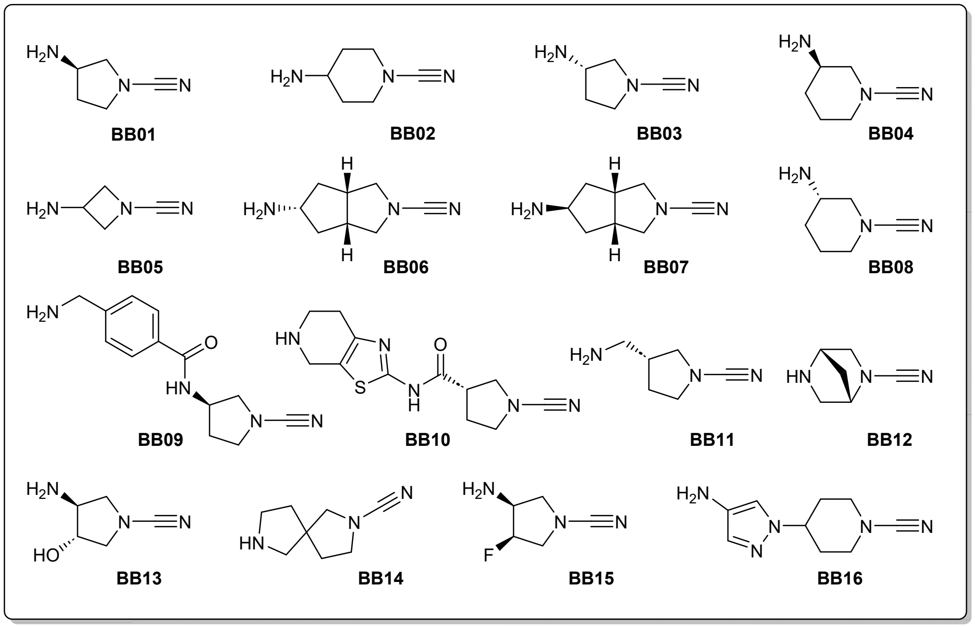

BB1: (*R*)-3-Aminopyrrolidine-1-carbonitrile was synthesized according to the general procedure for cyanimide building block synthesis starting from (*R*)-(+)-1-Boc-3-aminopyrrolidine. ^{1}H NMR (300 MHz, $CDCl_3$) δ 3.63 – 3.51 (m, 2H), 3.47 (dd, *J* = 9.5, 5.7 Hz, 1H), 3.43 – 3.34 (ddd, *J* = 9.2, 8.1, 5.5 Hz, 1H), 3.03 (ddd, *J* = 9.5, 4.2, 0.8 Hz, 1H), 2.08 – 1.93 (m, 1H), 1.70 – 1.57 (m, 1H), 1.26 (s, 2H). ^{13}C NMR (75 MHz, $CDCl_3$) δ 117.6, 58.3, 51.3, 49.0, 34.6. HR-MS calculated for $C_5H_9N_3$ [M+H]$^+$ 112.0875, found 112.0895.

BB2: 4-Aminopiperidine-1-carbonitrile was synthesized according to the general procedure for cyanimide building block synthesis starting from 1-Boc-4-aminopiperidine. ^{1}H NMR (300 MHz, $CDCl_3$) δ 3.38 (dt, *J* = 13.0, 4.0 Hz, 2H), 3.05 – 2.91 (m, 2H), 2.83 – 2.67 (m, 1H), 1.79 (dd, *J* = 13.2, 3.6 Hz, 2H), 1.49 – 1.31 (m, 4H). ^{13}C NMR (75 MHz, $CDCl_3$) δ 118.3, 48.3, 47.1, 34.2. HR-MS calculated for $C_6H_{11}N_3$ [M+H]$^+$ 126.1031, found 126.1048.

BB3: (*S*)-3-Aminopyrrolidine-1-carbonitrile was synthesized according to the general procedure for cyanimide building block synthesis starting from (*S*)-(-)-1-Boc-3-aminopyrrolidine. ^{1}H NMR (300 MHz, $CDCl_3$) δ 3.68 – 3.35 (m, 4H), 3.05 (dt, *J* = 9.4, 4.4 Hz, 1H), 2.03 (dt, *J* = 12.8, 6.1 Hz, 1H), 1.66 (tt, *J* = 7.5, 4.3 Hz, 1H), 1.29 (s, 2H). ^{13}C NMR (75 MHz, $CDCl_3$) δ 117.7, 58.4, 51.4, 49.1, 34.7. HR-MS calculated for $C_5H_9N_3$ [M+H]$^+$ 112.0875, found 112.0872.

BB4: (*R*)-3-Aminopiperidine-1-carbonitrile was synthesized according to the general procedure for cyanimide building block synthesis starting from (*R*)-*tert*-butyl 3-aminopiperidine-1-carboxylate. ^{1}H NMR (300 MHz, $CDCl_3$) δ 3.28 – 3.03 (m, 2H), 2.84 – 2.72 (m, 2H), 2.58 – 2.48 (m, 1H), 1.82 – 1.41 (m, 3H), 1.22 (s, 2H), 1.12 – 0.91 (m, 1H). ^{13}C NMR (75 MHz, $CDCl_3$) δ 118.2, 56.8, 49.2, 46.4, 32.5, 22.8. HR-MS calculated for $C_6H_{11}N_3$ [M+H]$^+$ 126.1031, found 126.1033.

BB5: 3-Aminoazetidine-1-carbonitrile was synthesized according to the general procedure for cyanimide building block synthesis starting from 1-Boc-3-aminoazetidine. ^{1}H NMR (300 MHz, $CDCl_3$) δ 4.29 – 4.18 (m, 2H), 3.90 – 3.75 (m, 3H), 1.63 (s, 2H). ^{13}C NMR (75 MHz, $CDCl_3$) δ 117.8, 65.1, 45.1. HR-MS calculated for $C_4H_7N_3$ [M+H]$^+$ 98.0718, found 98.0724.

BB6: (3a*R*,5*r*,6a*S*)-5-aminohexahydrocyclopenta[*c*]pyrrole-2(1*H*)-carbonitrile.

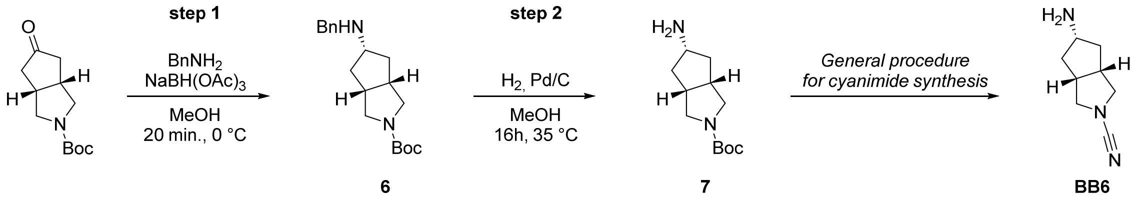

Step 1: *tert*-Butyl (3a*R*,5*r*,6a*S*)-5-(benzylamino)hexahydrocyclopenta[*c*]pyrrole-2(1*H*)-carboxylate (6).[5] *tert*-Butyl (3a*R*,6a*S*)-5-oxohexahydrocyclopenta[*c*]pyrrole-2(1*H*)-carboxylate (5.0 g, 22 mmol, 1.0 eq.) was dissolved in dry DCE (100 mL). Benzylamine (2.9 mL, 26 mmol, 1.2 eq.) was added and the reaction mixture was stirred for 10 min. at RT. Acetic acid (1.0 g, 22 mmol, 1.0 eq.) was added and the reaction mixture was cooled to 0 °C. Sodium triacetoxyborohydride (2.2 g, 49 mmol, 2.2 eq.) was added and the reaction was allowed to warm to RT. After TLC analysis indicated complete conversion of starting material after 16h the mixture was concentrated *in vacuo*. The residue was taken up in EtOAc (200 mL) and the organic layer was washed with sat. $NaHCO_3$ (2 x 100 mL) and brine (1 x 100 mL). The organic layer was dried over Na_2SO_4, filtered and concentrated *in vacuo* to obtain the crude product as a green/black suspension. The title compound (**6**) was isolated via Büchi flash chromatography (DCM → 5% MeOH/DCM, 0.5% Et_3N) as a brown oil (yield: 6.23 g, 20 mmol, 88%). ^{1}H NMR (300 MHz, $CDCl_3$) δ 7.34 – 7.22 (m, 5H), 3.76 (s, 2H), 3.53 – 3.39 (m, 2H), 3.38 – 3.21 (m, 2H), 3.21 – 3.06 (m, 1H), 2.62 – 2.49 (m, 2H), 2.28 – 2.15 (m, 2H), 1.45 (s, 9H), 1.35 – 1.16 (m, 2H). ^{13}C NMR (75 MHz, $CDCl_3$) δ 154.6, 140.4, 128.4, 128.1, 126.9, 60.6, 58.5, 53.0, 52.8, 52.0, 40.8, 28.5.

Step 2: *tert*-Butyl (3a*R*,5*r*,6a*S*)-5-aminohexahydrocyclopenta[*c*]pyrrole-2(1*H*)-carboxylate (7).[5] Compound **6** (6.1 g, 19 mmol, 1.0 eq.) was dissolved in MeOH (125 mL). The reaction mixture was flushed with argon and Pd/C (10 wt %) (410 mg, 3.9 mmol, 0.2 eq.) was added. The reaction mixture was flushed with H_2 and stirred for 16h at 35 °C until TLC analysis indicated complete consumption of the starting material with formation of a more polar product. The mixture was filtered through Celite and concentrated *in vacuo* to obtain the title compound (**7**) as a yellow oil (yield: 4.4 g, 19 mmol, quant.). ^{1}H NMR (300 MHz, MeOD) δ 3.63 – 3.42 (m, 3H), 3.34 (d, J = 1.9 Hz, 2H), 2.80 – 2.64 (m, 2H), 2.40 (h, J = 13.1, 6.7 Hz, 2H), 1.45 (s, 11H). ^{13}C NMR (75 MHz, MeOD) δ 155.0, 79.6, 52.0, 51.1, 36.0, 27.4.

BB6: (3a*R*,5*r*,6a*S*)-5-Aminohexahydrocyclopenta[*c*]pyrrole-2(1*H*)-carbonitrile was synthesized according to the general procedure for cyanimide building block synthesis starting

from compound **7**. ^{1}H NMR (300 MHz, $CDCl_3$) δ 3.38 – 3.27 (m, 2H), 3.25 – 3.14 (m, 3H), 2.65 – 2.48 (m, 2H), 2.23 – 2.08 (p, 2H), 1.58 – 1.43 (s, 2H), 1.17 – 1.04 (q, 2H). ^{13}C NMR (75 MHz, $CDCl_3$) δ 117.5, 57.0, 54.6, 42.6, 41.6. HR-MS calculated for $C_8H_{13}N_3$ $[M+H]^+$ 152.1188, found 152.1207.

BB7: (3aR,5s,6aS)-5-aminohexahydrocyclopenta[c]pyrrole-2(1H)-carbonitrile.

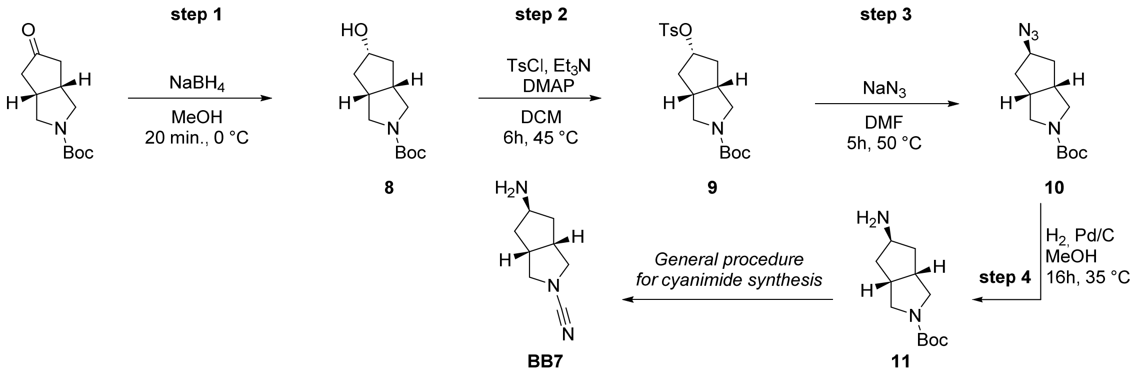

Step 1: *tert*-Butyl (3a*R*,5*r*,6a*S*)-5-hydroxyhexahydrocyclopenta[*c*]pyrrole-2(1*H*)-carboxylate (8). *tert*-Butyl (3a*R*,6a*S*)-5-oxohexahydrocyclopenta[*c*]pyrrole-2(1*H*)-carboxylate (5.0 g, 21 mmol, 1.0 eq.) was dissolved in MeOH (100 mL) and cooled to 0 °C. Sodium borohydride (1.6 g, 42 mmol, 2.0 eq.) was added and the reaction mixture was stirred at 0 °C for 20 min. until TLC analysis indicated complete consumption of the starting material. The reaction was quenched by addition of water (5 mL) and the reaction mixture was concentrated *in vacuo*. The residue was taken up in H_2O (50 mL), EtOAc (200 mL) was added and the layers were separated. The organic layer was washed with 1M HCl (1 x 50 mL) and brine (1 x 50 mL). The organic layer was dried over Na_2SO_4, filtered and concentrated *in vacuo* to obtain the title compound (**8**) as a colorless oil (yield: 4.6 g, 20 mmol, 97%). ^{1}H NMR (300 MHz, $CDCl_3$) δ 4.26 (p, *J* = 6.6, 1.1 Hz, 1H), 3.47 (dd, *J* = 11.2, 7.6 Hz, 2H), 3.31 (dd, 2H), 2.79 (s, 1H), 2.66 – 2.50 (m, 2H), 2.14 (p, 2H), 1.53 – 1.39 (m, 11H). ^{13}C NMR (75 MHz, $CDCl_3$) δ 154.7, 79.2, 74.5, 52.2, 41.0, 40.8, 28.5.

Step 2: *tert*-Butyl (3a*R*,5*r*,6a*S*)-5-(tosyloxy)hexahydrocyclopenta[*c*]pyrrole-2(1*H*)-carboxylate (9). Compound **8** (5.0 g, 21 mmol, 1.0 eq.) was dissolved in DCM (100 mL). *p*-Toluenesulfonyl chloride (4.5 g, 20 mmol, 1.2 eq.), 4-(Dimethylamino)pyridine (484 mg, 21 mmol, 0.2 eq.) and Et_3N (8.3 g, 60 mmol, 3.0 eq.) were added and the reaction mixture was stirred for 16h at 45 °C. DCM (50 mL) was added after TLC analysis indicated complete consumption of the starting material. The organic layer was washed with 1M HCl (1 x 150 mL),

sat. $NaHCO_3$ (1 x 150 mL) and brine (1 x 150 mL). The organic layer was dried over Na_2SO_4, filtered and concentrated *in vacuo* to obtain the title compound (**9**) as a brown oil (yield: 7.4 g, 19 mmol, 98%). ^{1}H NMR (300 MHz, $CDCl_3$) δ 7.74 – 7.66 (m, 2H), 7.32 – 7.23 (m, 2H), 4.83 (tt, *J* = 6.9, 5.9 Hz, 1H), 3.49 – 3.35 (m, 2H), 3.25 – 3.11 (m, 2H), 2.48 (s, 2H), 2.37 (s, 3H), 2.06 (s, 2H), 1.60 (m, 2H), 1.39 (s, 9H). ^{13}C NMR (75 MHz, $CDCl_3$) δ 154.4, 144.7, 134.0, 129.9, 127.7, 83.7, 79.3, 51.3, 37.8, 29.7, 28.5, 21.6.

Step 3: *tert*-Butyl (3a*R*,5*s*,6a*S*)-5-azidohexahydrocyclopenta[*c*]pyrrole-2(1*H*)-carboxylate (10). Compound **9** (7.4 g, 19 mmol, 1.0 eq.) was dissolved in DMF (100 mL). Sodium azide (6.3 g, 97 mmol, 5 eq.) was added and the reaction mixture was stirred for 5h at 50 °C. After TLC analysis indicated complete consumption of the starting material the mixture was concentrated *in vacuo*. The residue was taken up in EtOAc (200 mL) and the organic layer was washed with H_2O (2 x 200 mL) and 1M LiCl (1 x 200 mL). The organic layer was dried over Na_2SO_4, filtered and concentrated *in vacuo* to obtain the crude product as a brown suspension. The title compound was isolated via Büchi flash chromatography (Heptane -> 20% EtOAc/Heptane) to obtain the title compound (**10**) as a colorless oil (yield: 4.7 g, 18 mmol, 95%). ^{1}H NMR (300 MHz, $CDCl_3$) δ 4.07 – 4.01 (m, 1H), 3.48 – 3.35 (m, 2H), 3.21 – 3.06 (m, 2H), 2.83 – 2.63 (m, 2H), 1.96 – 1.85 (m, 2H), 1.72 – 1.58 (m, 2H), 1.38 (s, 9H). ^{13}C NMR (75 MHz, $CDCl_3$) δ 154.6, 79.3, 63.4, 51.7, 38.3, 37.6, 29.7, 28.5.

Step 4: *tert*-Butyl (3a*R*,5*s*,6a*S*)-5-aminohexahydrocyclopenta[*c*]pyrrole-2(1*H*)-carboxylate (11). Compound **10** (4.7 g, 18 mmol, 1.0 eq.) was dissolved in MeOH (220 mL). The reaction mixture was flushed with argon and Pd/C (10 wt%) (410 mg, 3.9 mmol, 0.1 eq.) was added. The reaction was flushed with H_2 and stirred for 16h at 35 °C until TLC analysis indicated complete consumption of the starting material with formation of a more polar product. The mixture was filtered through Celite and concentrated *in vacuo* to obtain the title compound (**11**) as a white solid (yield: 4.2 g, 18 mmol, quant.). ^{1}H NMR (300 MHz, $CDCl_3$) δ 3.56 – 3.37 (m, 3H), 3.05 (t, J = 6.3 Hz, 2H), 2.83 – 2.67 (m, 3H), 2.23 – 2.07 (m, 1H), 1.85 (dt, J = 14.6, 5.9 Hz, 1H), 1.70 (dp, J = 13.4, 3.3 Hz, 1H), 1.57 (ddd, J = 13.2, 8.1, 6.4 Hz, 1H), 1.38 (s, 9H). ^{13}C NMR (75 MHz, $CDCl_3$) δ 154.5, 79.1, 52.5, 43.3, 41.8, 28.5.

BB7: (3a*R*,5*s*,6a*S*)-5-Aminohexahydrocyclopenta[*c*]pyrrole-2(1*H*)-carbonitrile was synthesized according to the general procedure for cyanimide building block synthesis starting from compound **11**.[5] ^{1}H NMR (300 MHz, $CDCl_3$) δ 3.50 (p, *J* = 6.0 Hz, 1H), 3.45 – 3.36 (m, 2H), 3.05 (dd, *J* = 9.8, 3.4 Hz, 2H), 2.85 – 2.75 (m, 2H), 1.70 – 1.52 (m, 4H), 1.38 – 1.32 (m,

2H). ^{13}C NMR (75 MHz, $CDCl_3$) δ 117.51, 56.99, 54.53, 42.57, 41.60. HR-MS calculated for $C_8H_{13}N_3$ $[M+H]^+$ 152.1188, found 152.1185.

BB8: (*S*)-3-Aminopiperidine-1-carbonitrile was synthesized according to the general procedure for cyanimide building block synthesis starting from (*S*)-*tert*-butyl 3-aminopiperidine-1-carboxylate. ^1H NMR (300 MHz, $CDCl_3$) δ 3.23 (dd, *J* = 29.1, 14.5 Hz, 2H), 2.97 – 2.78 (m, 2H), 2.61 (t, 1H), 1.91 – 1.77 (m, 1H), 1.75 – 1.35 (m, 4H), 1.21 – 1.04 (m, 1H). ^{13}C NMR (75 MHz, $CDCl_3$) δ 118.2, 56.8, 49.3, 46.5, 32.6, 22.8. HR-MS calculated for $C_6H_{11}N_3$ $[M+H]^+$ 126.1031, found 126.1034.

BB9: (*R*)-4-(aminomethyl)-*N*-(1-cyanopyrrolidin-3-yl)benzamide.

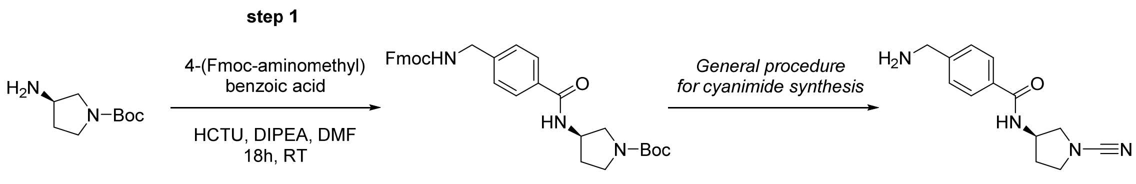

12 BB9

***tert*-Butyl (*R*)-3-(4-(((((9*H*-fluoren-9-yl)methoxy)carbonyl)amino)methyl)benzamido) pyrrolidine-1-carboxylate (12).** 4-(Fmoc-aminomethyl)benzoic acid (3.0 g, 8.05 mmol, 1.5 eq.) was dissolved in DMF (30 mL) and HCTU (3.33 g, 8.05 mmol, 1.5 eq.) and DiPEA (2.8 mL, 16.11 mmol, 3.0 eq.) were added. The resulting reaction mixture was stirred at rt for 10 min. (*R*)-(+)-1-Boc-3-aminopyrrolidine (1.0 g, 5.37 mmol, 1.0 eq) was added and the resulting reaction mixture was stirred at rt for 18h. The reaction mixture was concentrated *in vacuo* and the crude residue was taken up in EtOAc (100 mL). The organic layer was washed with 1M HCl (2 x 100 mL), sat. $NaHCO_3$ (2 x 100 mL) and brine (1 x 50 mL). The organic layer was dried over Na_2SO_4, filtered and concentrated *in vacuo*. The product was isolated *via* Büchi flash chromatography (Heptane → 70% EtOAc/Heptane) to yield compound **12** as a white solid (2.34 g, 4.32 mmol, 80%).

BB9: (*R*)-4-(aminomethyl)-*N*-(1-cyanopyrrolidin-3-yl)benzamide was synthesized according to the general procedure for cyanimide building block synthesis starting from compound **12**. ^1H NMR (300 MHz, MeOD) δ 7.90 – 7.78 (m, 2H), 7.51 – 7.42 (m, 2H), 4.68 – 4.52 (m, 1H), 3.87 (s, 2H), 3.75 (dd, *J* = 9.9, 6.4 Hz, 1H), 3.70 – 3.49 (m, 2H), 3.46 – 3.37 (ddd, *J* = 9.9, 4.7, 0.7 Hz, 1H), 2.36 – 2.22 (m, 1H), 2.15 – 2.02 (m, 1H). ^{13}C NMR (75 MHz,

MeOD) δ 168.9, 146.1, 132.4, 127.4, 127.1, 116.9, 54.7, 50.1, 48.7, 44.8, 30.6. HR-MS calculated for $C_{13}H_{16}N_4O$ $[M+H]^+$ 245.1402, found 245.1409.

BB10: (*S*)-1-Cyano-*N*-(4,5,6,7-tetrahydrothiazolo[5,4-*c*]pyridin-2-yl)pyrrolidine-3-carboxamide was synthesized according to the general procedure for cyanimide building block synthesis starting from (9*H*-fluoren-9-yl)methyl (*S*)-2-(1-(*tert*-butoxycarbonyl)pyrrolidine-3-carboxamido)-6,7-
dihydrothiazolo [5,4-*c*]pyridine-5(4*H*)-carboxylate. ^1H NMR (300 MHz, $CDCl_3$) δ 4.00 (s, 2H), 3.74 – 3.57 (m, 3H), 3.47 (dt, *J* = 9.3, 7.4 Hz, 1H), 3.32 – 3.12 (m, 3H), 2.74 – 2.60 (m, 2H), 2.22 (td, *J* = 7.4, 5.2 Hz, 2H). ^{13}C NMR (75 MHz, $CDCl_3$) δ 169.33, 155.97, 142.92, 122.53, 116.90, 52.79, 50.38, 44.29, 43.63, 43.26, 29.75, 27.77. HR-MS calculated for $C_{12}H_{15}N_5OS$ $[M+H]^+$ 278.1075, found 278.1086.

BB11: (*R*)-3-(Aminomethyl)pyrrolidine-1-carbonitrile was synthesized according to the general procedure for cyanimide building block synthesis starting from (*R*)-1-Boc-3-(aminomethyl)pyrrolidine. ^1H NMR (300 MHz, $CDCl_3$) δ 3.56 – 3.31 (m, 3H), 3.13 (t, 1H), 2.80 – 2.63 (m, 2H), 2.27 (hept, *J* = 7.0 Hz, 1H), 2.11 – 1.94 (m, 1H), 1.75 – 1.55 (m, 1H), 1.26 (s, 2H). ^{13}C NMR (75 MHz, $CDCl_3$) δ 117.7, 53.8, 50.1, 44.3, 42.3, 29.3. HR-MS calculated for $C_6H_{11}N_3$ $[M+H]^+$ 126.1031, found 126.1036.

BB12: (1*S*,4*S*)-2,5-Diazabicyclo[2.2.1]heptane-2-carbonitrile was synthesized according to the general procedure for cyanimide building block synthesis starting from *tert*-butyl (1*S*,4*S*)-2,5-diazabicyclo[2.2.1]heptane-2-carboxylate. ^1H NMR (300 MHz, $CDCl_3$) δ 4.04 (s, 1H), 3.67 (s, 1H), 3.45 (d, *J* = 8.8 Hz, 1H), 3.20 (dd, *J* = 15.8, 9.7 Hz, 2H), 2.96 (d, *J* = 10.5 Hz, 1H), 1.90 – 1.65 (m, 3H). ^{13}C NMR (75 MHz, $CDCl_3$) δ 117.4, 62.1, 61.1, 55.8, 51.2, 36.9. HR-MS calculated for $C_6H_9N_3$ $[M+H]^+$ 124.0875, found 124.0871.

BB13: (3*S*,4*S*)-3-Amino-4-hydroxypyrrolidine-1-carbonitrile was synthesized according to the general procedure for cyanimide building block synthesis starting from (3*S*,4*S*)-N-Boc-3-amino-4-hydroxypyrrolidine. ^1H NMR (300 MHz, MeOD) δ 4.77 (s, 2H), 4.06 – 3.99 (m, 1H), 3.74 (m, 2H), 3.41 – 3.18 (m, 4H). ^{13}C NMR (75 MHz, MeOD) δ 117.4, 75,7, 57.4, 55.7, 55.6. HR-MS calculated for $C_5H_9N_3O$ $[M+H]^+$ 128.0824, found 128.0831.

BB14: 2,7-Diazaspiro[4.4]nonane-2-carbonitrile was synthesized according to the general procedure for cyanimide building block synthesis starting from tert-butyl 2,7-diazaspiro[4.4]nonane-2-carboxylate. ^1H NMR (300 MHz, $CDCl_3$) δ 3.60 – 3.41 (m, 3H), 3.38

– 3.25 (m, 2H), 3.05 (t, *J* = 7.1 Hz, 2H), 2.95 – 2.79 (m, 2H), 1.94 – 1.86 (m, 2H), 1.85 – 1.67 (m, 2H). ^{13}C NMR (75 MHz, $CDCl_3$) δ 117.5, 60.2, 56.3, 50.0, 46.2, 36.4, 36.2. HR-MS calculated for $C_8H_{13}N_3$ $[M+H]^+$ 152.1188, found 152.1182.

BB15: 4-(4-Amino-1H-pyrazol-1-yl)piperidine-1-carbonitrile was synthesized according to the general procedure for cyanimide building block synthesis starting from *cis*-1-Boc-3-amino-4-fluoropyrrolidine. ^{1}H NMR (300 MHz, $CDCl_3$) δ 4.99 – 4.94 (m, 0.5H), 4.79 (t, *J* = 2.8 Hz, 0.5H), 3.78 (d, *J* = 2.0 Hz, 1H), 3.73 – 3.64 (m, 2H), 3.65 – 3.45 (m, 1H), 3.25 – 3.16 (m, 1H). ^{13}C NMR (75 MHz, $CDCl_3$) δ 116.45, 92.47 (d, *J* = 181.3 Hz), 55.36 (d, *J* = 23.1 Hz), 54.86 (d, *J* = 19.2 Hz), 53.77 (d, *J* = 1.8 Hz). HR-MS calculated for $C_5H_8FN_3$ $[M+H]^+$ 130.0780, found 130.0802.

BB16: 4-(4-Amino-1*H*-pyrazol-1-yl)piperidine-1-carbonitrile was synthesized according to the general procedure for cyanimide building block synthesis starting from 4-amino-1-(1-Boc-piperidin-4-yl)-1*H*-pyrazole. ^{1}H NMR (300 MHz, $CDCl_3$) δ 7.18 (d, *J* = 0.9 Hz, 1H), 7.04 (d, *J* = 0.8 Hz, 1H), 4.12 (tt, *J* = 10.2, 5.1 Hz, 1H), 3.67 – 3.50 (m, 2H), 3.27 – 3.11 (m, 2H), 2.93 (s, 2H), 2.20 – 2.07 (m, 4H). HR-MS calculated for $C_9H_{13}N_5$ $[M+H]^+$ 192.1249, found 192.1253. ^{13}C NMR (75 MHz, $CDCl_3$) δ 131.2, 129.2, 117.8, 115.9, 57.3, 48.9, 31.3.

BB07CA28 and BB07CA48

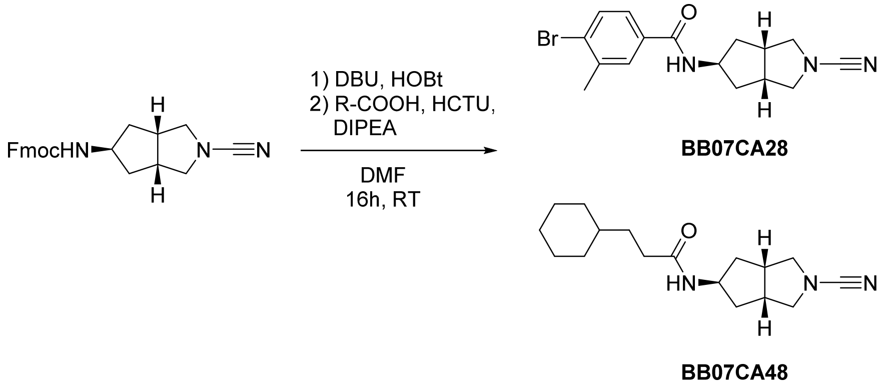

BB07CA28: 4-Bromo-*N*-((3a*R*,5*s*,6a*S*)-2-cyanooctahydrocyclopenta[*c*]pyrrol-5-yl)-3-methylbenzamide. Fmoc-protected **BB07** (50 mg, 133 µmol, 1.0 eq.) was dissolved in DMF (1 mL). DBU (0.5 eq., 57 µmol, 10 µL) was added and the mixture was stirred for 30 min. until TLC indicated complete removal of the Fmoc group. The reaction was quenched by addition of HOBt (1.5 eq., 201 µmol, 27 mg) and stirred for another 30 min. 4-Bromo-3-methylbenzoic acid (1.5 eq., 201 µmol, 31 mg) was dissolved in DMF (1 mL) and to this were added DiPEA

(3.0 eq., 0.40 mmol, 70 µL) and HCTU (1.5 eq., 201 µmol, 10 mg). To reaction was stirred for 5 min. before being added to the deprotected amine. The whole reaction mixture was stirred for 16h after which it was concentrated under reduced pressure. The residue was taken up in EtOAc (20 mL) and washed with 1M HCl (2 x 10 mL), sat. $NaHCO_3$ (2 x 10 mL) and brine (1 x 10 mL). The organic layer was dried over Na_2SO_4, filtered and concentrated *in vacuo*. The product was purified by Büchi flash chromatography (Heptane → EtOAc) to obtain the title compound as a colorless solid (yield: 33 mg, 113 µmol, 85%). ^{1}H NMR (300 MHz, $CDCl_3$) δ 7.56 – 7.52 (d, *J* = 2.2 Hz, 1H), 7.52 – 7.45 (d, *J* = 8.2 Hz, 1H), 7.33 – 7.26 (dd, *J* = 8.2, 2.3 Hz, 1H), 6.09 (d, *J* = 7.1 Hz, 1H), 4.45 (h, *J* = 7.1 Hz, 1H), 3.54 – 3.38 (m, 2H), 3.10 (dd, *J* = 10.0, 3.9 Hz, 2H), 2.81 (dq, *J* = 7.7, 3.9 Hz, 2H), 2.35 (s, 3H), 1.97 – 1.86 (ddd, *J* = 13.4, 6.5, 3.3 Hz, 2H), 1.84 – 1.72 (dt, *J* = 13.5, 8.0 Hz, 2H). ^{13}C NMR (75 MHz, $CDCl_3$) δ 166.6, 138.4, 133.5, 132.5, 129.4, 128.5, 125.5 116.9, 57.3, 51.1, 41.2, 38.6, 22.9. HR-MS calculated for $C_{16}H_{18}BrN_3O$ $[M+H]^+$ 350.0711, found 350.0757.

BB07CA48: *N*-((3a*R*,5*s*,6a*S*)-2-cyanooctahydrocyclopenta[*c*]pyrrol-5-yl)-3-cyclohexylpropanamide. This compound was synthesized according to the procedure described for **BB07CA28**, but coupling 3-cyclohexylpropanoic acid on a the same scale. (yield: 31 mg, 106 µmol, 80%). ^{1}H NMR (300 MHz, $CDCl_3$) δ 5.42 (d, *J* = 7.2 Hz, 1H), 4.29 (h, *J* = 7.1 Hz, 1H), 3.46 (dd, *J* = 10.0, 7.9 Hz, 2H), 3.07 (dd, *J* = 10.0, 3.9 Hz, 2H), 2.82 – 2.65 (m, 2H), 2.14 – 2.02 (m, 2H), 1.90 – 1.76 (m, 2H), 1.75 – 1.50 (m, 7H), 1.49 – 1.37 (m, 2H), 1.27 – 1.01 (m, 4H), 0.91 – 0.72 (m, 2H). ^{13}C NMR (75 MHz, $CDCl_3$) δ 173.2, 116.9, 57.3, 50.4, 41.2, 38.6, 37.4, 34.3, 33.1, 26.5, 26.2. HR-MS calculated for $C_{17}H_{27}N_3O$ $[M+H]^+$ 290.2232, found 290.2268.

BB07CA902 and BB06CA902

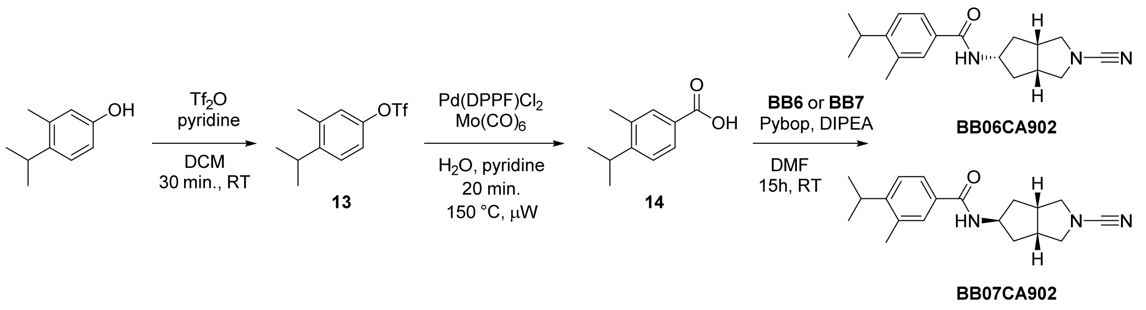

4-Isopropyl-3-methylphenyl trifluoromethanesulfonate (13). A solution of 4-isopropyl-3-methylphenol (1.5 g, 10 mmol, 1.0 eq.) in dry DCM (50 mL) was flushed with argon and cooled to 0 °C. Dry pyridine (2.0 eq., 20 mmol, 1.6 mL) was added followed by a dropwise addition of

trifluoromethanesulfonic anhydride (1.2 eq., 12 mmol, 2.1 mL) The reaction was slowly warmed to room temperature and TLC analysis indicated complete conversion of starting material after 30 min. The reaction was quenched by addition of water (10 mL), followed by 1M HCl (10 mL). The mixture was diluted with Et_2O (100 mL), the layers were separated and the organic layer was washed with 1M HCl (1 x 50 mL), sat. $NaHCO_3$ (1 x 50 mL) and brine (1 x 50 mL). The organic layer was dried over Na_2SO_4, filtered and concentrated *in vacuo* to obtain the title compound as a colorless oil (yield: 2.79 g, 9.89 mmol, 99%). ^{1}H NMR (300 MHz, $CDCl_3$) δ 7.28 (d, *J* = 8.6 Hz, 1H), 7.06 (dd, *J* = 8.5, 2.7 Hz, 1H), 7.02 (d, *J* = 2.7 Hz, 1H), 3.13 (hept, *J* = 6.8 Hz, 1H), 2.36 (s, 3H), 1.22 (d, *J* = 6.9 Hz, 6H). ^{13}C NMR (75 MHz, $CDCl_3$) δ 147.30, 137.87, 126.61, 122.60, 121.03, 118.81, 29.19, 23.23, 19.52.

4-Isopropyl-3-methylbenzoic acid (14). This compound was synthesized using a reported procedure.[6] A microwave tube was charged with a stirring bar, triflate **13** (1.13 g, 4.0 mmol, 1 eq.), Pd(DPPF)Cl_2 (0.1 eq., 0.4 mmol, 293 mg), water (12 mL), pyridine (1.5 mL) and $Mo(CO)_6$ (0.5 eq., 2 mmol, 528 mg). The tube was capped and the mixture was heated to 150 °C in a microwave for 20 min. while stirring. After cooling the black reaction mixture the cap was removed and 6M aqueous HCl (10 mL) was added. The mixture was extracted with Et_2O (2 x 50 mL) after which the combined organic layers were extracted with 2M NaOH (2 x 20 mL). The combined NaOH layers were acidified with concentrated HCl followed by extraction with EtOAc (2 x 50 mL). The combined EtOAc layers were dried over Na_2SO_4, filtered and concentrated *in vacuo* to obtain the title compound as a colorless solid (yield: 180 mg, 1.0 mmol, 25%). ^{1}H NMR (300 MHz, $CDCl_3$) δ 10.40 (s, 1H), 7.93 (d, *J* = 8.1 Hz, 1H), 7.90 (s, 1H), 7.34 (d, *J* = 8.1 Hz, 1H), 3.20 (hept, *J* = 6.9 Hz, 1H), 2.40 (s, 3H), 1.25 (d, *J* = 6.9 Hz, 6H). ^{13}C NMR (75 MHz, $CDCl_3$) δ 172.54, 153.54, 135.52, 132.04, 128.36, 126.62, 125.09, 29.74, 23.05, 19.39.

BB07CA902: *N*-((3a*R*,5*s*,6a*S*)-2-cyanooctahydrocyclopenta[*c*]pyrrol-5-yl)-4-isopropyl-3-methylbenzamide. Fmoc-protected **BB07** (14 mg, 37 µmol, 1.0 eq.) was dissolved in DMF (0.5 mL). DBU (0.5 eq., 18.5 µmol, 2.76 µL) was added and the mixture was stirred for 30 min. until TLC indicated complete removal of the Fmoc group. The reaction was quenched by addition of HOBt (1.5 eq., 55 µmol, 7.5 mg) and stirred for another 30 min. Carboxylic acid **14** (1.5 eq., 56 µmol, 10 mg) was dissolved in DCM (0.5 mL) and to this were added DiPEA (3.0 eq., 0.11 mmol, 20 µL) and Pybop (1.5 eq., 56 µmol, 29 mg). To reaction was stirred for 5 min. before being added to the deprotected amine. The whole reaction mixture was stirred for 15h after which it was concentrated under reduced pressure. The residue was taken up in EtOAc (20 mL) and washed with sat. $NaHCO_3$ (2 x 10 mL) and brine (1 x 10 mL). The organic layer was dried over Na_2SO_4, filtered and concentrated *in vacuo*. The product was purified by Büchi

flash chromatography (Heptane → EtOAc) to obtain the title compound as a colorless solid (yield: 10 mg, 32 μmol, 87%). ^{1}H NMR (300 MHz, $CDCl_3$) δ 7.54 – 7.47 (m, 2H), 7.28 (d, *J* = 4.5 Hz, 1H), 5.97 (d, *J* = 7.1 Hz, 1H), 4.55 (h, *J* = 7.1 Hz, 1H), 3.55 (dd, *J* = 10.0, 7.9 Hz, 2H), 3.24 – 3.09 (m, 3H), 2.86 (dt, *J* = 7.7, 4.0 Hz, 2H), 2.37 (s, 3H), 1.99 (ddd, *J* = 13.4, 6.4, 3.4 Hz, 2H), 1.90 – 1.78 (m, 2H), 1.22 (d, *J* = 6.9 Hz, 6H). ^{13}C NMR (75 MHz, $CDCl_3$) δ 167.54, 151.03, 135.67, 131.68, 128.89, 125.11, 124.68, 117.05, 57.42, 51.14, 41.42, 38.82, 29.50, 23.13, 19.46. HR-MS calculated for $C_{19}H_{25}N_3O$ $[M+H]^+$ 312.2076, found 312.2100.

BB06CA902: *N*-((3a*R*,5*r*,6a*S*)-2-cyanooctahydrocyclopenta[*c*]pyrrol-5-yl)-4-isopropyl-3-methylbenzamide. This compound was synthesized according to the procedure described for **BB07CA902**, starting from **BB06** on a 25 mg, 67 μmol scale. (yield: 16 mg, 51 μmol, 76%). ^{1}H NMR (300 MHz, $CDCl_3$) δ 7.54 (m, 2H), 7.29 (d, *J* = 8.1 Hz, 1H), 6.22 (d, *J* = 5.7 Hz, 1H), 4.50 – 4.32 (m, 1H), 3.42 (dd, *J* = 10.0, 7.0 Hz, 2H), 3.31 (dd, *J* = 10.0, 1.8 Hz, 2H), 3.16 (hept, *J* = 6.9 Hz, 1H), 2.73 (dd, *J* = 7.9, 4.7 Hz, 2H), 2.46 (dt, *J* = 13.8, 7.1 Hz, 2H), 2.38 (s, 3H), 1.38 (m, 2H), 1.22 (d, *J* = 6.8 Hz, 6H). ^{13}C NMR (75 MHz, $CDCl_3$) δ 151.07, 135.72, 128.82, 125.19, 124.75, 57.47, 51.57, 41.20, 39.69, 29.52, 23.14, 19.47. HR-MS calculated for $C_{19}H_{25}N_3O$ $[M+H]^+$ 312.2076, found 312.2100.

Echo synthesis and analysis

Test reaction and optimizations

The coupling of carboxylic acid **CA64** with amine **BB1** was used as a model reaction to find the optimal conditions for in-plate synthesis. Reactions were conducted in 1.5 mL Eppendorf tubes using DMSO as solvent at a total volume of 300 μL. Stock solutions of **BB1** (200 mM in DMSO), **CA64** and all reagents (each 500 mM in DMSO) were made. Empty vials were first charged with DMSO, followed by addition of **CA64**, coupling reagents and base where appropriate. All tested reaction conditions are listed in the table below. After 30 min. **BB1** was added and samples were taken after 1h, 3h and 16h, and analyzed by TLC (15% MeOH/DCM). The choice for the optimal reaction conditions (condition 6.2: 5 eq. DIC/HOBt) was based on the degree of consumed **BB1** and the amount of undesired side product formation.

Condition	Amine:Reagent:base (eq.)	Coupling reagent/Base
1.1	1:2:3	HBTU/NMM
1.2	1:5:15	HBTU/NMM

1.3	1:10:30	HBTU/NMM
2.1	1:2:3	HCTU/NMM
2.2	1:5:15	HCTU/NMM
2.3	1:10:30	HCTU/NMM
3.1	1:2:3	HATU/NMM
3.2	1:5:15	HATU/NMM
3.3	1:10:30	HATU/NMM
4.1	1:2:3	Pybop/NMM
4.2	1:5:15	Pybop/NMM
4.3	1:10:30	Pybop/NMM
5.1	1:2	EDC+HOBt
5.2	1:5	EDC+HOBt
5.3	1:10	EDC+HOBt
6.1	1:2	DIC+HOBt
6.2	1:5	DIC+HOBt
6.3	1:10	DIC+HOBt
7.1	1:2:3	EDC/DMAP
7.2	1:5:15	EDC/DMAP
7.3	1:10:30	EDC/DMAP
8.1	1:2:3	DIC/DMAP
8.2	1:5:15	DIC/DMAP
8.3	1:10:30	DIC/DMAP

NMM: *N*-methylmorpholine; HBTU: 2-(1*H*-benzotriazol-1-yl)-1,1,3,3-tetramethyluronium hexafluorophosphate; HCTU: *O*-(1*H*-6-Chlorobenzotriazole-1-yl)-1,1,3,3-tetramethyluronium hexafluorophosphate ; HATU: 1-[Bis(dimethylamino)methylene]-1*H*-1,2,3-triazolo[4,5-b]pyridinium 3-oxide hexafluorophosphate; Pybop: (Benzotriazol-1-yloxy)tripyrrolidinophosphonium hexafluorophosphate; DIC: *N*,*N*'-diisopropylcarbodiimide, EDC: 1-Ethyl-3-(3-dimethylaminopropyl)carbodiimide; HOBt: 1-hydroxybenzotriazool; DMAP: 4-dimethylaminopyridine. No base was used for conditions where HOBt was used.

Carboxylic acid selection

Carboxylic acids were chosen from all in-house available acids (89 compounds) and purchased from Enamine (382 compounds). For the acids obtained from Enamine, the following procedure was followed: A list containing all available bicyclic carboxylic acids with molecular weight below 400 Da was obtained. All compounds containing any of the moieties

shown below were excluded. This resulted in a list of 1,304 compounds. The final selection of 382 compounds was made based on a high degree of structural variety, pricing and immediate availability. The compounds were received as 100 mM solutions in DMSO in 96-deepwell plates. Compounds were transferred to 384PP Echo-ready plates (Labcyte P-05525) using a multichannel pipette and stored at -20 °C.

Excluded moieties:

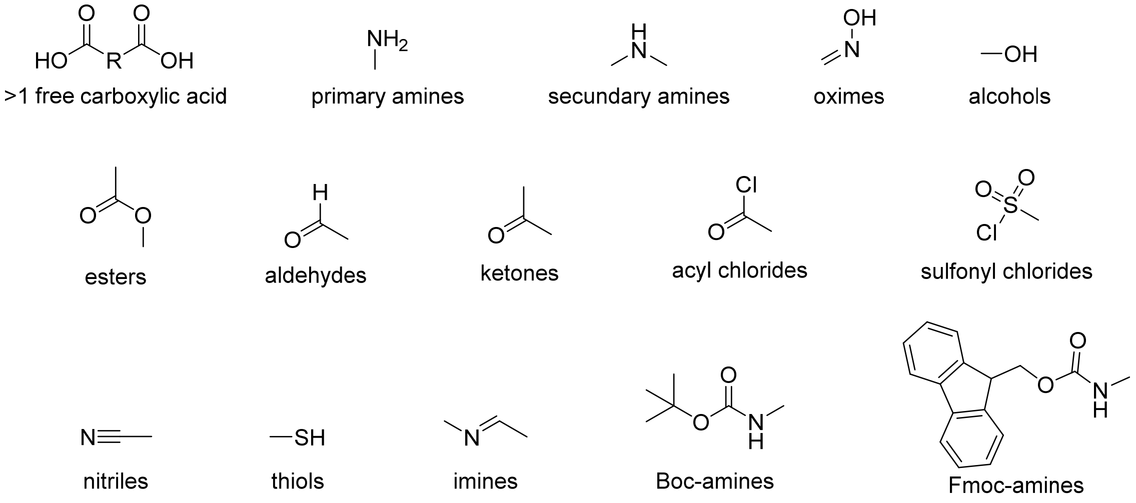

Miniaturized high-throughput Echo synthesis

Synthesis in 384 well plates. Synthesis was performed in 384LDV Echo-ready plates (Labcyte LP-0200). Each well had a total volume of 10 µL. First, 6 µL DMSO was dispensed in each well using a CombiNL Microplate dispenser (ThermoFisher). Next, 2.5 µL of the carboxylic acids **CA1-CA89** (200 mM in DMSO), 0.5 µL DIC (1M DMSO) and 0.5 µL HOBt (1M DMSO) were transferred using an Echo550 acoustic dispenser. After 30 min. incubation, 0.5 µL of the amines **BB1-BB4** and **BB6-BB8** (200 mM in DMSO) were transferred using an Echo550 acoustic dispenser. The plates were sealed and kept for 16h at room temperature and afterwards stored at -20 °C.

Synthesis in 1536 well plates (plates NCN1-5)

Synthesis was performed in 1536LDV Echo-ready plates (Labcyte LP-0400). Each well had a total volume of 2.5 µL. First, each well was charged with 1125 nL of a solution of DIC + HOBt (111.1 mM) in DMSO using a CombiNL Microplate dispenser (ThermoFisher). Next, 1250 nL of the carboxylic acids **CA1-CA469** (100 mM in DMSO) were transferred using an Echo550

acoustic dispenser. After 30 min. incubation, 125 nL of the amines **BB1-BB16** (200 mM in DMSO) were transferred using an Echo550 acoustic dispenser. The synthesis plates were sealed and kept for 16h at room temperature, after which 10 fold dilution daughter plates (1 mM compound concentration) were made, by dispensing 2700 nL DMSO into empty 1536LDV Echo-ready plates (using a CombiNL Microplate dispenser) followed by transfer of 300 nL from the synthesis plates (using an Echo550 acoustic dispenser). All plates were sealed and stored at -20 °C. In each plate, at least 8 wells were left empty for future controls. Plate lay-outs can be found in the supporting data files.

Mass spectrometry analysis of library compounds

Compounds (30 nL) from the indicated wells in the synthesis plates (see Figures) were transferred to a 96 well LC-MS plate using the Echo550 acoustic dispenser, diluted with 30 µL acetonitrile/water 1:1 v/v and analyzed on a Waters Xevo LC-MS system by injecting 1 µL sample. For the indicated 1,536 well plates, 48 compounds diagonally distributed (‘hockey stick’ or ‘checkmark’ pattern) over the plate were analyzed. By picking the compounds in such a way, at least one compound from each row and each column is analyzed, and as such serves as a good representation of the whole plate, without the need to check all 1,536 compounds in each plate. All MS spectra were analyzed and checked for the presence of the product, CA, BB and possible side products. Based on this, each compound well was annotated as ‘good’ (>90% conversion, almost no side products), ‘medium’ (50-90% conversion, or mass difficult to find due to bad ionization, some side products), or ‘bad’ (no to low conversion, many side products), and colored accordingly: green, orange and red, respectively. In some cases the ‘hockey stick’ pattern resulted in an empty well taken along in the analysis (for example N24, P24, N48, P48). These wells were purposely left empty to use as control wells in the high-throughput screen.

Biochemistry methods

Purified recombinant enzymes used in this study

Enzyme	Domain	Tag	UniProt ID	Source	Final assay concentration
UCH-L1	Full length (1-223)	-	P09936	*In-house* [Larsen, 1996][7]	1 nM
UCH-L3	Full length (1-230)	-	P15374	*In-house*, [Larsen, 1996][7]	0.01 nM
UCH-L5	Full length (1-329)	-	Q9Y5K5	Novus biochemicals #NBP1-72315	1 nM
OTUD1	CD (287-481)	His6 tag	Q5VV17	*In-house,* [Mevissen, 2013][16]	5 nM
OTUB2	Full length (1-234)	His6, cleaved	Q96DC9	*In-house*, [Nanao, 2004][8]	25 nM
USP7	Full length (1-1102)	GST-tagged, cleaved	Q93009	*In-house*, [Kim, 2019][9]	1 nM
USP8	Full length (1-1118)	-	P40818	Ubiquigent #64-0053-050	5 nM
USP16	Full length (22-823)	N-terminal His-tag	Q9Y5T5	*In-house*, [Mons, 2021][10]	2 nM
mUSP18	Full length (46-368)	N-terminal His-tag	Q9WTV6	[Basters, 2014][11]	2 nM
hUSP18	Full length (16-372)	His6-GST tag	Q9UMW8	*In-house,* [Basters, 2017][1]	2 nM
USP24	Full length (1-2620)	His10 tag	Q9UPU5	Biotechne E-616	0.4 nM
USP30	CD (57-517)	-	Q70CQ3	Ubiquigent #64-0057-050	10 nM
USP32	Full length (1-1604)	N-terminal His-tag	Q8NFA0	*In-house*, [Sapmaz, 2019][12]	0.8 nM
SENP1	CD (419-644)	His6, cleaved	Q9P0U3	*In-house,* [Huppelschoten, 2023][17]	1 nM
Papain (from papaya latex)		-	P00784	Sigma Aldrich #P3125	3 nM

IC_{50} and k_{inact}/K_I determination

The assays were performed at room temperature in "nonbinding surface flat bottom low flange" black 384-well plates (Corning 3820) at room temperature in a buffer containing 50 mM Tris·HCl, 100 mM NaCl, pH 7.6, 2.0 mM cysteine, 1 mg/mL 3-[(3-cholamidopropyl)-dimethylammonio]propanesulfonic acid (CHAPS), and 0.5 mg/mL γ-globulins from bovine blood (BGG). Each well had a final volume of 20.4 µL. The compounds were dissolved in 10 mM DMSO stocks, and appropriate volumes were transferred to the empty plates using a Labcyte Echo550 acoustic dispenser. A DMSO back-fill was performed to obtain equal volumes of DMSO (400 µL) in each well. *N*-ethylmaleimide (NEM, 10 mM) was used a positive control (100% inhibition) and DMSO as negative control (0% inhibition). All buffer dispensing steps were performed on a Biotek MultiflowFX liquid dispenser. A 10 µL portion of buffer was added and the plate was vigorously shaken for 20s. Next, 5 µL of recombinant enzyme (4x final concentration) was added followed by incubation for 1h. A 5 µL portion of the substrate (Ub-RhoMP[2] for all DUBs, ISG15ct-RhoMP (*vide supra*) for USP18 and Z-FR-AMC [Bachem, #I-1160] for papain) was added (final concentration 400 nM for Ub-RhoMP and ISG15ct-RhoMP, 10 µM for Z-FR-AMC), and the increase in fluorescence over time was recorded using a BMG Labtech PHERAstar plate reader (excitation 487 nm, emission 535 nm for Rho; excitation 360 nm, emission 450 nm for AMC). The initial enzyme velocities were calculated from the slopes, normalized to the positive and negative controls, and plotted against the inhibitor concentrations using the built-in equation "[inhibitor] vs response − variable slope (four parameters), least-squares fit" with constraints "Bottom = 0" and "Top = 100" in GraphPad Prism 7 software to obtain the IC_{50} values.

In case of the k_{inact}/K_I determinations the order of mUSP18 and substrate addition was reversed and no incubation time was used. All data fitting and calculations were done using GraphPad Prism 7 software. The fluorescence intensities were plotted against time (in seconds) after a baseline correction using the DMSO control for each inhibitor concentration. The data were fitted to the equation:

$$FI = \frac{v_i}{k_{obs}}(1 - e^{-k_{obs}t})$$

Where FI is the fluorescence intensity (in AU), v_i is the initial enzyme velocity (in AU/sec), k_{obs} is the observed rate constant (in sec^{-1}) and t is the time (in sec). Thus obtained k_{obs} values were plotted against the inhibitor concentration ([inh]) and fitted using linear regression. The slope of the plot was determined, which is equal to the k_{inact}/K_I value.

High-throughput screening

The screens were performed at room temperature in “flat bottom, non-treated, black polystyrene” 1536-well plates (Corning 3724) at room temperature in a buffer containing 50 mM Tris·HCl, 100 mM NaCl, pH 7.6, 2.0 mM cysteine, 1 mg/mL 3-[(3-cholamidopropyl)-dimethylammonio]propanesulfonic acid (CHAPS), and 0.5 mg/mL γ-globulins from bovine blood (BGG). Each well had a final volume of 8 μL and final compound concentration was 1.25 μM. The compounds (10 nL each) were transferred from the 1 mM daughter plates to the empty assay plates using a Labcyte Echo550 acoustic dispenser. Four wells containing DMSO (10 nL) and four wells containing NEM (10 mM final concentration) were used in each plate as negative (0% inhibition) and positive (100% inhibition) controls, respectively. All buffer dispensing steps were performed on a Biotek MultiflowFX liquid dispenser. A 6 μL enzyme solution (1.33x final concentration) was added to each well, followed by incubation for 30 min. Next, 2 μL of a substrate solution (4x final concentration) was added to each well (Ub-RhoMP[2] for all DUBs and ISG15ct-RhoMP (*vide supra*) for USP18). After 1h or 2h incubation the fluorescence intensity was recorded using a BMG Labtech PHERAstar plate reader (excitation 487 nm, emission 535 nm). The percentage inhibition was calculated for each well using the following formula:

$$\%inhibition = 100 - 100 \times \frac{FI_x - FI_{pos}}{FI_{neg} - FI_{pos}}$$

Where FI_x is the fluorescence intensity of each well, FI_{pos} is the average fluorescence intensity of the positive (NEM) control wells, and FI_{neg} is the average fluorescence intensity of the negative (DMSO) control wells.

Selectivity determination (Ubiquigent)

DUBprofiler assays were performed by Ubiquigent. BB07CA902 was tested at 0.25 μM using 100 nM ubiquitin-rhodamine110 as substrate.

Covalent Complex Formation Mass Spectrometry Analysis

Samples of 1.4 μM mUSP18 in 20 μL buffer containing 20 mM Tris·HCl, 50 mM NaCl at pH 7.6 and 2.0 mM TCEP were prepared. These samples were treated with 1 μL of vehicle (2.8% DMSO in buffer) or 1 μL of a 280 μM BB07CA902 stock solution in buffer (containing 2.8%

DMSO; final compound concentration = 14 µM) and incubated for 30 min at room temperature. Samples were then diluted 3-fold with water and analyzed by mass spectrometry by injecting 2 µL into a Waters XEVO-G2 XS Q-TOF mass spectrometer equipped with an electrospray ion source in positive mode (capillary voltage 1.2 kV, desolvation gas flow 900 L/h, T = 60 °C) with a resolution of R = 26,000. Samples were run using two mobile phases: (A) 0.1% formic acid in water and (B) 0.1% formic acid in CH_3CN on a Waters Acquity UPLC protein BEH C4 column [300 Å, 1.7 µm (2.1 × 50 mm^2), flow rate = 0.5 mL/min, run time = 14.00 min, column T = 60 °C, and mass detection 200−2500 Da]. Gradient: 2−100% B. Data processing was performed using Waters MassLynx mass spectrometry software 4.1, and ion peaks were deconvoluted using the built-in MaxEnt1 function.

Cell lines and reagents

Human HEK293T (Cat# ATCC® CRL-3216™) and mouse EL4 (Cat# ATCC® TIB-39™) cells were cultured in under standard conditions in Dulbecco's modified Eagle's medium (DMEM) (Gibco) supplemented with 8% FCS (Biowest) and 1% penicillin/streptomycin at 37 °C and 5% CO_2. All cell lines were tested for mycoplasma contamination using MycoAlert™ Mycoplasma Detection Kit (Lonza, Catalog #: LT07-318) on a monthly basis. Interferon β from mouse (mIFN-β) was purchased from Sigma-Aldrich (Cat# I9032).

Murine USP18 overexpression and in-cell inhibition assays

For DNA transfections, HEK293T cells were seeded into 6-well plate to achieve 50–60% confluence the following day and then transfected with plasmids Flag-mUSP18 wildtype (Flag-mUSP18 wt) and Flag-mUSP18 catalytic cysteine-to-alanine mutant (Flag-mUSP18 C61A)[1] using PEI (polyethylenimine, Polysciences Inc., Cat# 23966) as follows: 200 µL DMEM medium without supplements was mixed with DNA and PEI (1 mg/mL) with a ratio of 1:3 (e.g.: 1 µg DNA : 3 µL PEI), incubated at RT for 20 min., and added drop-wise to the cells. At 24 hours following transfection, cells were incubated with indicated compounds at the indicated final concentrations at 37 °C for 4 hours, then washed twice with PBS and harvested for further analysis.

Generation of ISGylation and in-cell inhibition assay

Mouse EL4 cells were stimulated with 300 U/mL mouse IFN-β. At 24 hours following stimulation, cells were incubated with indicated compounds at the indicated final concentrations at 37 °C for 4 hours, then washed twice with PBS and harvested for further analysis.

Activity-based probe labelling

The in-cell inhibition of DUBs and mUSP18 was assessed using activity-based probe labelling assays. In brief, cell pellets were resuspended in HR buffer (50 mM Tris-HCl, 5 mM $MgCl_2$, 250 mM sucrose, 2 mM TCEP and a Protease inhibitor tablet (Roche), pH 7.4), and lysed by sonication (Bioruptor, Diagenode, high intensity for 10 minutes with an ON/OFF cycle of 30 seconds) at 4°C. 40 µg clarified cell lysate was incubated with Rhodamine-mISG15ct-propargylamide probe[1] (final concentration 1 µM) or Rhodamine-Ubiquitin-propargylamide probe[13] (final concentration 1 µM) at 37 °C for 30 min. Reactions were stopped by the addition of LDS (lithium dodecyl sulfate) sample buffer (Invitrogen Life Technologies, Carlsbad, CA, USA) containing 2.5% β-mercaptoethanol, followed by boiling for 7 minutes.

SDS-PAGE, in-gel fluorescence scanning, and immunoblotting

Samples were resolved on precast Bis-Tris NuPAGE Gels (Invitrogen, including 4-12%, and 10% for different samples) using MOPS buffer (Invitrogen Life Technologies, Carlsbad, CA, USA). For fluorescence scan, labeled enzymes were visualized by in-gel fluorescence using a Typhoon FLA 9500 imaging system (GE Healthcare Life Sciences) (Rhodamine channel for probe, Cy5 channel for protein marker).

For immunoblotting, proteins were transferred to a nitrocellulose membrane (Protan BA85, 0.45 µm, GE Healthcare) at 300 mA for 3 hours. The membranes were blocked in 5% milk (skim milk powder, LP0031, Oxiod) in 1× PBS (P1379, Sigma-Aldrich), incubated with a primary antibody diluted in 5% milk in 0.1% PBS-Tween 20 (PBST) for overnight at cold room, washed three times for 5 min in 0.1% PBST, incubated with the secondary antibody diluted in 5% milk in 0.1% PBST for 1 hour, and washed three times again in 0.1% PBST. The signal was visualized using a LICOR Odyssey system. The following primary antibodies were used: mouse anti-Flag (M2, Sigma-Aldrich), rabbit anti-mISG15[14], rabbit anti-mUSP18[15], mouse anti-GAPDH (1D4, Enzo Lifesciences) or mouse anti-β-actin (clone AC-15, Sigma-Aldrich).

The following fluorescent secondary antibodies (from LICOR) were used: anti-mouse-680, anti-rabbit-800.

Cloning NanoBRET plasmids

For NanoBRET experiments, genes of interest were cloned into the NanoBRET Flexi Starter Vectors (Promega, Cat. #N1821), which include pFN21A (N-terminal HaloTag), pFC14K (C-terminal HaloTag), pFN31K (N-terminal NanoLuc) and pFC32K (C-terminal NanoLuc). Cloning was performed using the Flexi Cloning System (Promega, Cat. #R1851 and #R1901) according to the manufacturer´s instructions.

Pre-cloned pFN21A vectors containing human ISG15, USP18 and STAT2 sequences were obtained directly from Promega and subsequently subcloned into the additional NanoBRET vectors (pFN31K, pFC14K and pFC32K). Murine ISG15 and USP18 sequences were amplified by PCR using Q5 High-Fidelity DNA Polymerase (New England Biolabs) according to the manufacturer´s instructions and primers sets fwd_mUSP18/rev_mUSP18 and fwd_mISG15/rev_mISG15, respectively. Amplified products were first cloned into the pFN21A vector and then subcloned into the other vectors of the NanoBRET system.

Murine and human ISG15 were cloned only into the pFN21A and pFN31K vectors, as ISG15 undergoes C-terminal processing.

Primers	Sequences
fwd_mUSP18	AAAAAAGCGATCGCCATGGGCAAGGGGTTTG
rev_mUSP18	AAAAAA GTTTAAAC GGATCCAGTCTTCGTGTAAAC
fwd_mISG15	AAAAAA GCGATCGC CGGGCCCTCTAGAATG
rev_mISG15	GTACCGAGCTCGGATCCTTAC GTTTAAACTTTTTT

NanoBRET assays

For each protein pair, all possible plasmid combinations were initially tested to determine the pair yielding the highest corrected BRET signal. Transient NanoBRET assays were performed following the manufacturer's instructions (Promega). Briefly, HEK293T cells were transfected with NanoLuc and HaloTag fusion vectors at 1:100 ratio (20 ng NanoLuc and 2 µg HaloTag plasmid). After approx. 20 h, cells were harvested, resuspended in phenol red–free Opti-MEM media supplemented with 4% FCS, and plated in white, flat-bottom, 96-well tissue culture plates (Greiner). Cells were treated with HaloTag NanoBRET 618 Ligand (Promega) or DMSO and incubated for 4–6 h at 37 °C with 5% CO_2. Immediately before signal detection,

NanoBRET Nano-Glo substrate (Promega) was added, and luminescence was recorded using a Tecan Spark 10M Multimode Plate Reader at 415–485 nm (NanoLuc donor) and 610–700 nm (HaloTag acceptor). The corrected NanoBRET ratio (mBU) was calculated by dividing the acceptor signal by the donor signal and subtracting the background signal obtained from the DMSO vehicle control from that of the HaloTag NanoBRET 618 Ligand treated samples.

Plasmid combinations that produced the highest corrected NanoBRET ratios were selected for donor saturation assays. In these experiments, HEK293T cells were transiently transfected with a fixed amount of donor plasmid (NanoLuc fusion vector) and increasing amounts of acceptor plasmid (HaloTag fusion vector). As a negative control, a HaloTag empty vector (Promega) was co-transfected with the NanoLuc fusion vector using the same donor-to-acceptor ratios. To ensure equal total DNA across all conditions, empty pcDNA3.1(-) plasmid was added where necessary. Data were analyzed using GraphPad Prism v10.5.0 to assess curve fitting. Saturation binding was evaluated using a one-site specific binding model, where a fitted curve indicated protein–protein interaction, while a linear fit indicated no interaction.

For interacting protein pairs, the Kd value (half-maximal saturation) obtained from the binding curve was used to define the optimal donor-to-acceptor ratio (assay window) for subsequent compound disruption assays. HEK293T cells were transfected with the corresponding amounts of NanoLuc and HaloTag fusion vectors and treated with increasing concentrations (0.01–10 µM) of compounds BB06CA902 or BB07CA902. Equivalent volumes of DMSO were included as vehicle controls to exclude solvent-related effects. To assess potential nonspecific effects, the p53-MDM2 negative control pair (Promega) was also treated with the compounds. Data were analyzed in GraphPad Prism v10.5.0, and inhibition was assessed by fitting to a dose–response model.

Cell viability assay

Cell viability was assessed using the CellTiter-Glo® 2.0 Cell Viability Assay (Promega). Immediately after measuring the NanoBRET signal, the reagent was added directly to the wells following the manufacturer’s instructions. Total luminescence was recorded using a Tecan Spark 10M plate reader, and cell viability was expressed as relative luminescence units (RLU) normalized to vehicle-treated controls.

Inhibition of human deISGylation activity

SUPT1 WT and USP18 KO cells (kindly provided by Prof. Dr. med. Speck) were stimulated with 500 U/mL hIFNβ (Peprotech) for 24 h. Cells were then treated with BB06CA902 or BB07CA902 (0.1–100 μM) for 4 h, pelleted, and lysed in buffer containing 1% CHAPS, 20 mM HEPES (pH 7.4), 8 mM EDTA, 2 mM EGTA, 2× complete protease inhibitor, and 10 mM NEM. Cell lysates were subjected to SDS–PAGE and transferred onto PVDF membranes for western blotting. Membranes were blocked and incubated with an anti-β-actin (8H10D10) antibody (Cell Signalling, 1:1000) and a homemade antibody against human ISG15 (1:1000).

References

[1] A. Basters, P. P. Geurink, A. Rocker, K. F. Witting, R. Tadayon, S. Hess, M. S. Semrau, P. Storici, H. Ovaa, K. P. Knobeloch, G. Fritz, *Nat. Struct. Mol. Biol.* **2017**, *24*, 270-278.

[2] R. Kooij, S. H. Liu, A. Sapmaz, B. T. Xin, G. M. C. Janssen, P. A. van Veelen, H. Ovaa, P. ten Dijke, P. P. Geurink, *J. Am. Chem. Soc.* **2020**, *142*, 16825-16841.

[3] A. Jones, M. Kemp, M. Stockley, K. Gibson, G. Whitlock, *WO2016/156816 A1* **2016**.

[4] A. Jones, M. I. Kemp, M. L. Stockley, K. R. Gibson, G. A. Whitlock, A. Madin, *WO2016/046530 A1* **2016**.

[5] R. C. Lemoine, A. C. Petersen, L. Setti, T. Baldinger, J. Wanner, A. Jekle, G. Heilek, A. deRosier, C. H. Ji, D. M. Rotstein, *Bioorg. Med. Chem. Lett.* **2010**, *20*, 1674-1676.

[6] G. Lesma, A. Sacchetti, A. Silvani, *Synthesis* **2006**, 594-596.

[7] C. N. Larsen, J. S. Price, K. D. Wilkinson, *Biochem.* **1996**, *35*, 6735-6744.

[8] M. H. Nanao, S. O. Tcherniuk, J. Chroboczek, O. Dideberg, A. Dessen, M. Y. Balakirev, *EMBO Rep.* **2004**, *5*, 783-788.

[9] R. Q. Kim, P. P. Geurink, M. P. C. Mulder, A. Fish, R. Ekkebus, F. El Oualid, W. J. van Dijk, D. van Dalen, H. Ovaa, H. van Ingen, T. K. Sixma, *Nat. Commun.* **2019**, *10*, 231.

[10] E. Mons, R. Q. Kim, B. R. van Doodewaerd, P. A. van Veelen, M. P. C. Mulder, H. Ovaa, *J. Am. Chem. Soc.* **2021**, *143*, 6423-6433.

[11] A. Basters, P. P. Geurink, F. El Oualid, L. Ketscher, M. S. Casutt, E. Krause, H. Ovaa, K. P. Knobeloch, G. Fritz, *FEBS J.* **2014**, *281*, 1918-1928.

[12] A. Sapmaz, I. Berlin, E. Bos, R. H. Wijdeven, H. Janssen, R. Konietzny, J. J. Akkermans, A. E. Erson-Bensan, R. I. Koning, B. M. Kessler, J. Neefjes, H. Ovaa, *Nat. Commun.* **2019**, *10*.

[13] R. Ekkebus, S. I. van Kasteren, Y. Kulathu, A. Scholten, I. Berlin, P. P. Geurink, A. de Jong, S. Goerdayal, J. Neefjes, A. J. Heck, D. Komander, H. Ovaa, *J. Am. Chem. Soc.* **2013**, *135*, 2867-2870.

[14] A. Osiak, O. Utermöhlen, S. Niendorf, I. Horak, K. P. Knobeloch, *Mol. Cell. Biol.* **2005**, *25*, 6338-6345.

[15] L. Ketscher, R. Hannss, D. J. Morales, A. Basters, S. Guerra, T. Goldmann, A. Hausmann, M. Prinz, R. Naumann, A. Pekosz, O. Utermohlen, D. J. Lenschow, K. P. Knobeloch, *Proc. Natl. Acad. Sci. USA* **2015**, *112*, 1577-1582.

[16] T. E. T. Mevissen, M. K. Hospenthal, P. P. Geurink, P. R. Elliott, M. Akutsu, N. Arnaudo, R. Ekkebus, Y. Kulathu, T. Wauer, F. El Oualid, S. M. Freund, H. Ovaa, D. Komander, *Cell* **2013**, *154*, 169-184.

[17] Y. Huppelschoten, R. Mukhopadhyay, J. Buchardt, T. E. Nielsen, A. C. O. Vertegaal, H. Ovaa, G. J. van der Heden van Noort, *ChemBioChem* **2023**, *24*, e202200601.

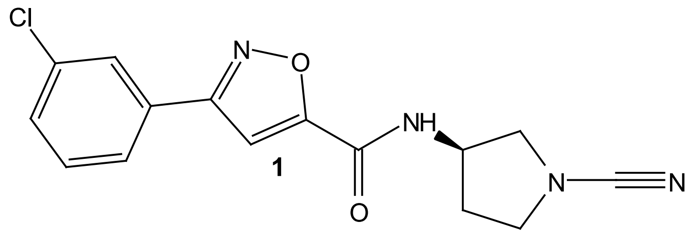

Cl
N
O
NH
1
O
N
N

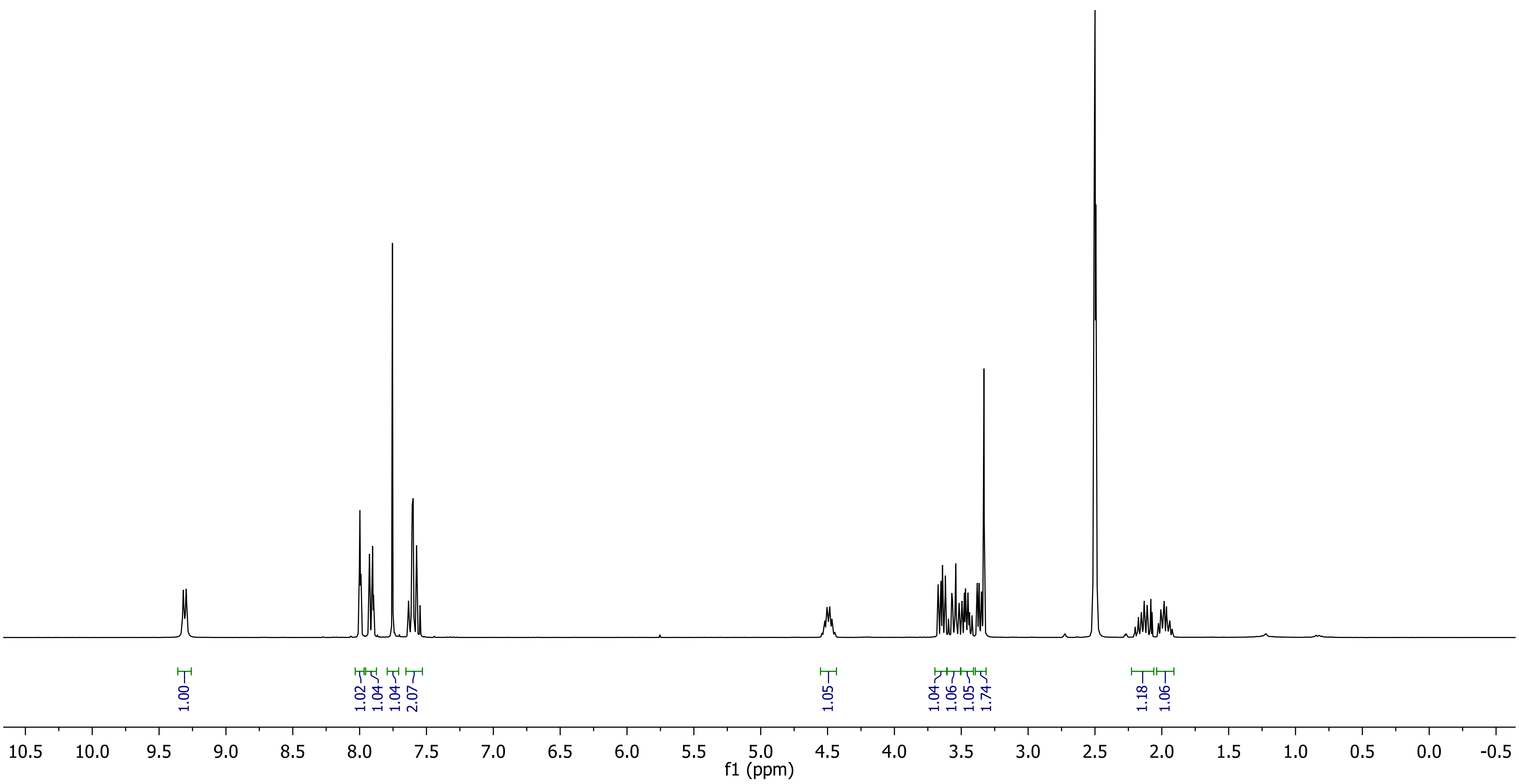

1.00
1.02
1.04
1.04
2.07
1.05
1.04
1.06
1.05
1.74
1.18
1.06
10.5
10.0
9.5
9.0
8.5
8.0
7.5
7.0
6.5
6.0
5.5
5.0
4.5
4.0
3.5
3.0
2.5
2.0
1.5
1.0
0.5
0.0
-0.5
f1 (ppm)

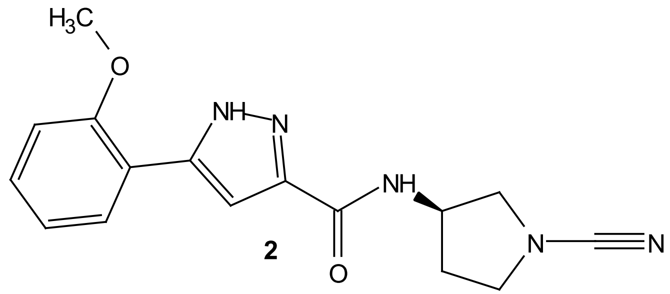
H3C
O
NH
N
NH
N
N
O
2

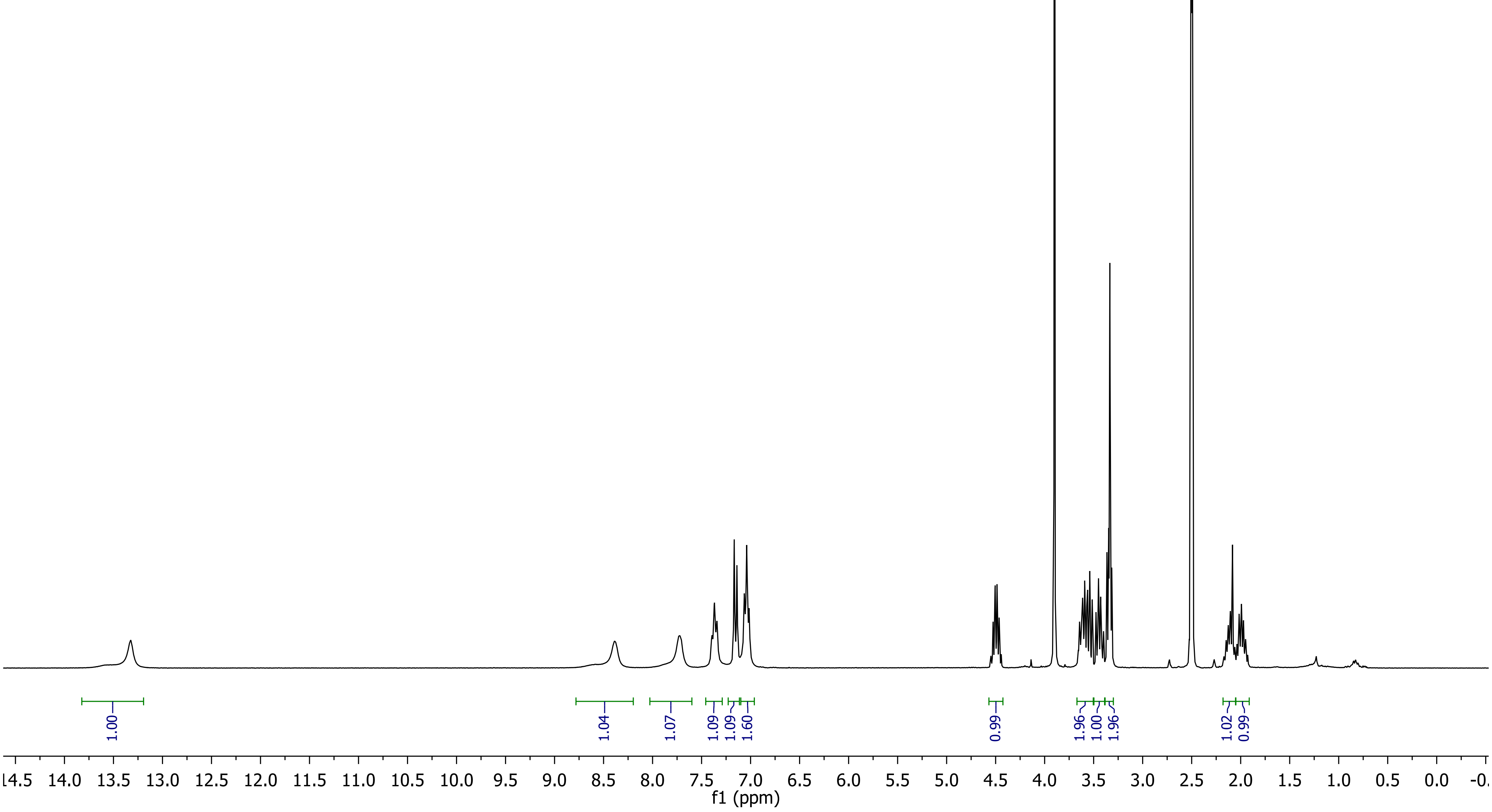
1.00
1.04
1.07
1.09
1.09
1.60
0.99
1.96
1.00
1.96
1.02
0.99
14.5 14.0 13.5 13.0 12.5 12.0 11.5 11.0 10.5 10.0 9.5 9.0 8.5 8.0 7.5 7.0 6.5 6.0 5.5 5.0 4.5 4.0 3.5 3.0 2.5 2.0 1.5 1.0 0.5 0.0
f1 (ppm)

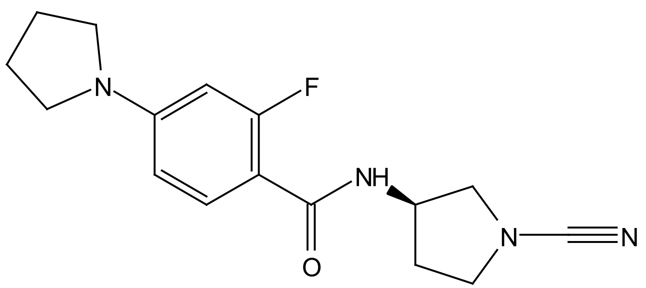
N
F
NH
N
N
O

3

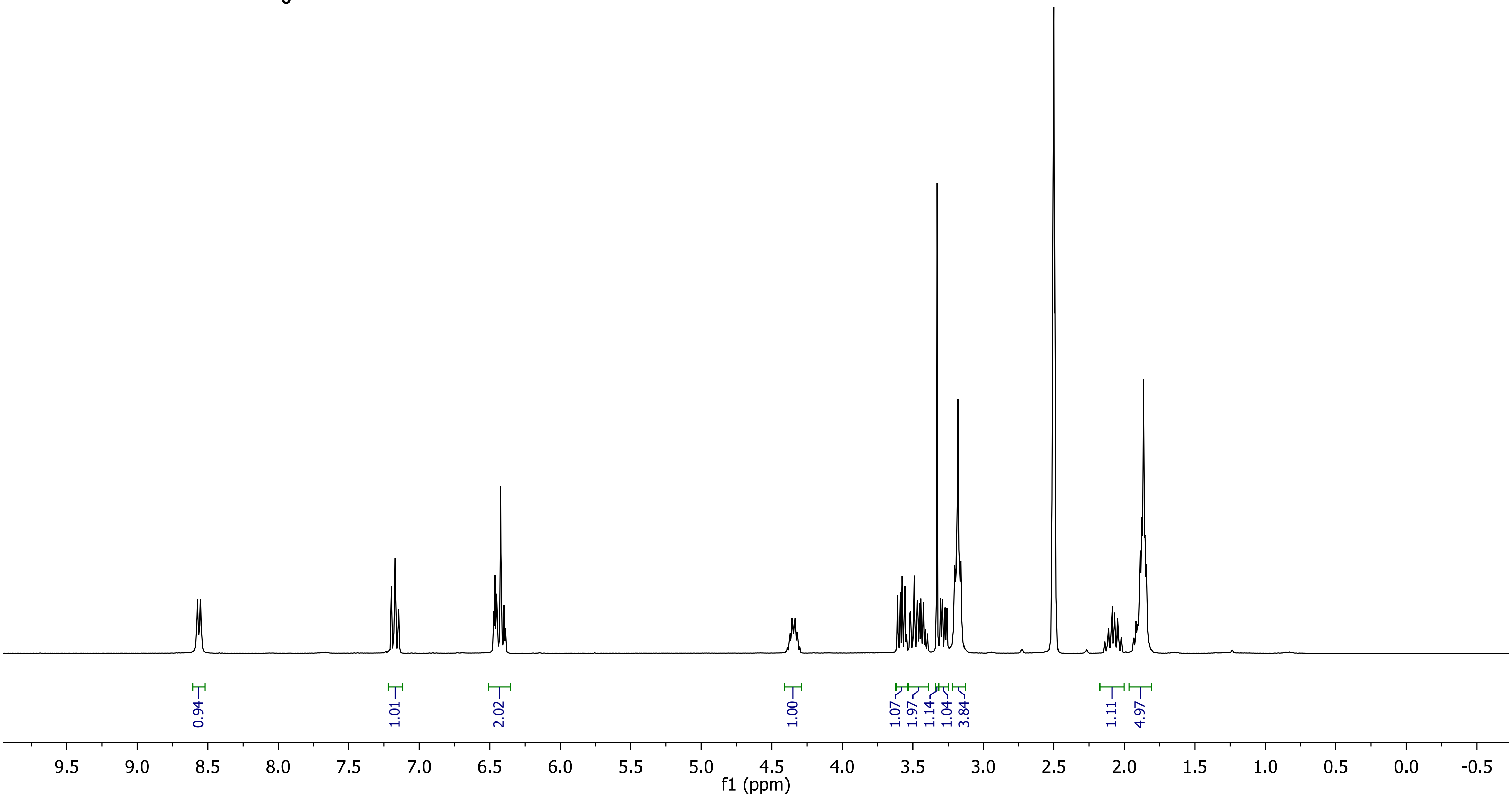
0.94
1.01
2.02
1.00
1.07
1.97
1.14
1.04
3.84
1.11
4.97
9.5 9.0 8.5 8.0 7.5 7.0 6.5 6.0 5.5 5.0 4.5 4.0 3.5 3.0 2.5 2.0 1.5 1.0 0.5 0.0 -0.5
f1 (ppm)

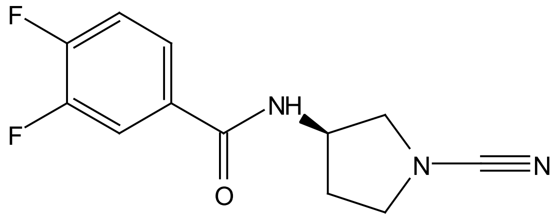
F
F
NH
O
N
N

4

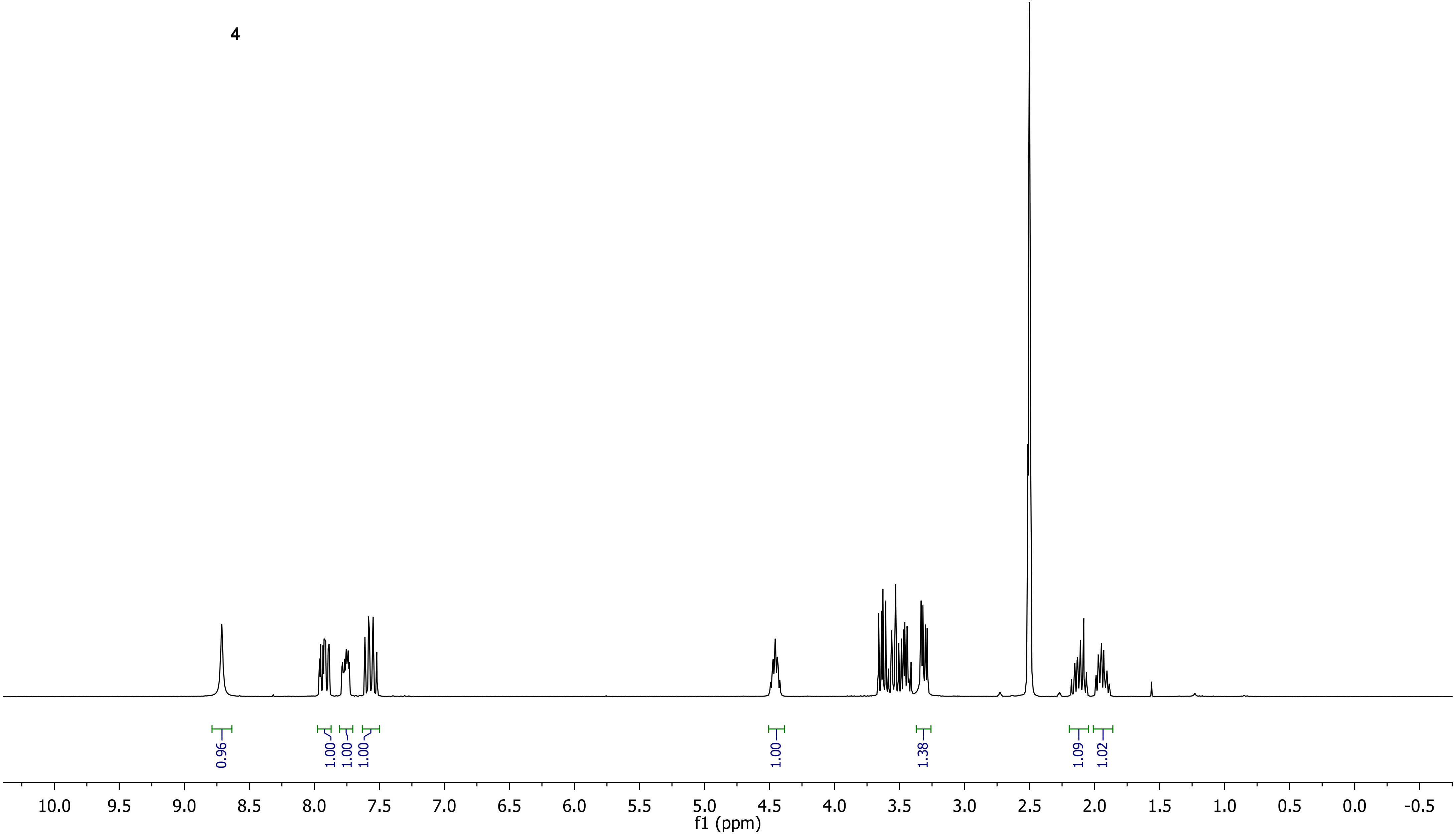
0.96
1.00
1.00
1.00
1.00
1.38
1.09
1.02
10.0 9.5 9.0 8.5 8.0 7.5 7.0 6.5 6.0 5.5 5.0 4.5 4.0 3.5 3.0 2.5 2.0 1.5 1.0 0.5 0.0 -0.5
f1 (ppm)

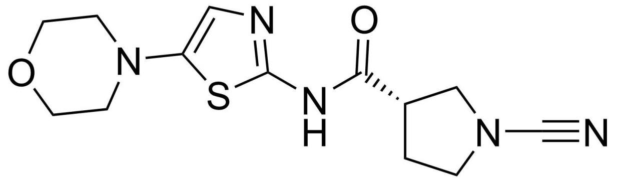

5

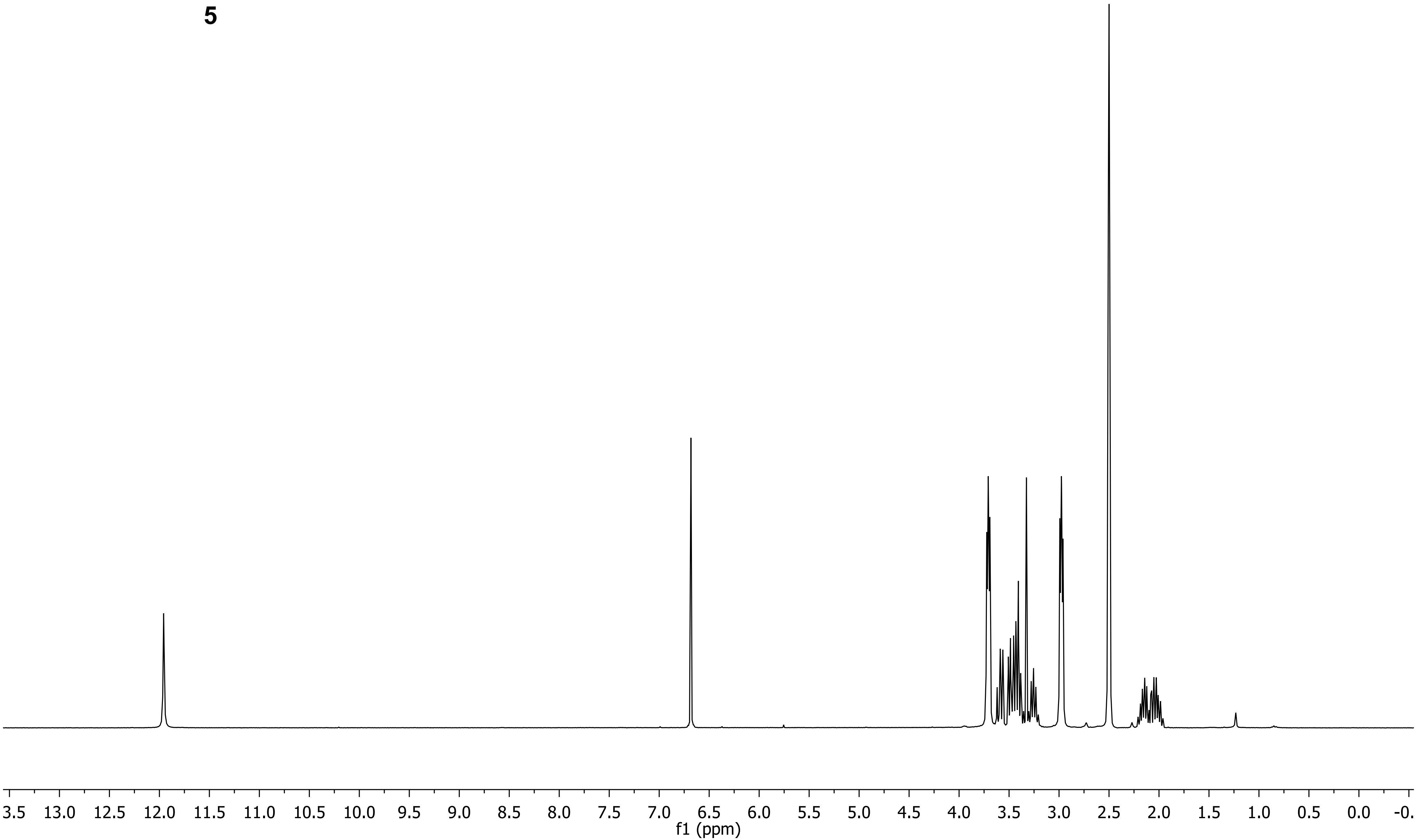

3.5 13.0 12.5 12.0 11.5 11.0 10.5 10.0 9.5 9.0 8.5 8.0 7.5 7.0 6.5 6.0 5.5 5.0 4.5 4.0 3.5 3.0 2.5 2.0 1.5 1.0 0.5 0.0 -0.
f1 (ppm)

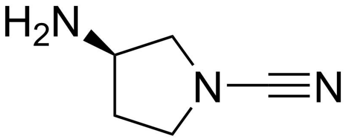

BB1

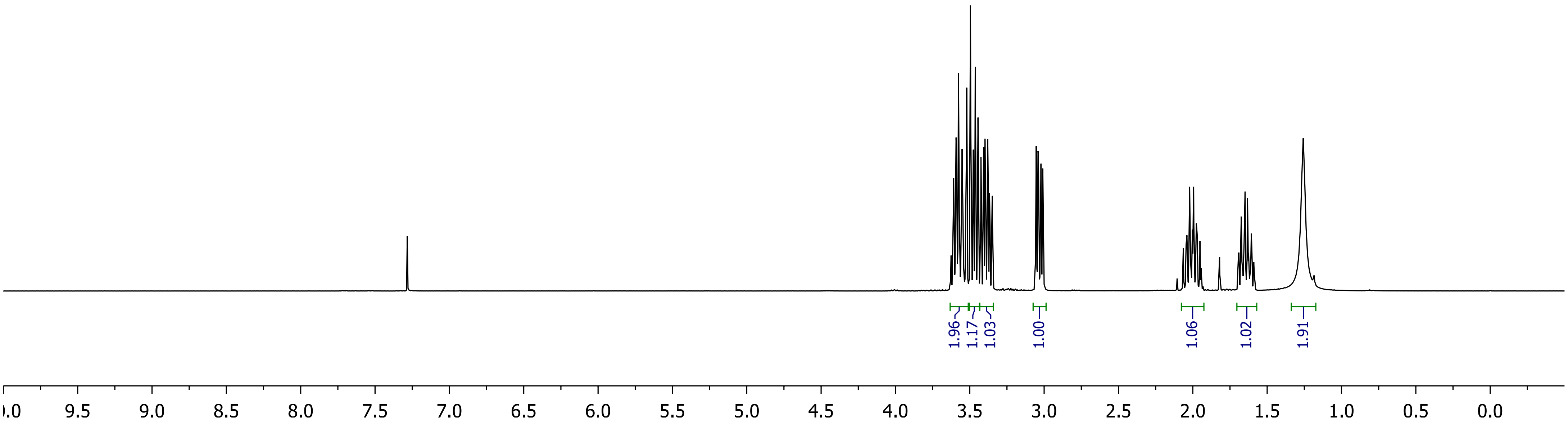
1.96
1.17
1.03
1.00
1.06
1.02
1.91
9.5
9.0
8.5
8.0
7.5
7.0
6.5
6.0
5.5
5.0
4.5
4.0
3.5
3.0
2.5
2.0
1.5
1.0
0.5
0.0

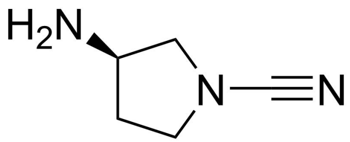

BB1

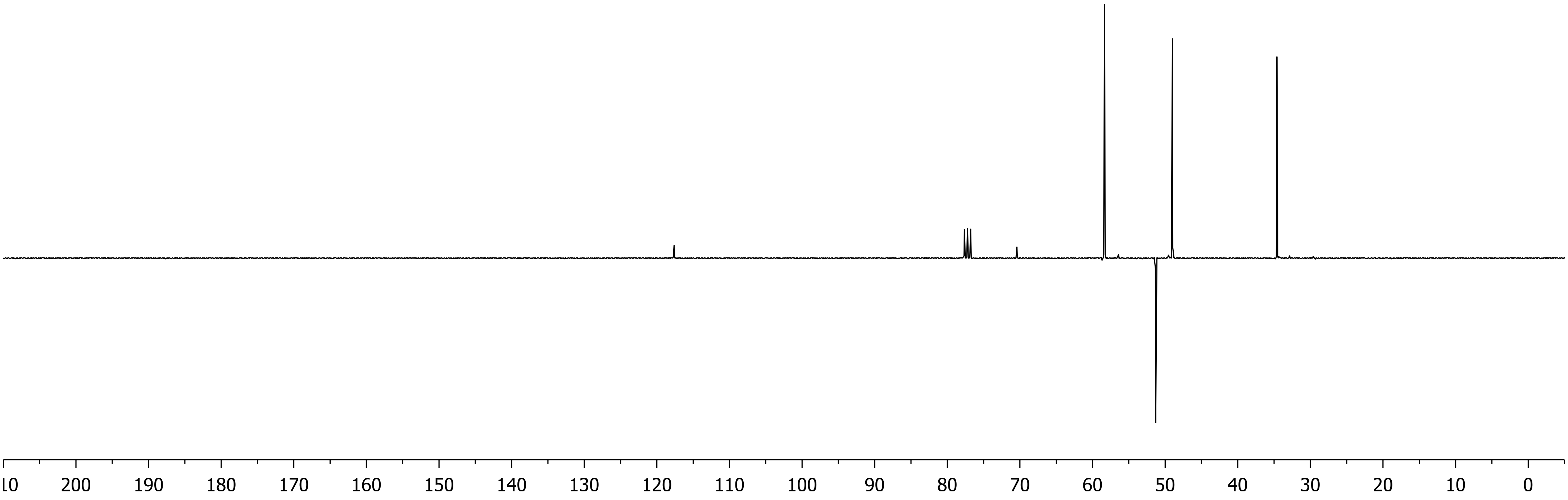
200
190
180
170
160
150
140
130
120
110
100
90
80
70
60
50
40
30
20
10
0

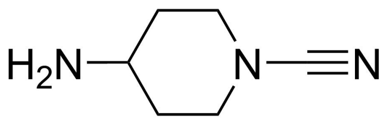
H2N
N
N

BB2

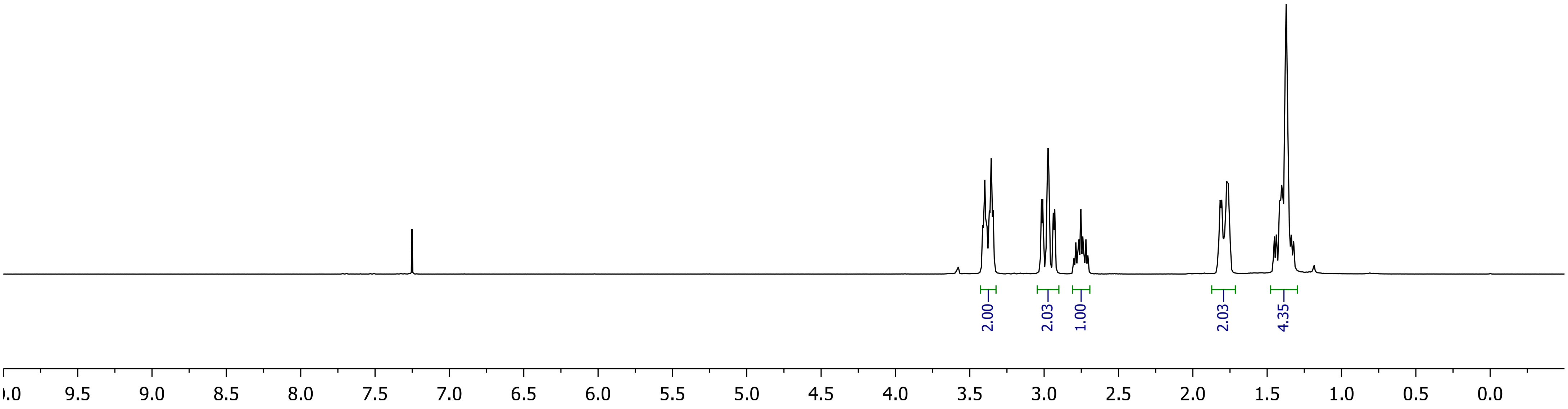
2.00
2.03
1.00
2.03
4.35
9.5
9.0
8.5
8.0
7.5
7.0
6.5
6.0
5.5
5.0
4.5
4.0
3.5
3.0
2.5
2.0
1.5
1.0
0.5
0.0

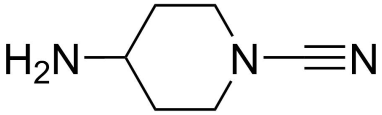
H2N
N
N

BB2

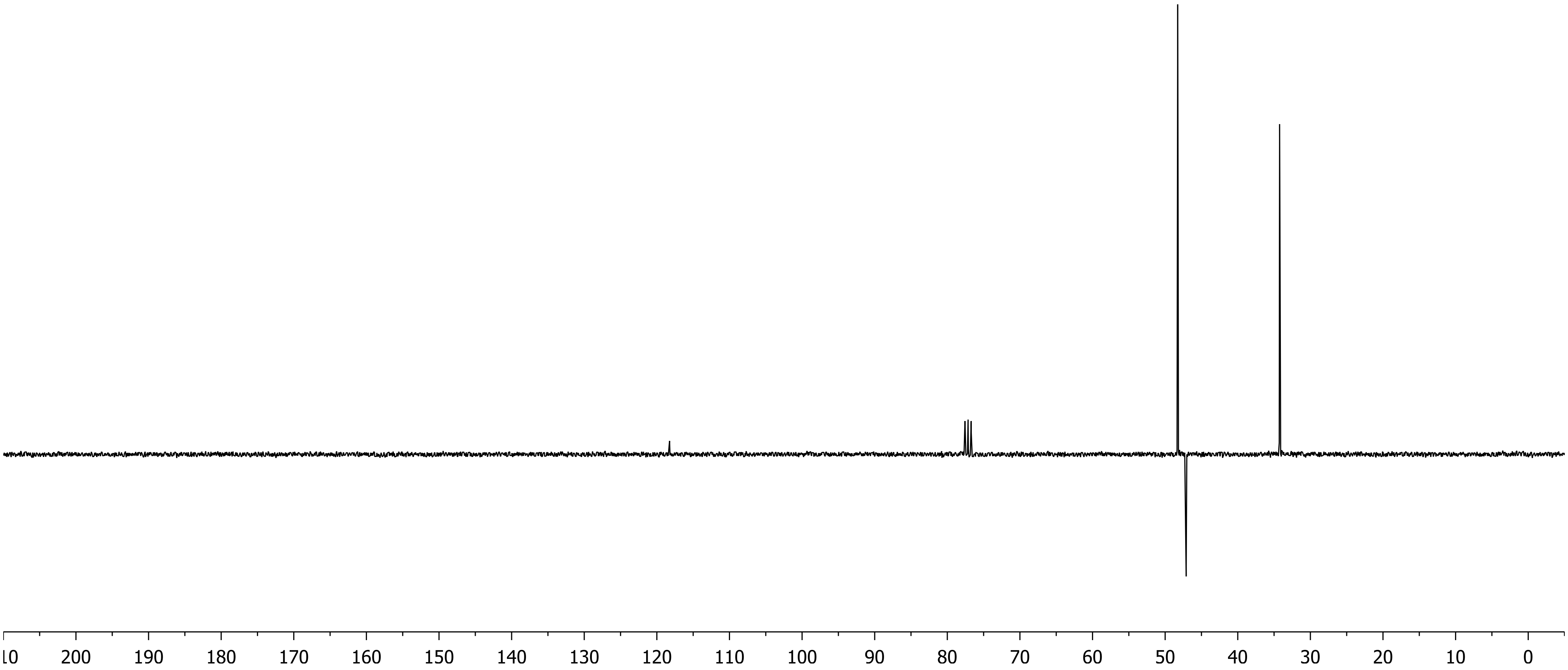
10
200
190
180
170
160
150
140
130
120
110
100
90
80
70
60
50
40
30
20
10
0

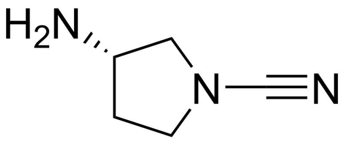
H2N
N
N

BB3

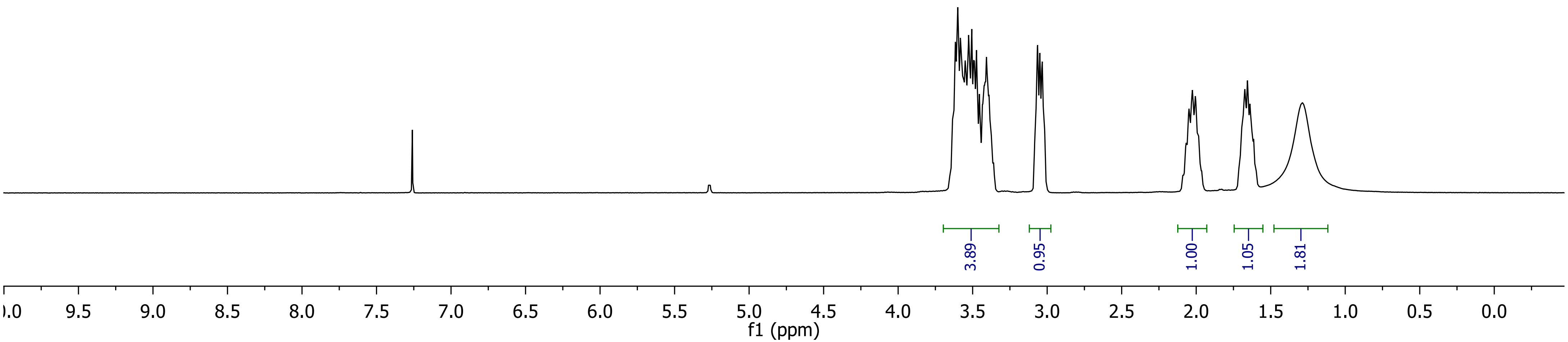
3.89
0.95
1.00
1.05
1.81
9.5
9.0
8.5
8.0
7.5
7.0
6.5
6.0
5.5
5.0
4.5
4.0
3.5
3.0
2.5
2.0
1.5
1.0
0.5
0.0
f1 (ppm)

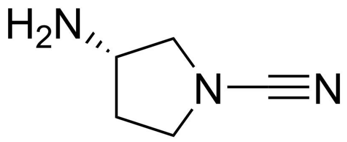

BB3

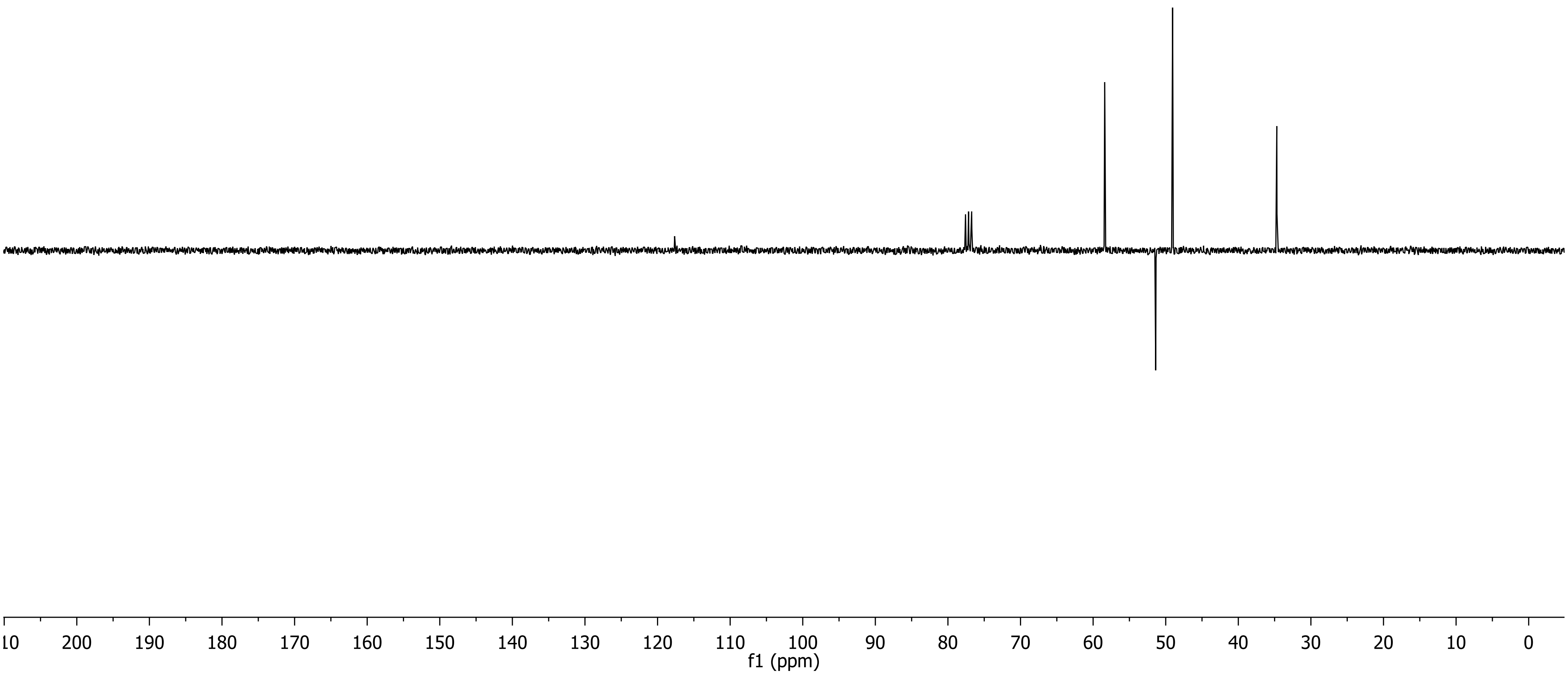
200
190
180
170
160
150
140
130
120
110
100
90
80
70
60
50
40
30
20
10
0
f1 (ppm)

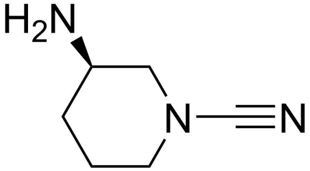
H2N
N
N

BB4

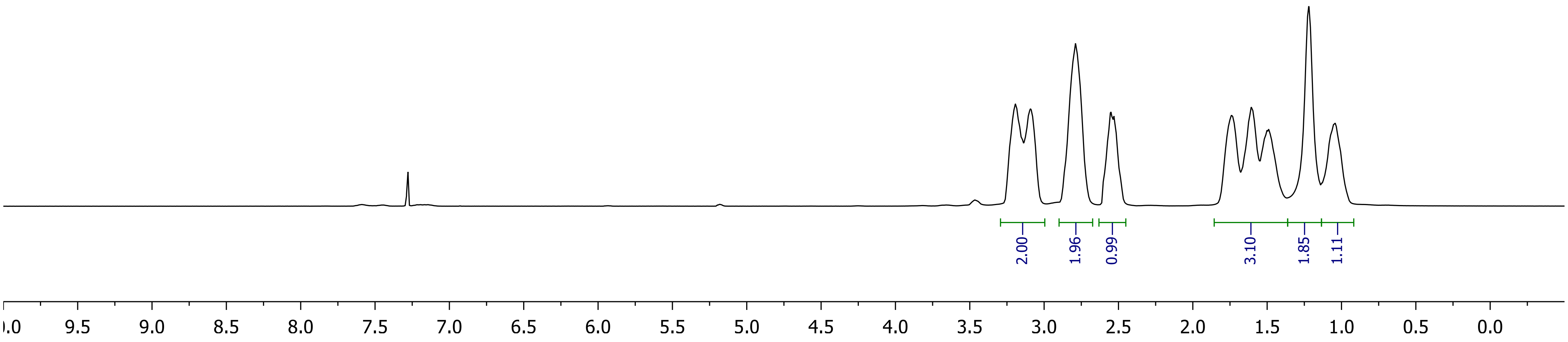
2.00
1.96
0.99
3.10
1.85
1.11
9.5
9.0
8.5
8.0
7.5
7.0
6.5
6.0
5.5
5.0
4.5
4.0
3.5
3.0
2.5
2.0
1.5
1.0
0.5
0.0

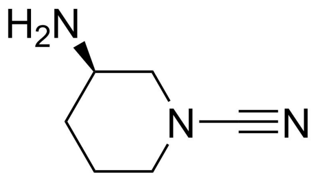

BB4

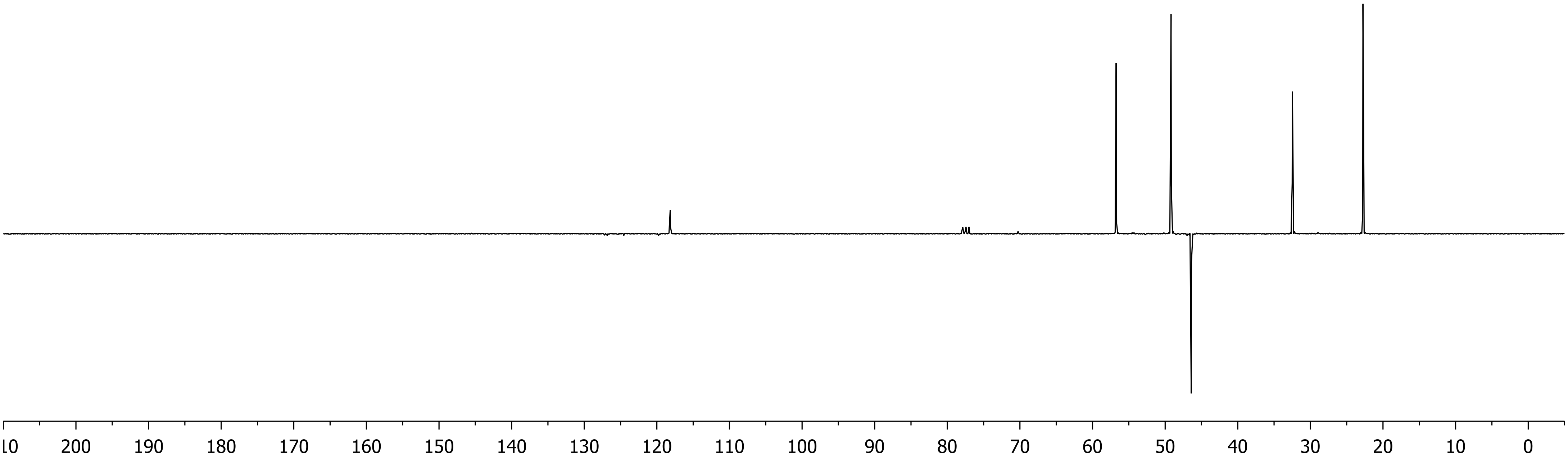

200
190
180
170
160
150
140
130
120
110
100
90
80
70
60
50
40
30
20
10
0

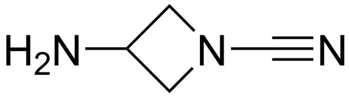
H2N
N
N

BB5

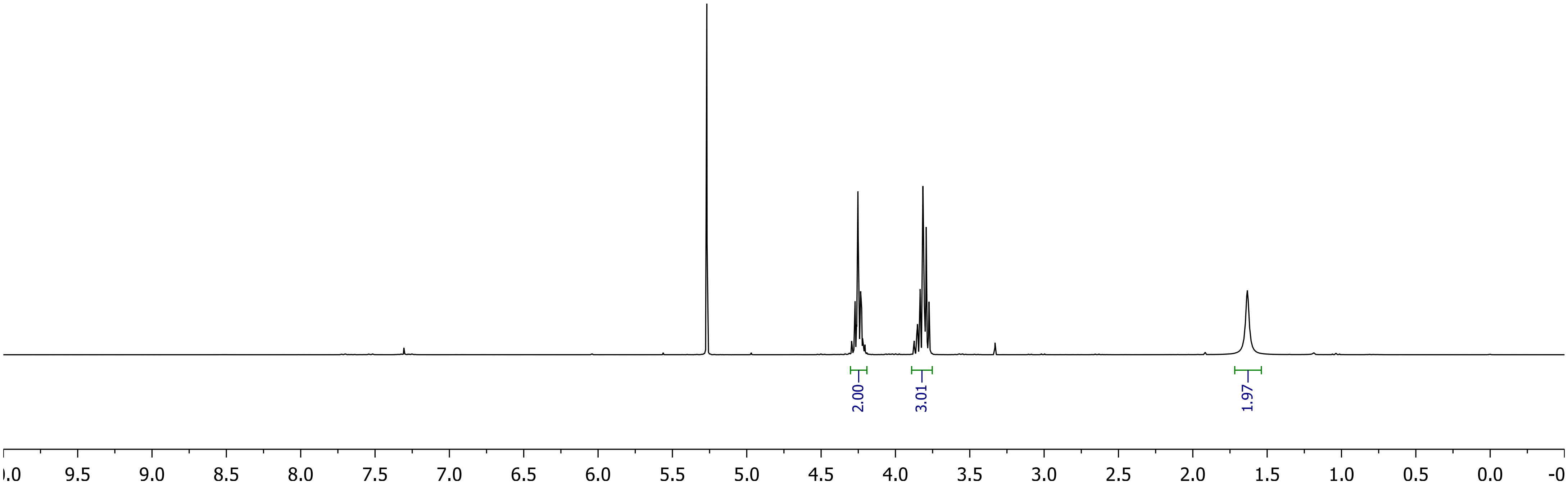
2.00
3.01
1.97
.0
9.5
9.0
8.5
8.0
7.5
7.0
6.5
6.0
5.5
5.0
4.5
4.0
3.5
3.0
2.5
2.0
1.5
1.0
0.5
0.0
-0

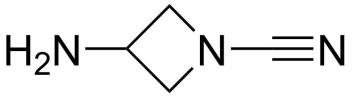

BB5

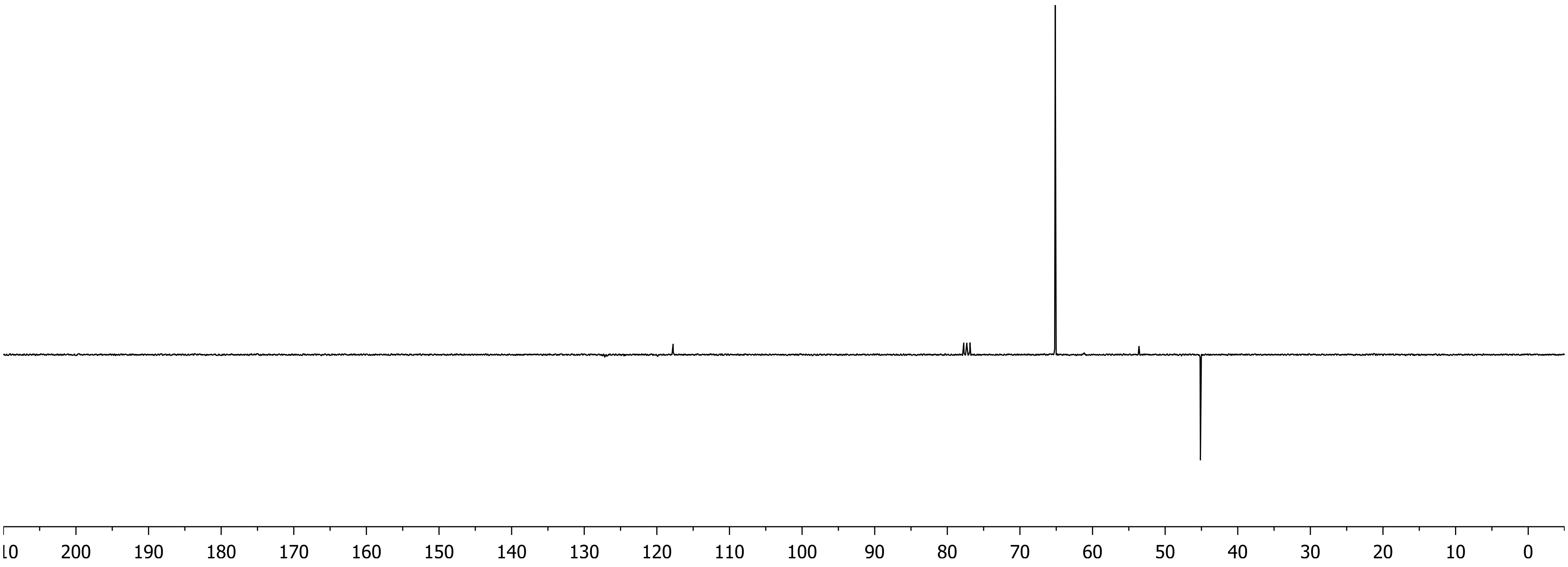
200 190 180 170 160 150 140 130 120 110 100 90 80 70 60 50 40 30 20 10 0

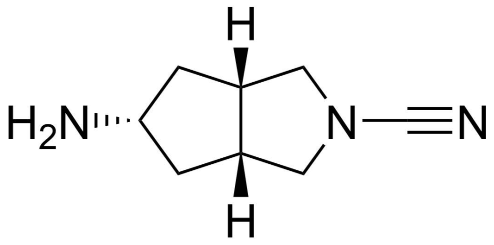
H
H2N
N
N
H

BB6

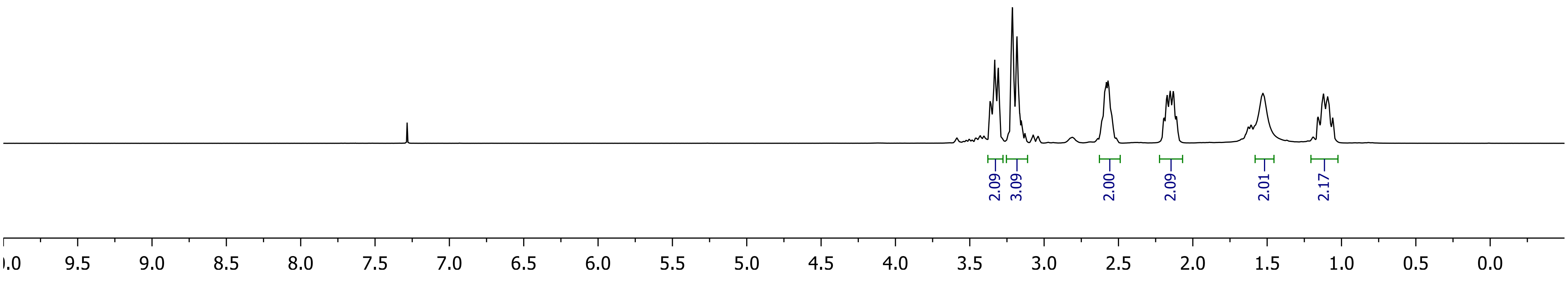
2.09
3.09
2.00
2.09
2.01
2.17
.0
9.5
9.0
8.5
8.0
7.5
7.0
6.5
6.0
5.5
5.0
4.5
4.0
3.5
3.0
2.5
2.0
1.5
1.0
0.5
0.0

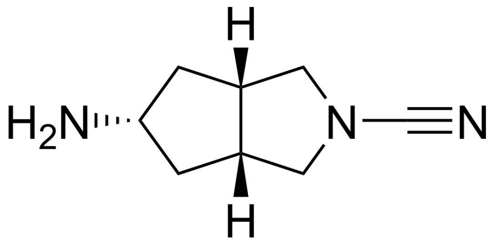
H
H2N
N
N
H

BB6

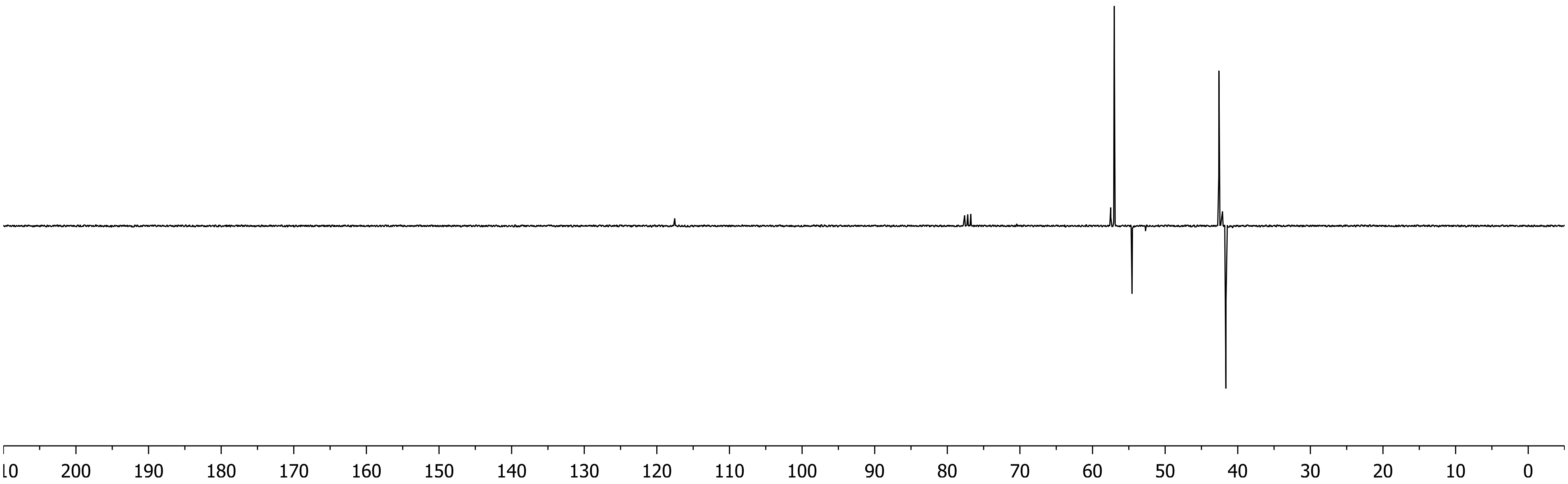
200
190
180
170
160
150
140
130
120
110
100
90
80
70
60
50
40
30
20
10
0

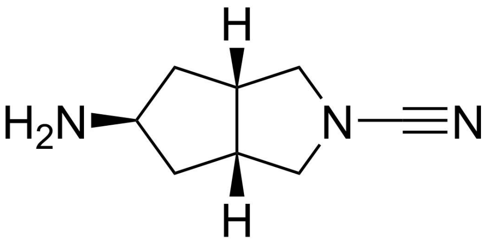
H
H₂N
N
N
H

BB7

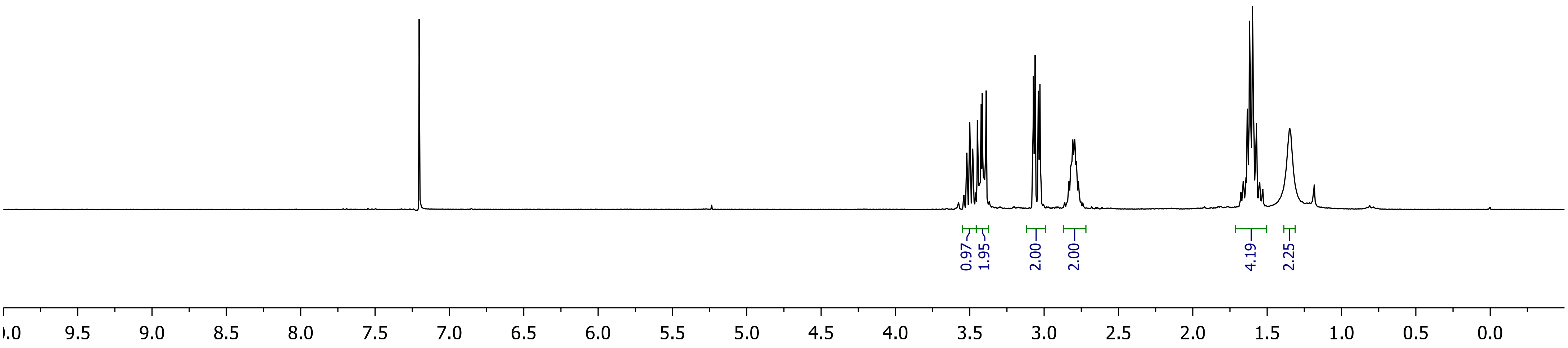
0.97
1.95
2.00
2.00
4.19
2.25
9.5 9.0 8.5 8.0 7.5 7.0 6.5 6.0 5.5 5.0 4.5 4.0 3.5 3.0 2.5 2.0 1.5 1.0 0.5 0.0

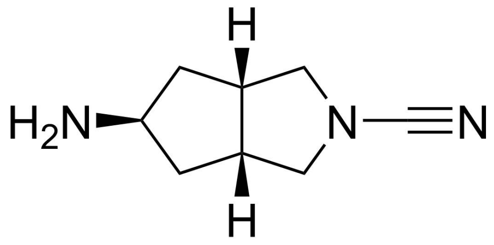

BB7

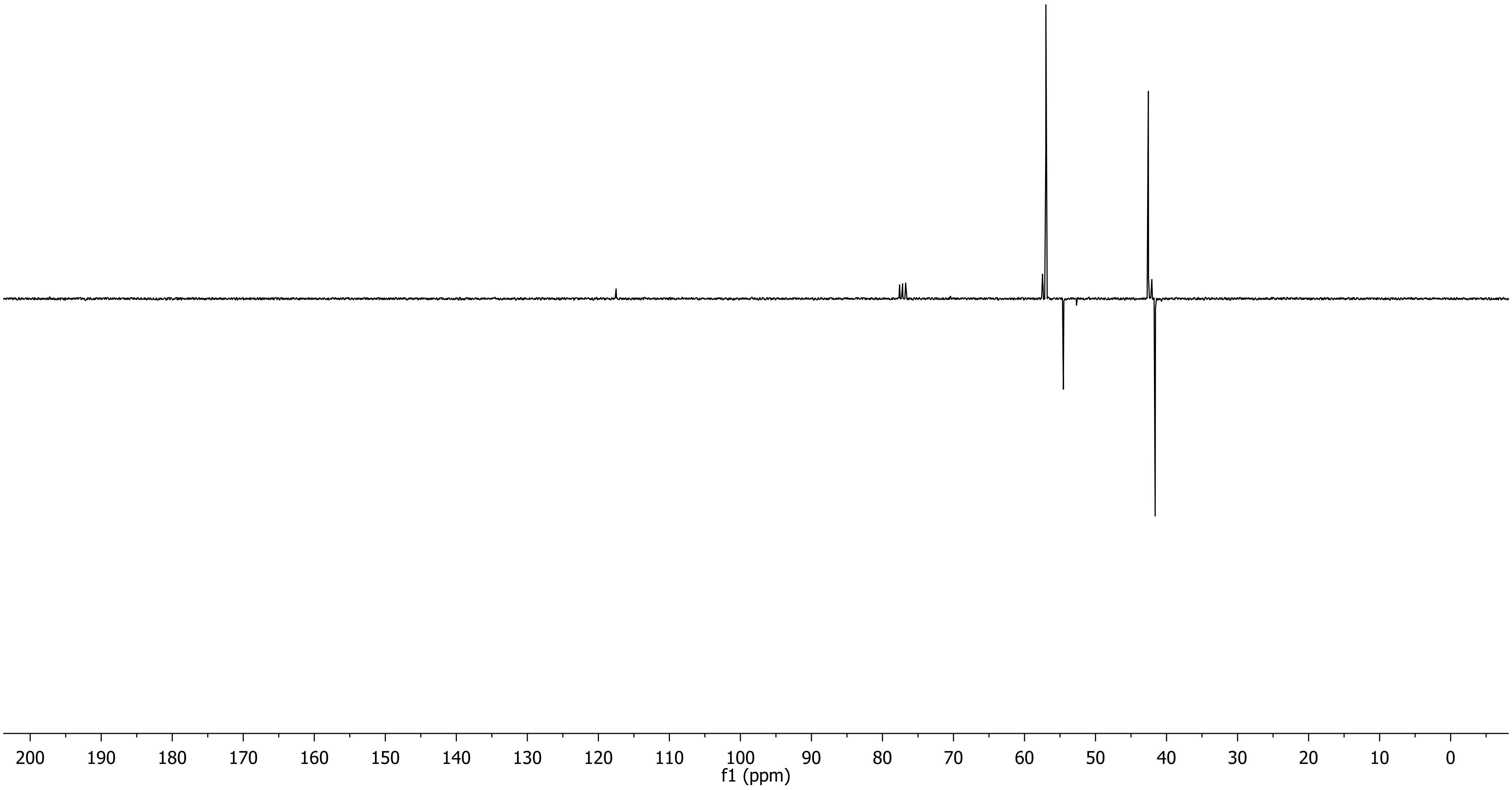
200
190
180
170
160
150
140
130
120
110
100
90
80
70
60
50
40
30
20
10
0
f1 (ppm)

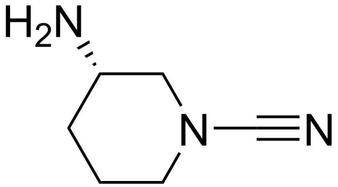
H2N
N
N

BB8

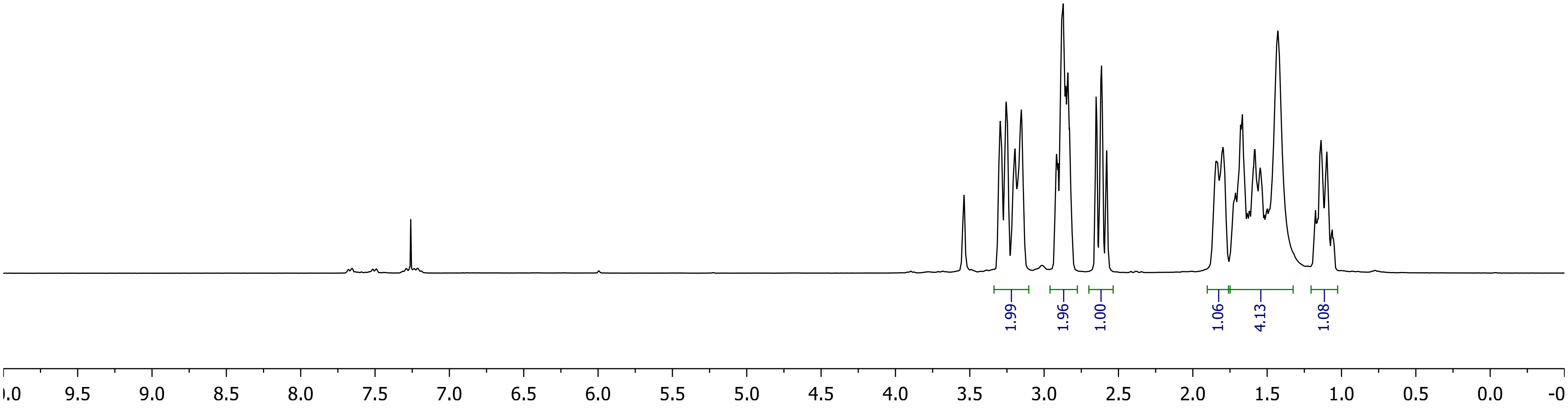
1.99
1.96
1.00
1.06
4.13
1.08
9.5
9.0
8.5
8.0
7.5
7.0
6.5
6.0
5.5
5.0
4.5
4.0
3.5
3.0
2.5
2.0
1.5
1.0
0.5
0.0

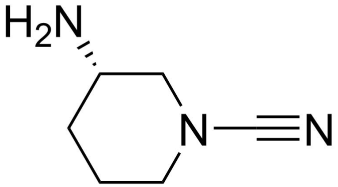
H2N
N
N

BB8

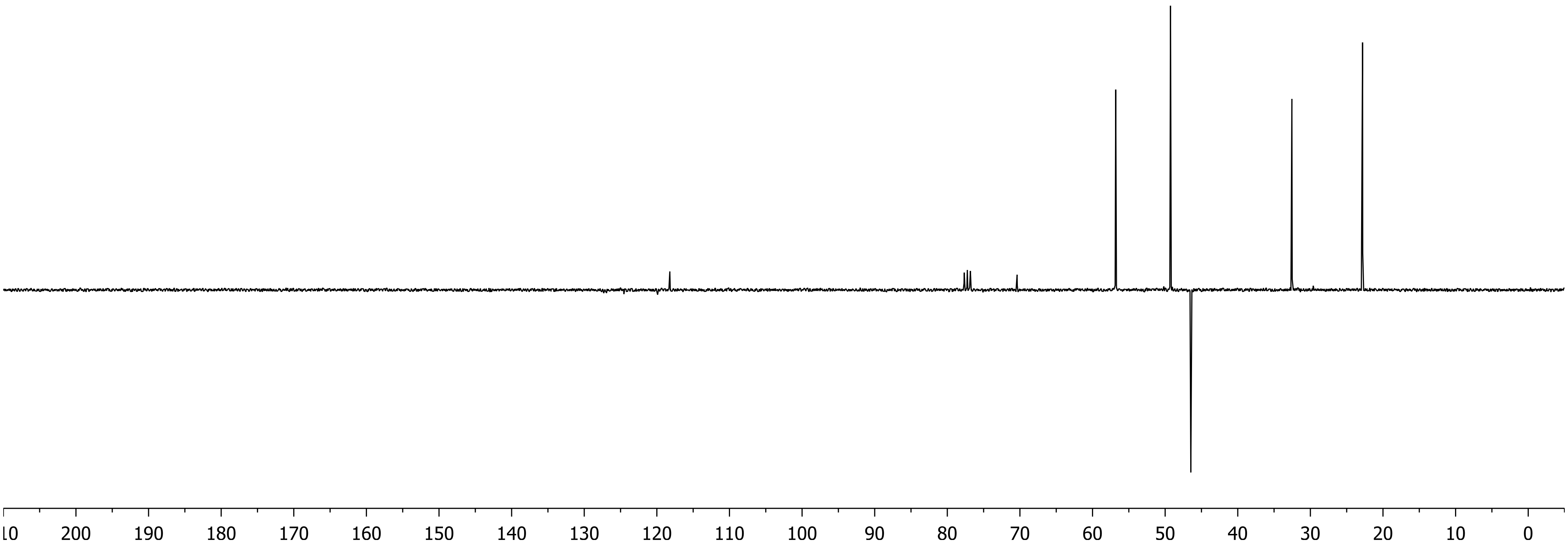
200
190
180
170
160
150
140
130
120
110
100
90
80
70
60
50
40
30
20
10
0

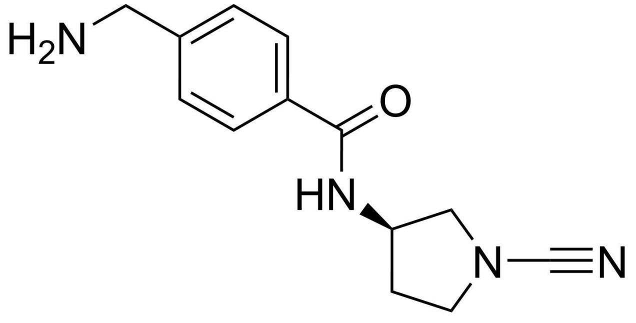

H2N
O
HN
N
N

BB9

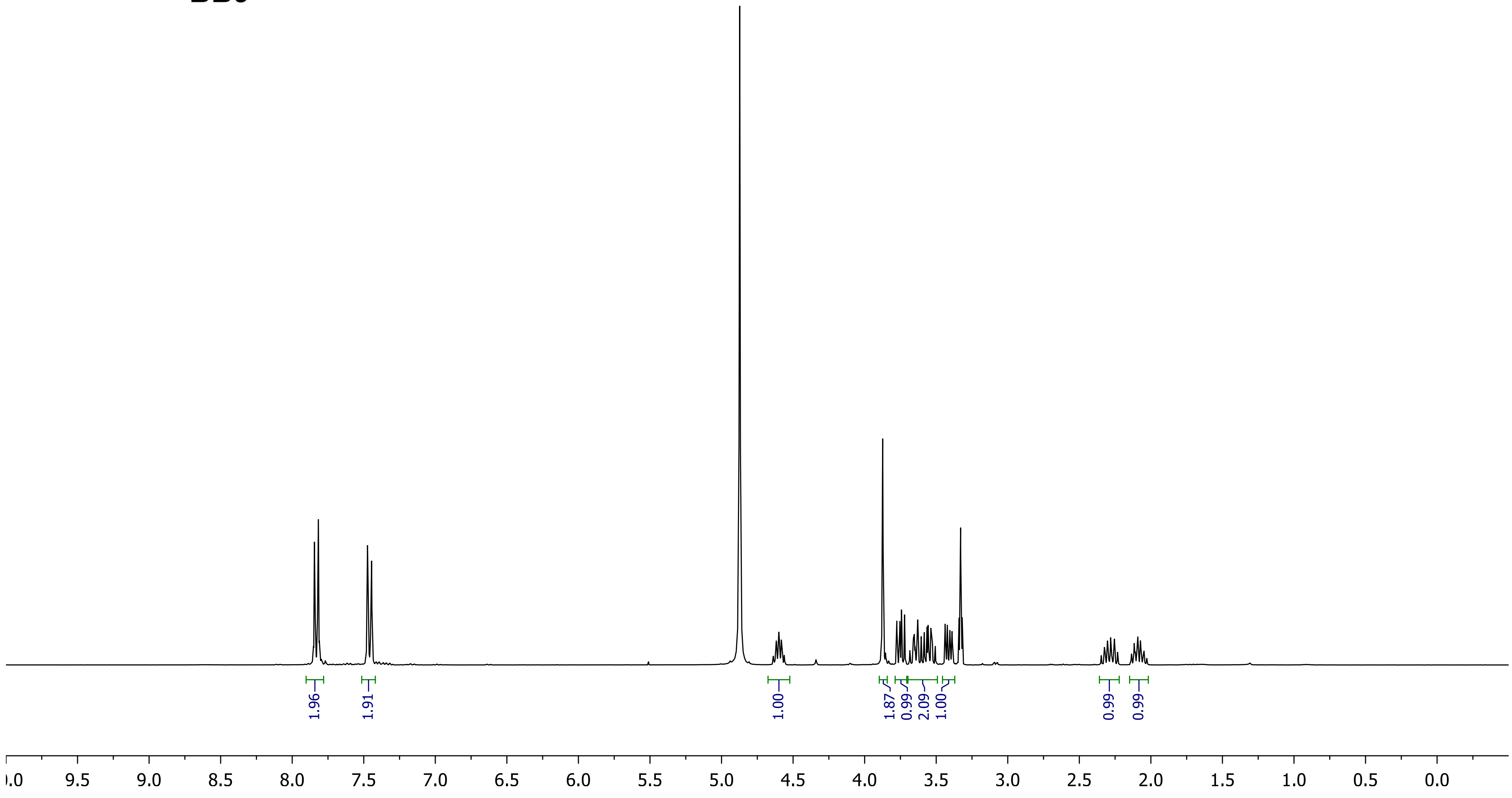

1.96
1.91
1.00
1.87
0.99
2.09
1.00
0.99
0.99
9.5
9.0
8.5
8.0
7.5
7.0
6.5
6.0
5.5
5.0
4.5
4.0
3.5
3.0
2.5
2.0
1.5
1.0
0.5
0.0

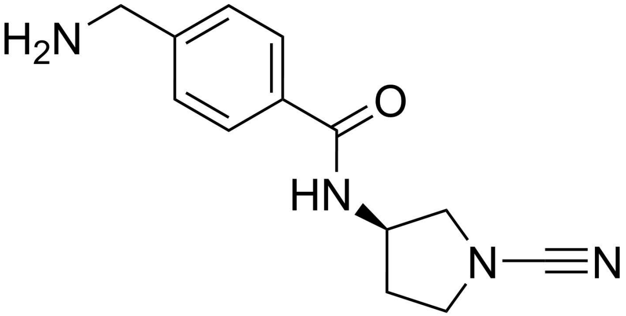
H2N
O
HN
N
N

BB9

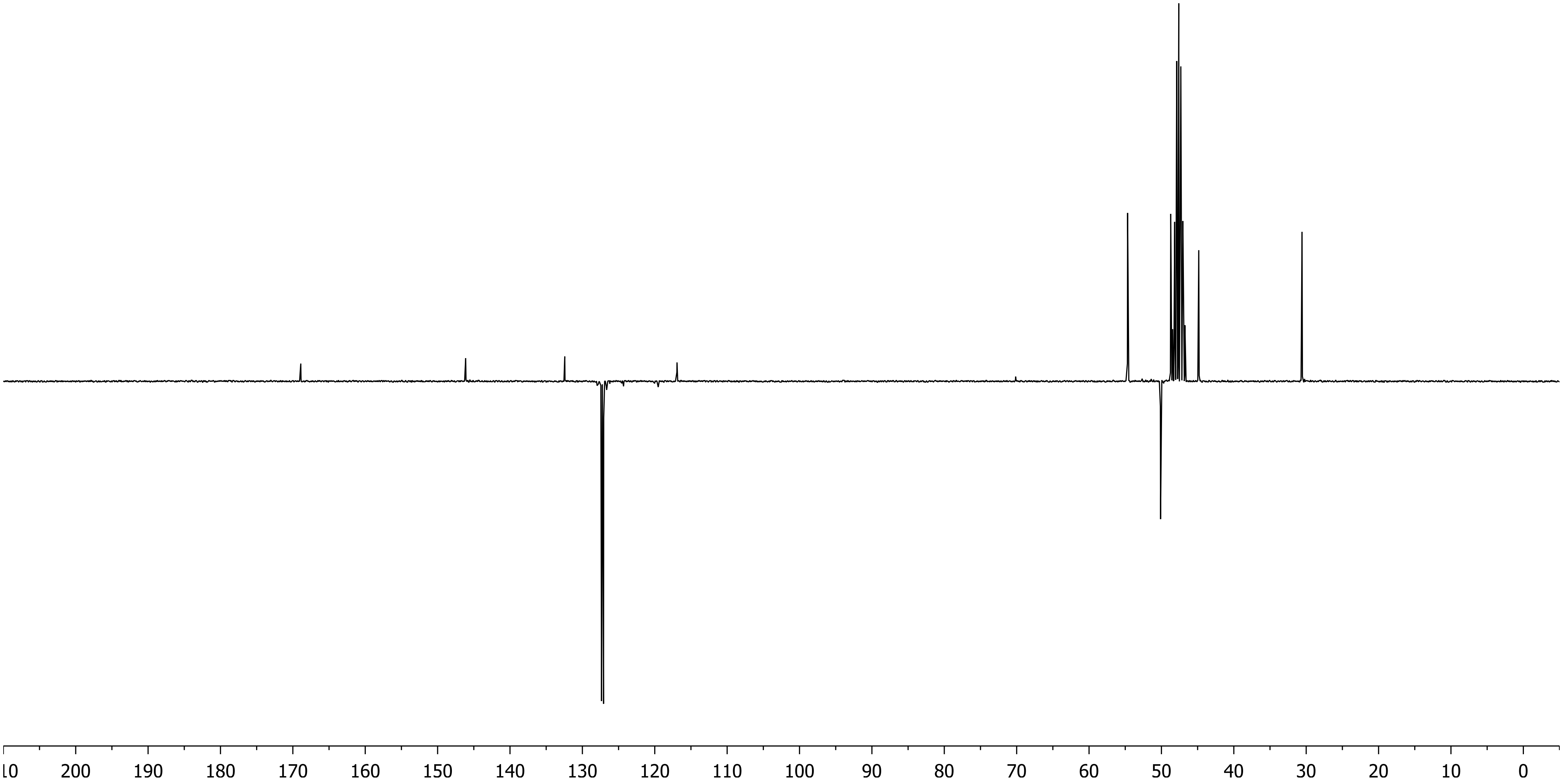
200
190
180
170
160
150
140
130
120
110
100
90
80
70
60
50
40
30
20
10
0

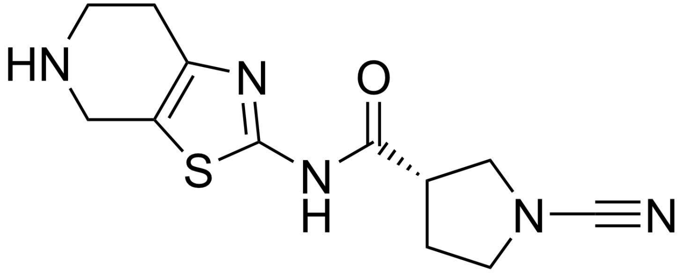

BB10

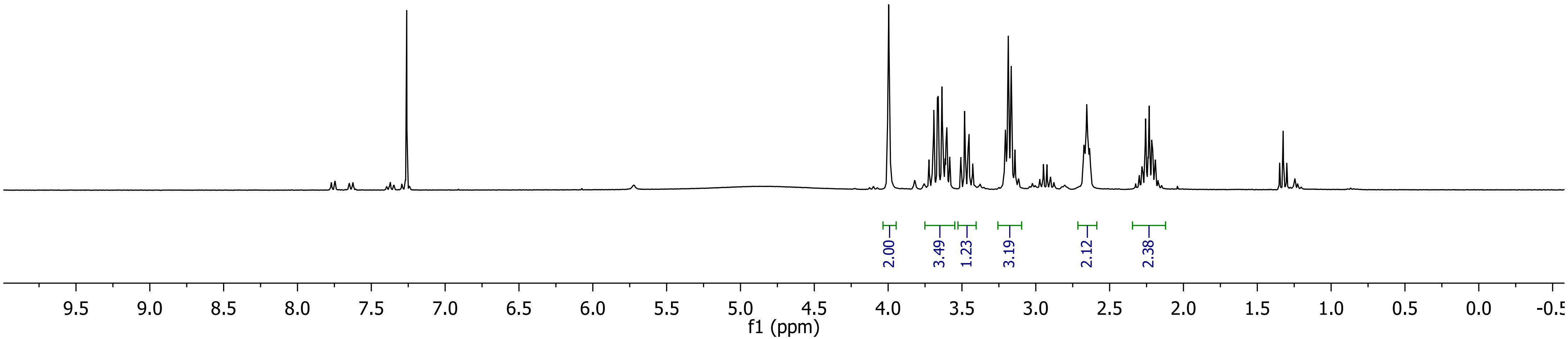
2.00
3.49
1.23
3.19
2.12
2.38
9.5 9.0 8.5 8.0 7.5 7.0 6.5 6.0 5.5 5.0 4.5 4.0 3.5 3.0 2.5 2.0 1.5 1.0 0.5 0.0 -0.5
f1 (ppm)

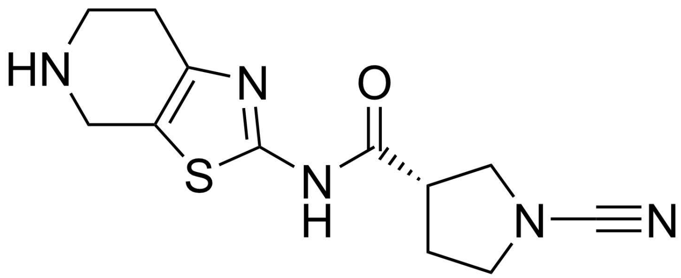
HN
N
O
S
N
H
N
N

BB10

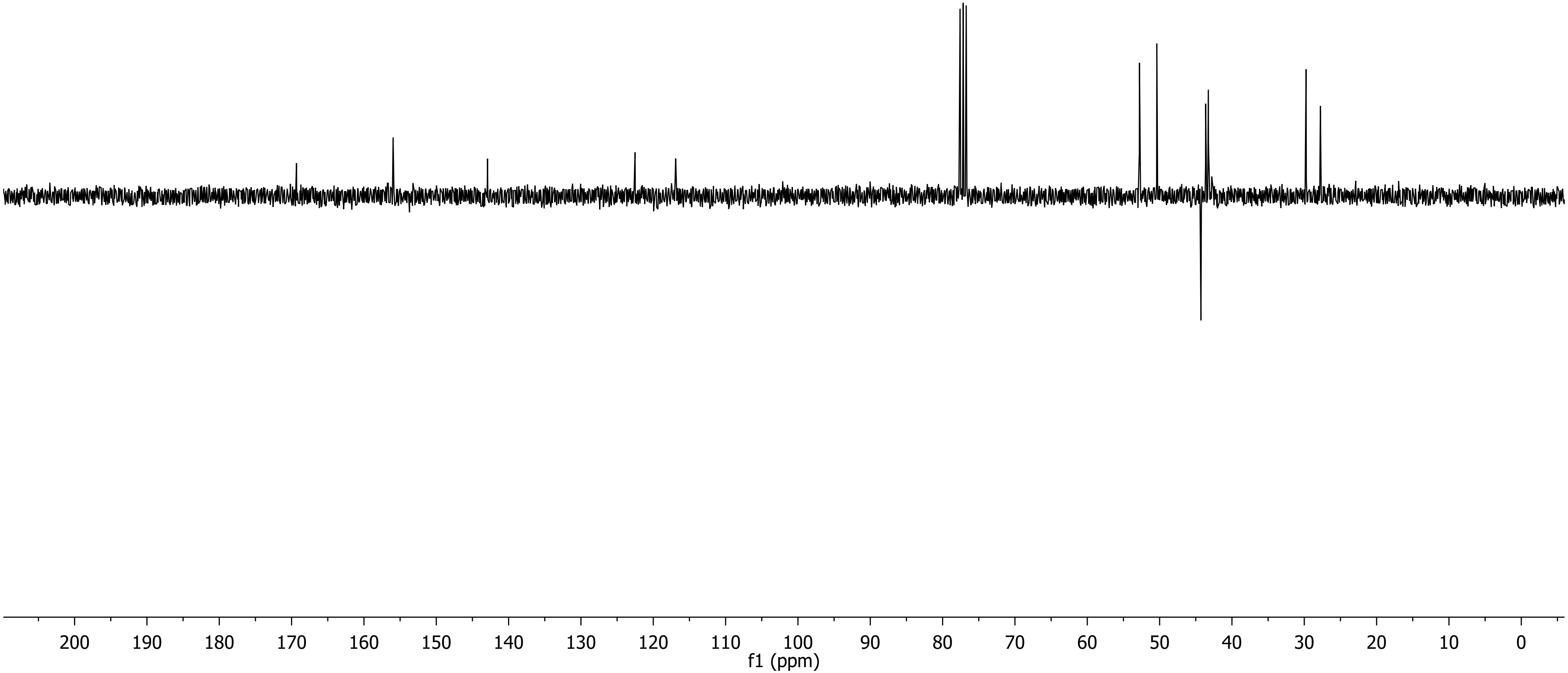
200
190
180
170
160
150
140
130
120
110
100
90
80
70
60
50
40
30
20
10
0
f1 (ppm)

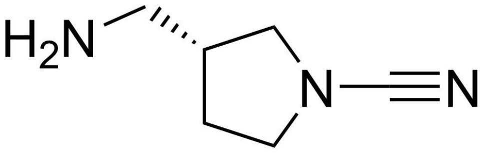

BB11

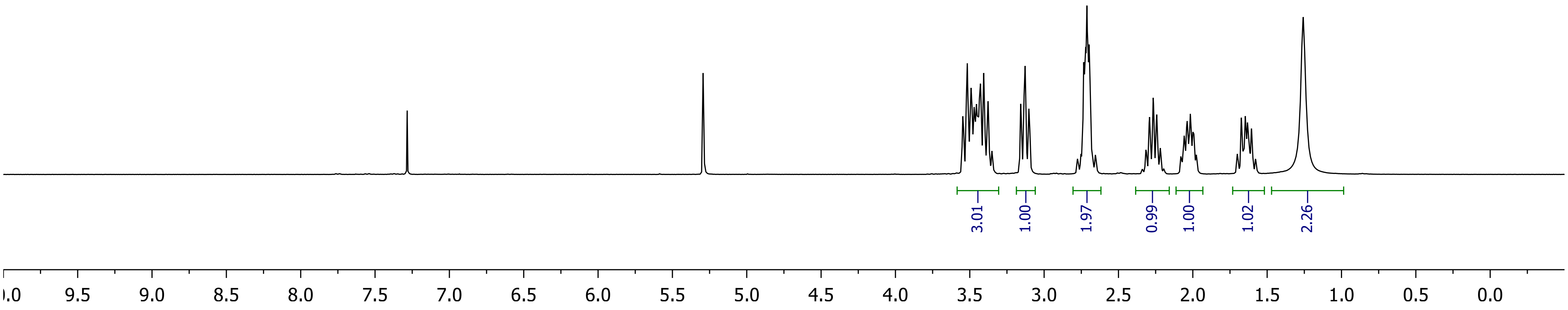
3.01
1.00
1.97
0.99
1.00
1.02
2.26
9.5 9.0 8.5 8.0 7.5 7.0 6.5 6.0 5.5 5.0 4.5 4.0 3.5 3.0 2.5 2.0 1.5 1.0 0.5 0.0

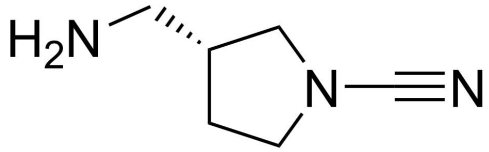

BB11

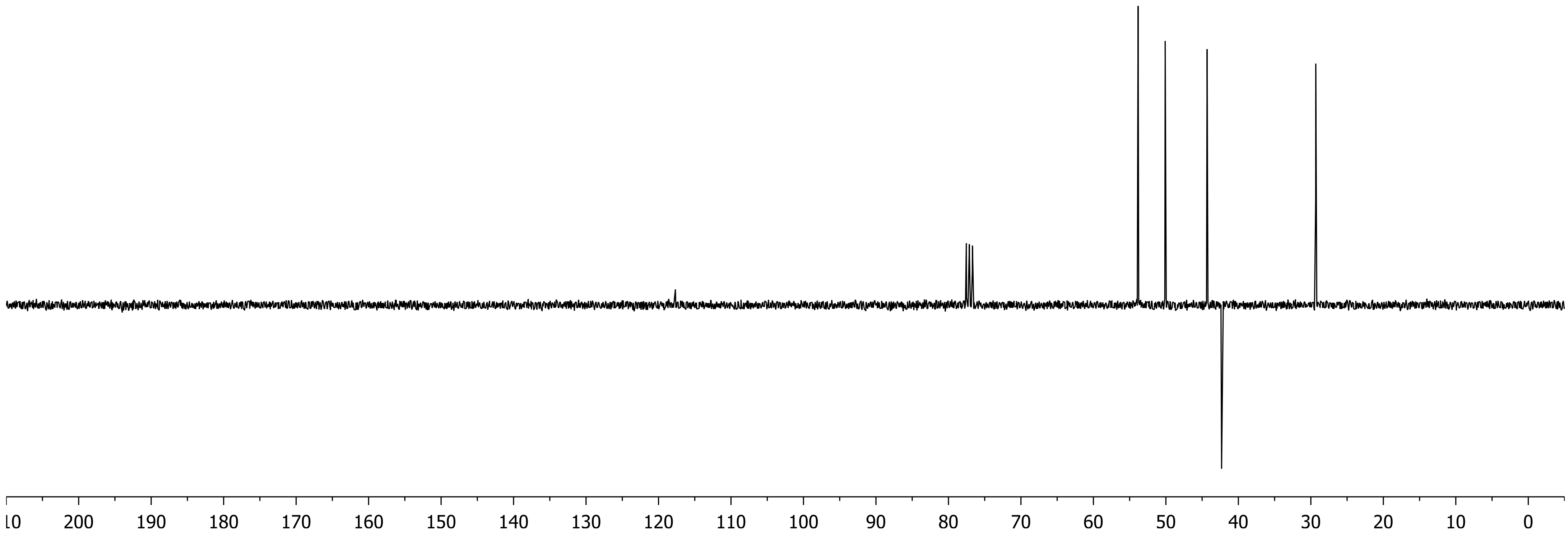

10
200
190
180
170
160
150
140
130
120
110
100
90
80
70
60
50
40
30
20
10
0

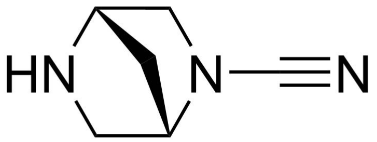

BB12

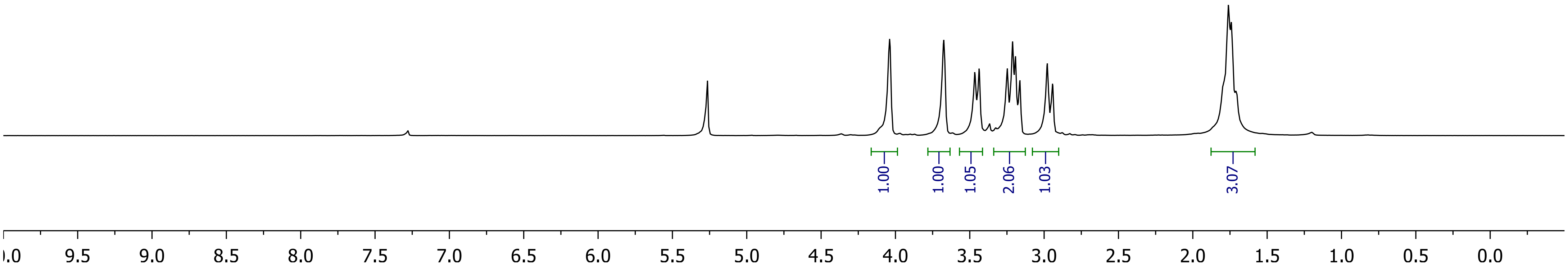

1.00
1.00
1.05
2.06
1.03
3.07
.0
9.5
9.0
8.5
8.0
7.5
7.0
6.5
6.0
5.5
5.0
4.5
4.0
3.5
3.0
2.5
2.0
1.5
1.0
0.5
0.0

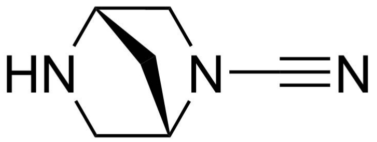
HN
N
N

BB12

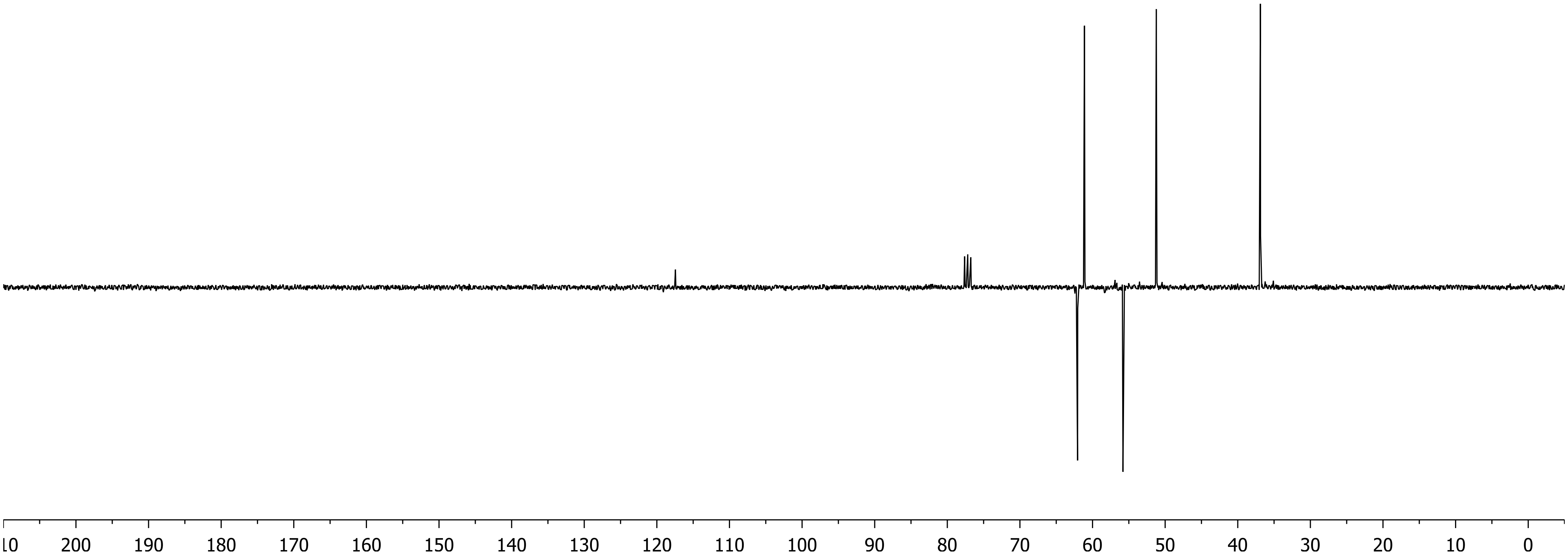
10
200
190
180
170
160
150
140
130
120
110
100
90
80
70
60
50
40
30
20
10
0

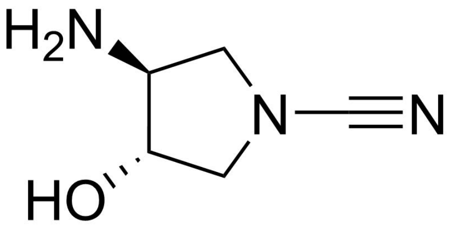
H2N
N
N
HO

BB13

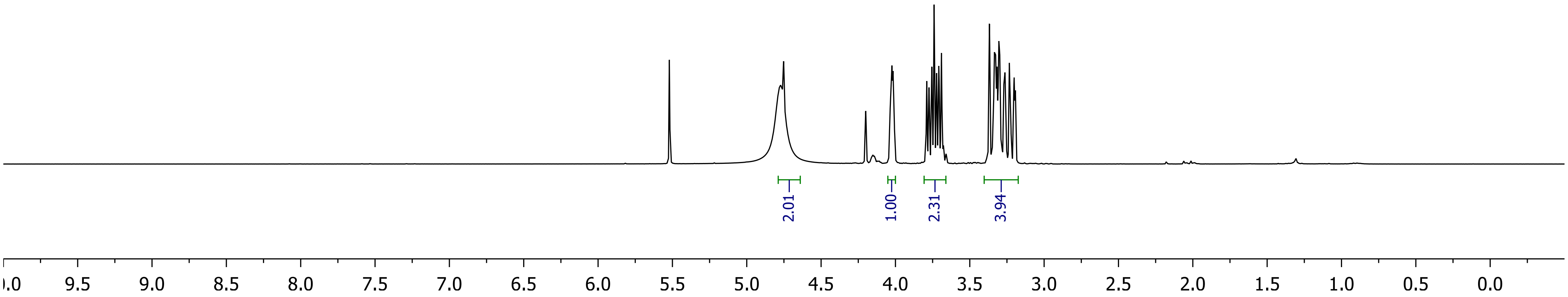
2.01
1.00
2.31
3.94
.0
9.5
9.0
8.5
8.0
7.5
7.0
6.5
6.0
5.5
5.0
4.5
4.0
3.5
3.0
2.5
2.0
1.5
1.0
0.5
0.0

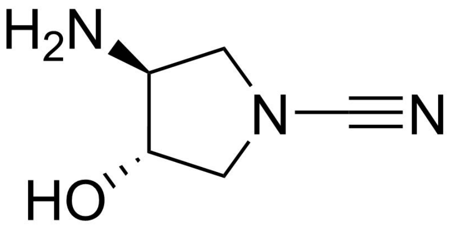
H2N
N
N
HO

BB13

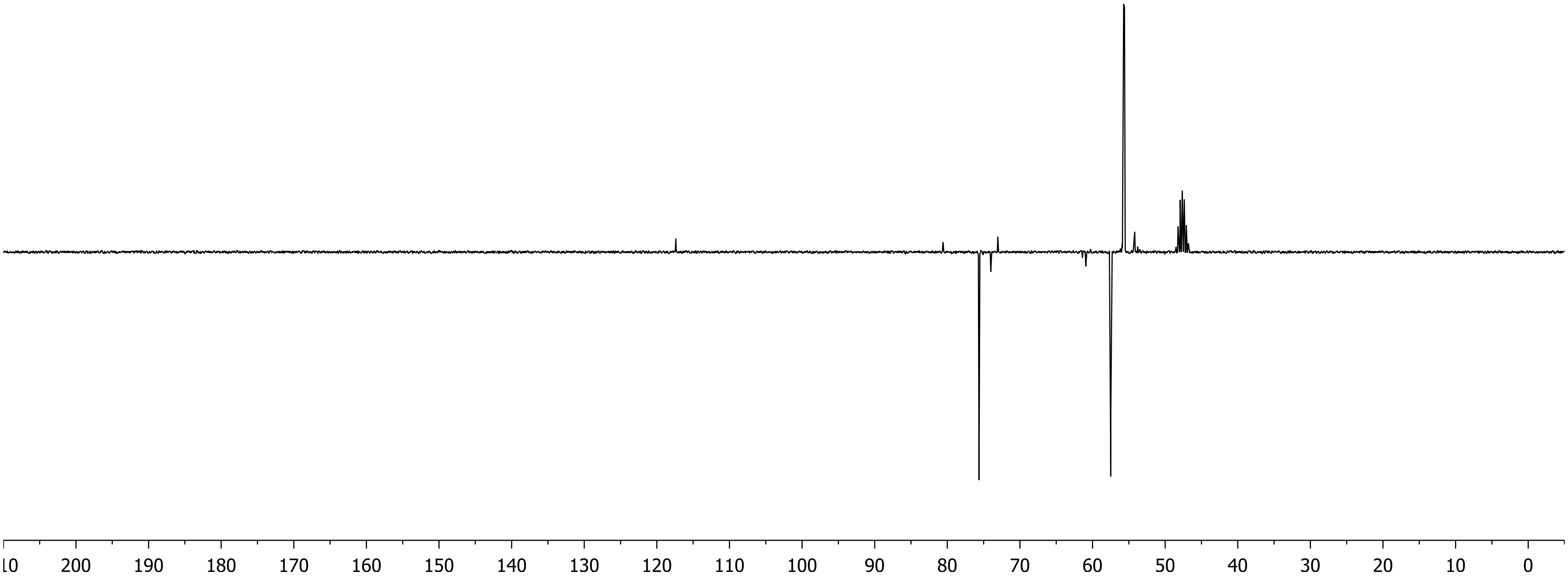
200 190 180 170 160 150 140 130 120 110 100 90 80 70 60 50 40 30 20 10 0

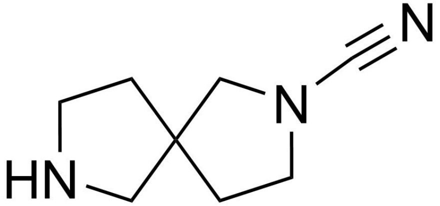

BB14

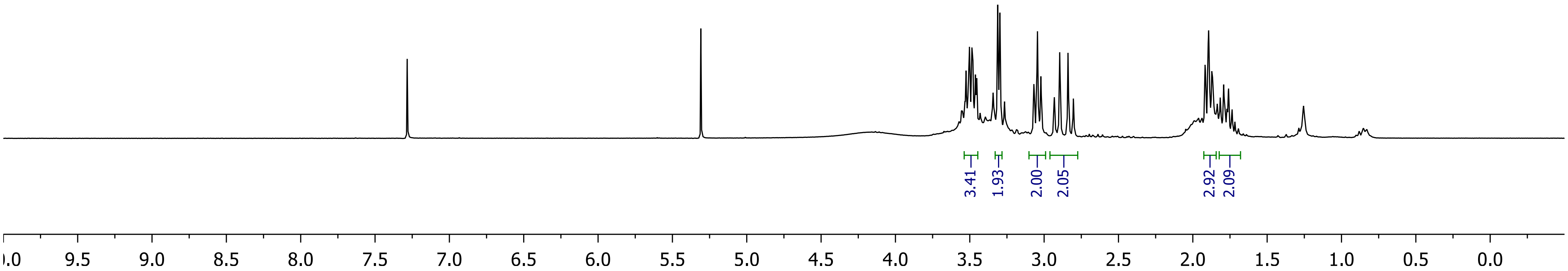
3.41
1.93
2.00
2.05
2.92
2.09
9.5 9.0 8.5 8.0 7.5 7.0 6.5 6.0 5.5 5.0 4.5 4.0 3.5 3.0 2.5 2.0 1.5 1.0 0.5 0.0

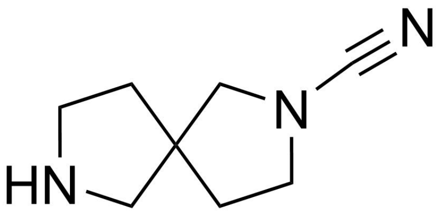

BB14

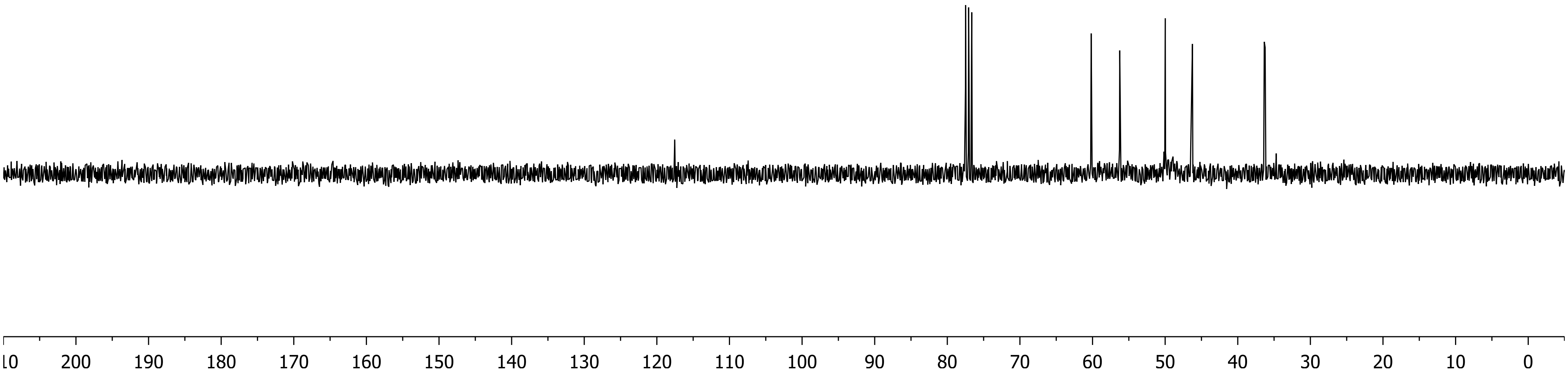

10
200
190
180
170
160
150
140
130
120
110
100
90
80
70
60
50
40
30
20
10
0

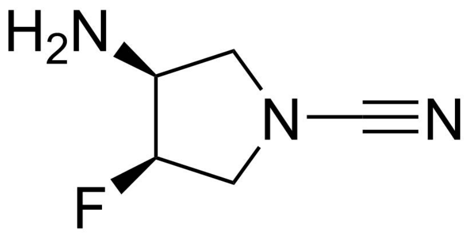

BB15

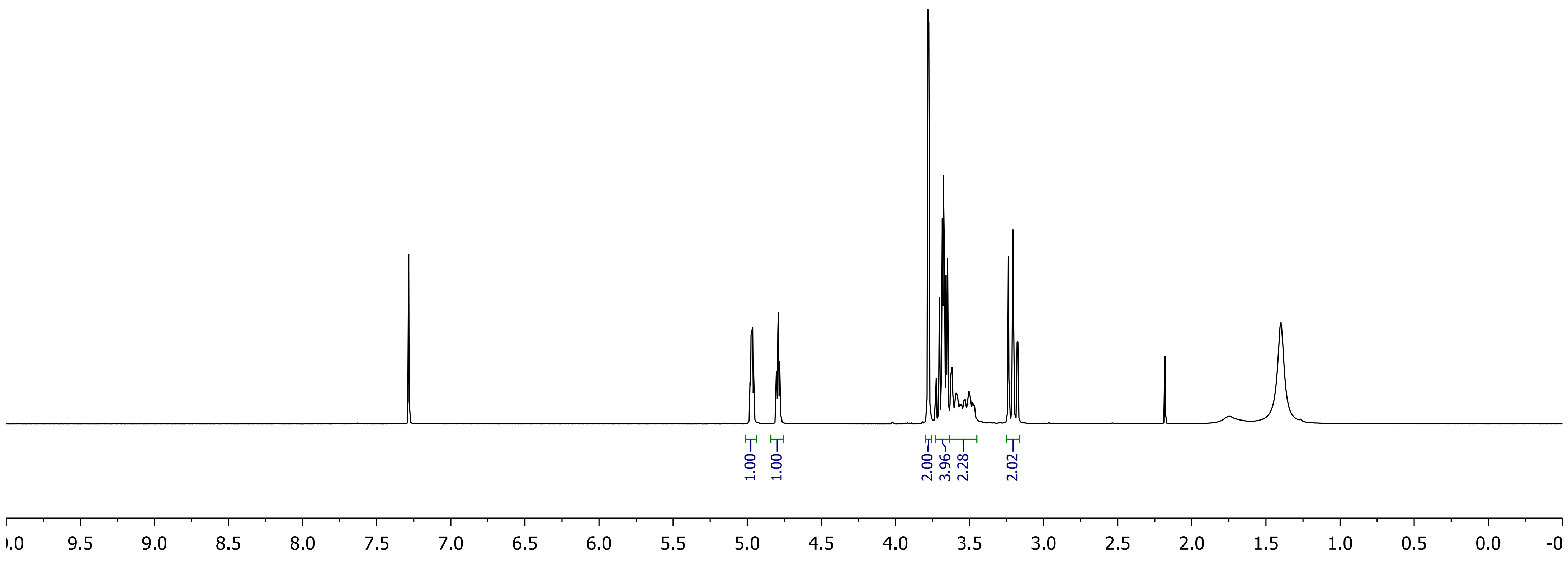

1.00
1.00
2.00
3.96
2.28
2.02
0.0
9.5
9.0
8.5
8.0
7.5
7.0
6.5
6.0
5.5
5.0
4.5
4.0
3.5
3.0
2.5
2.0
1.5
1.0
0.5
0.0

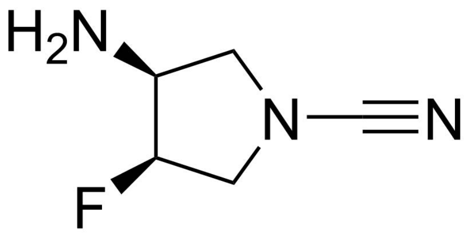
H2N
N
N
F

BB15

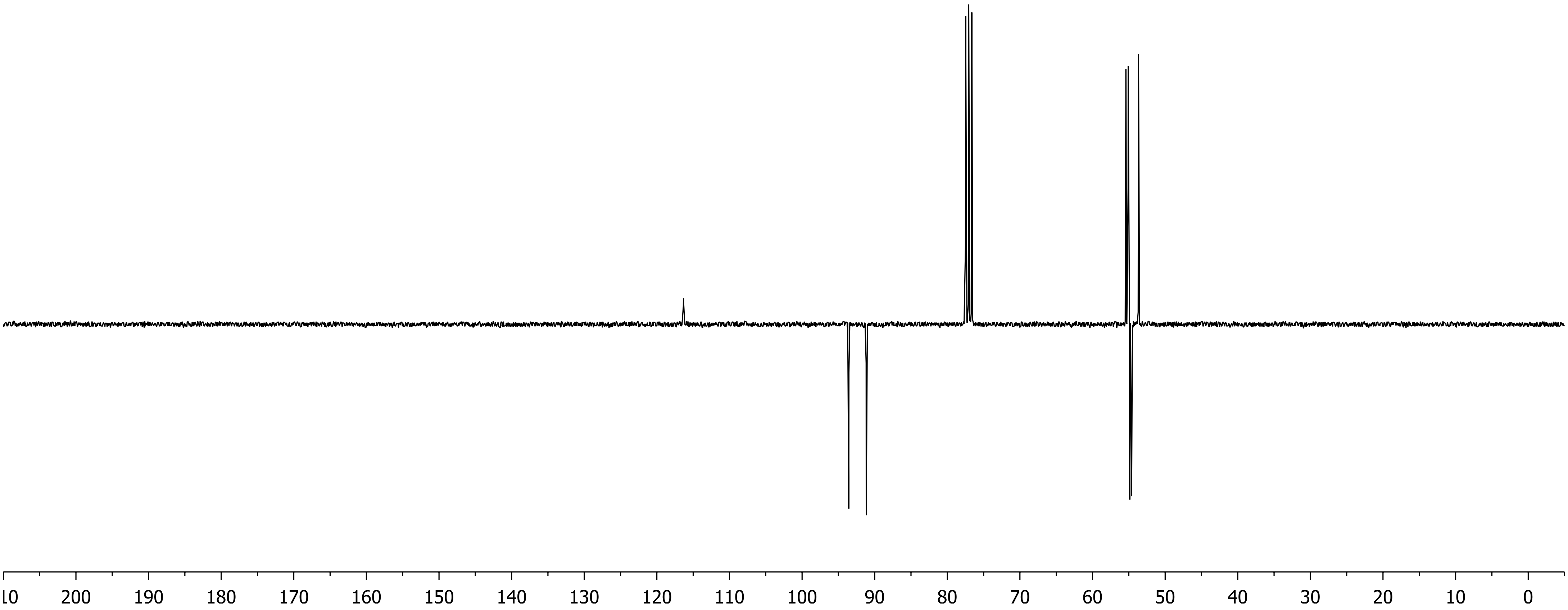
10 200 190 180 170 160 150 140 130 120 110 100 90 80 70 60 50 40 30 20 10 0

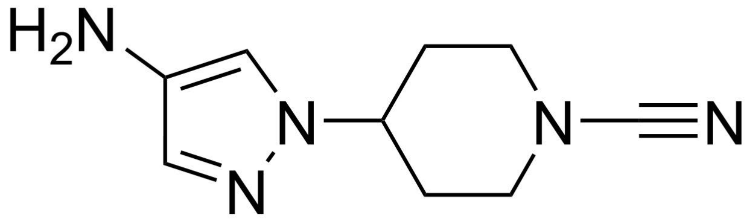

BB16

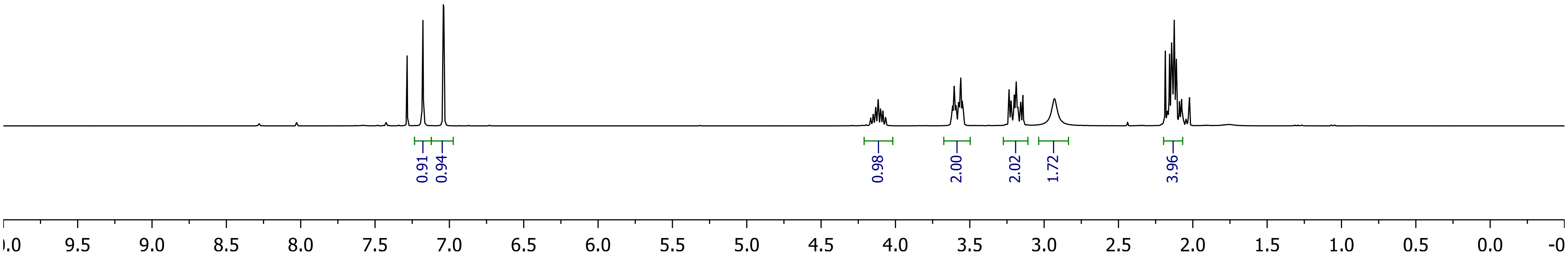
0.91
0.94
0.98
2.00
2.02
1.72
3.96
9.5 9.0 8.5 8.0 7.5 7.0 6.5 6.0 5.5 5.0 4.5 4.0 3.5 3.0 2.5 2.0 1.5 1.0 0.5 0.0

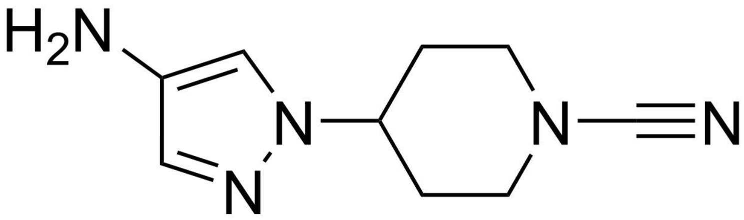

BB16

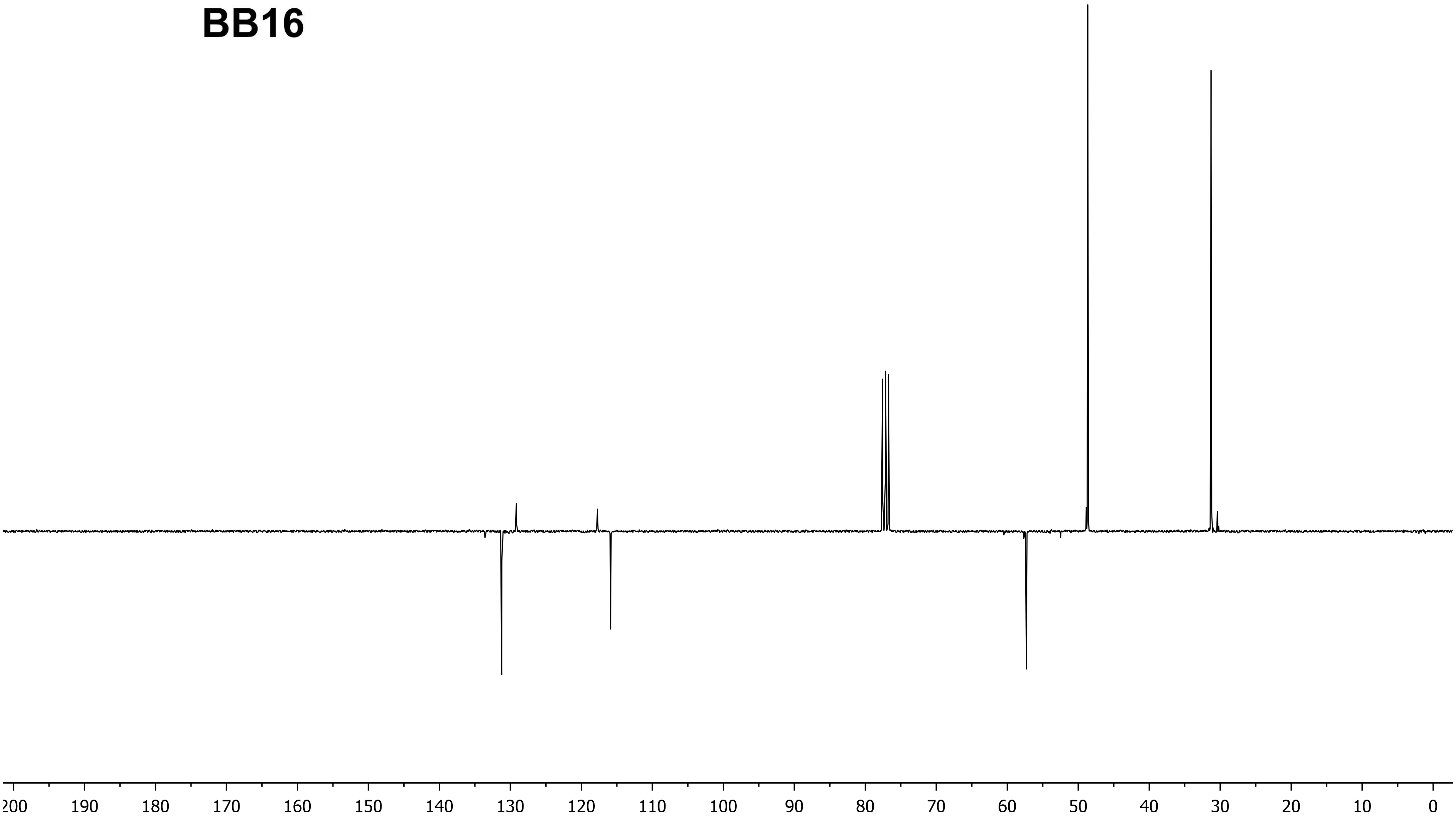

200
190
180
170
160
150
140
130
120
110
100
90
80
70
60
50
40
30
20
10
0

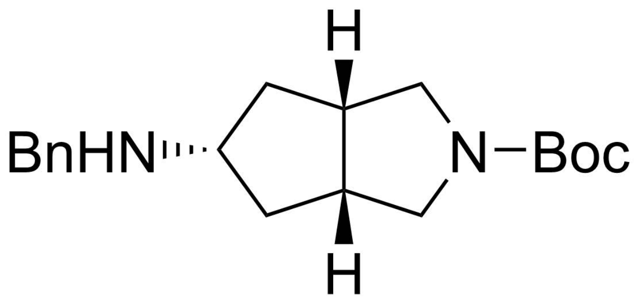
H
BnHN
N−Boc
H

6

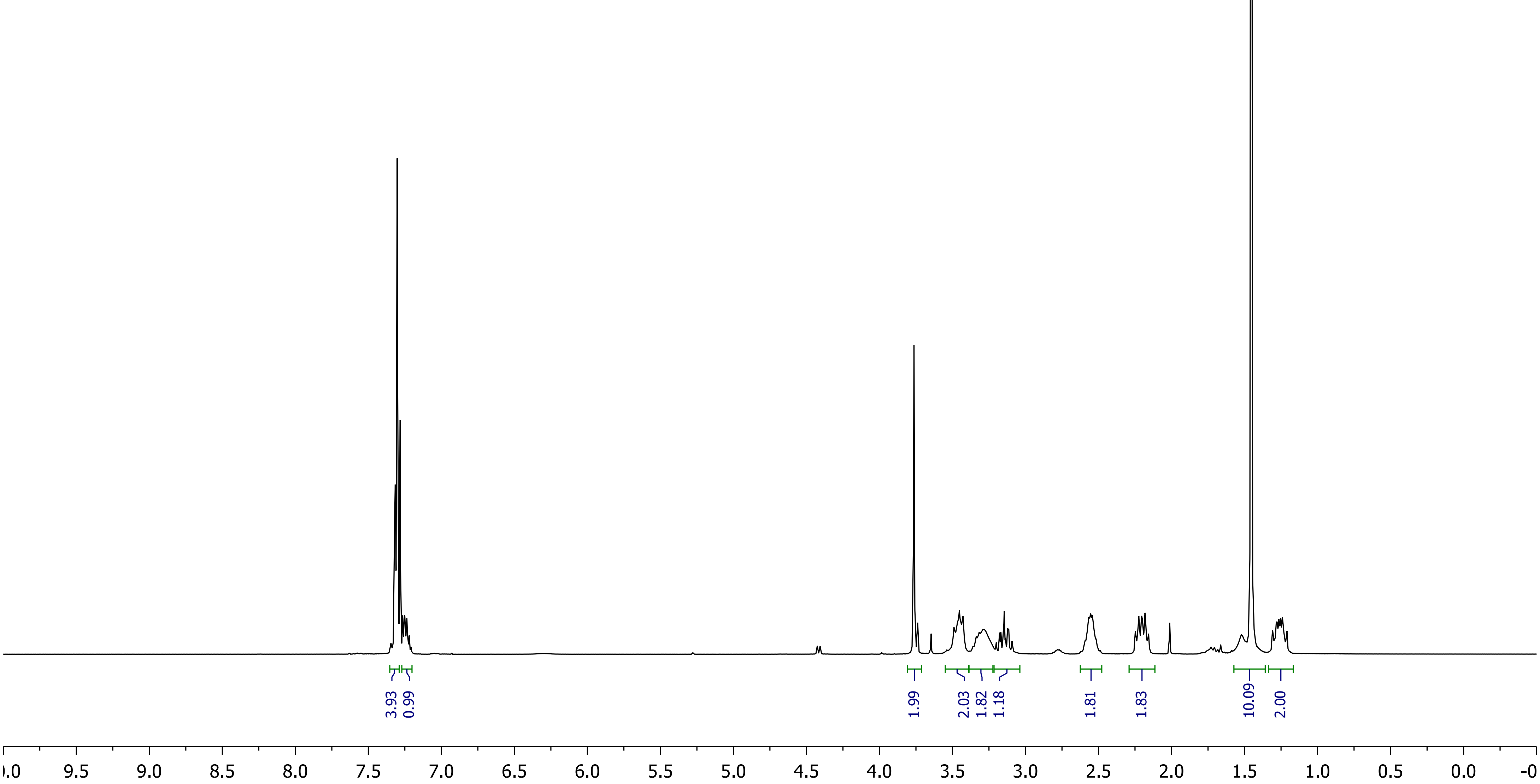
3.93
0.99
1.99
2.03
1.82
1.18
1.81
1.83
10.09
2.00
9.5
9.0
8.5
8.0
7.5
7.0
6.5
6.0
5.5
5.0
4.5
4.0
3.5
3.0
2.5
2.0
1.5
1.0
0.5
0.0

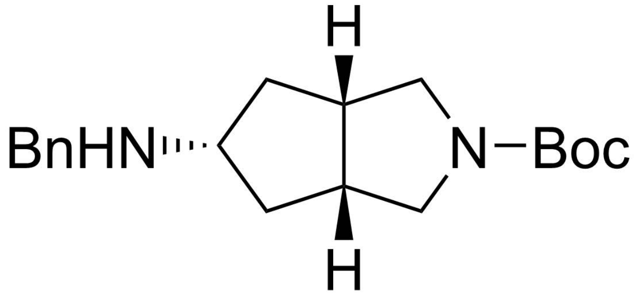
H
BnHN
N—Boc
H

6

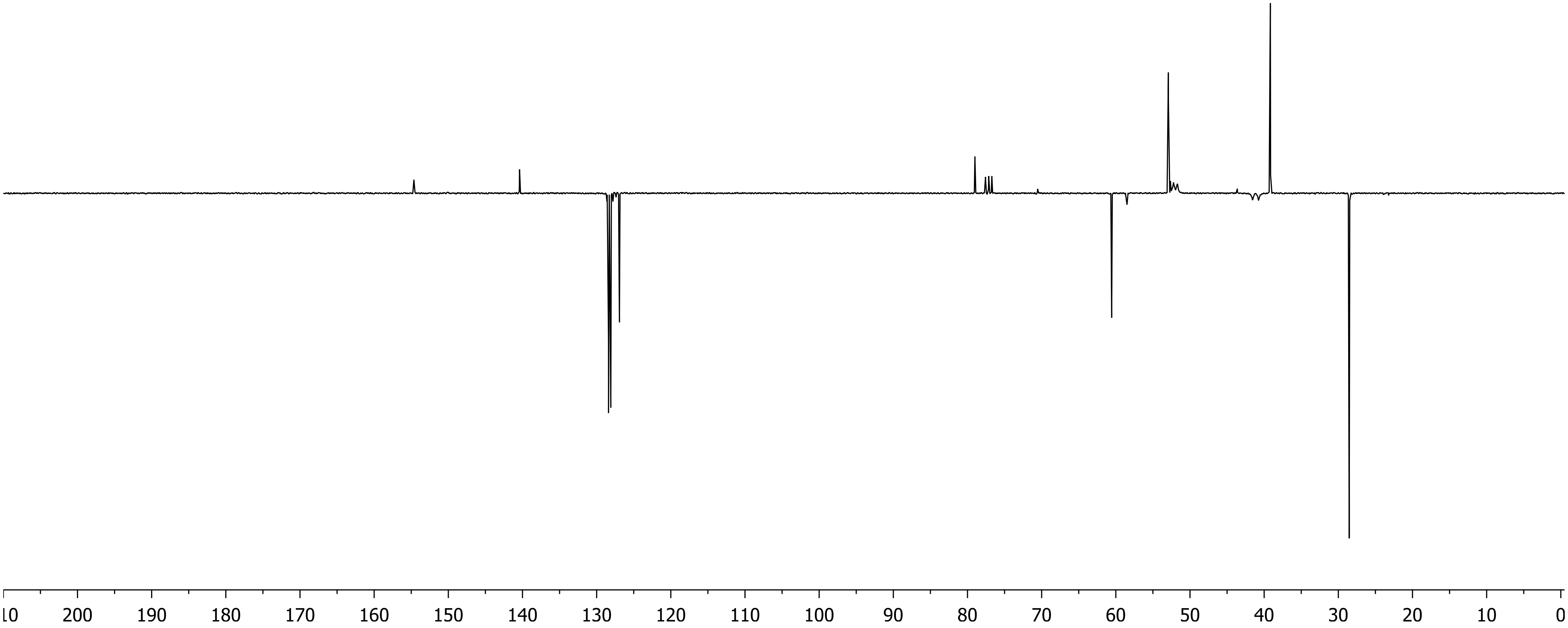
200
190
180
170
160
150
140
130
120
110
100
90
80
70
60
50
40
30
20
10
0

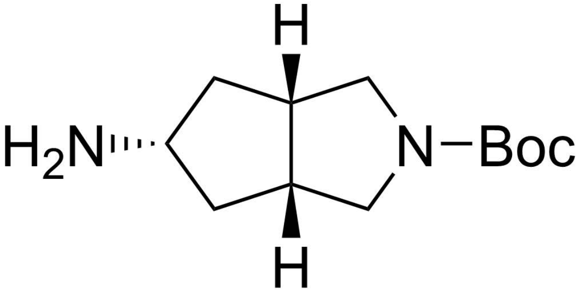
H
H
H_2N
N
Boc
H

7

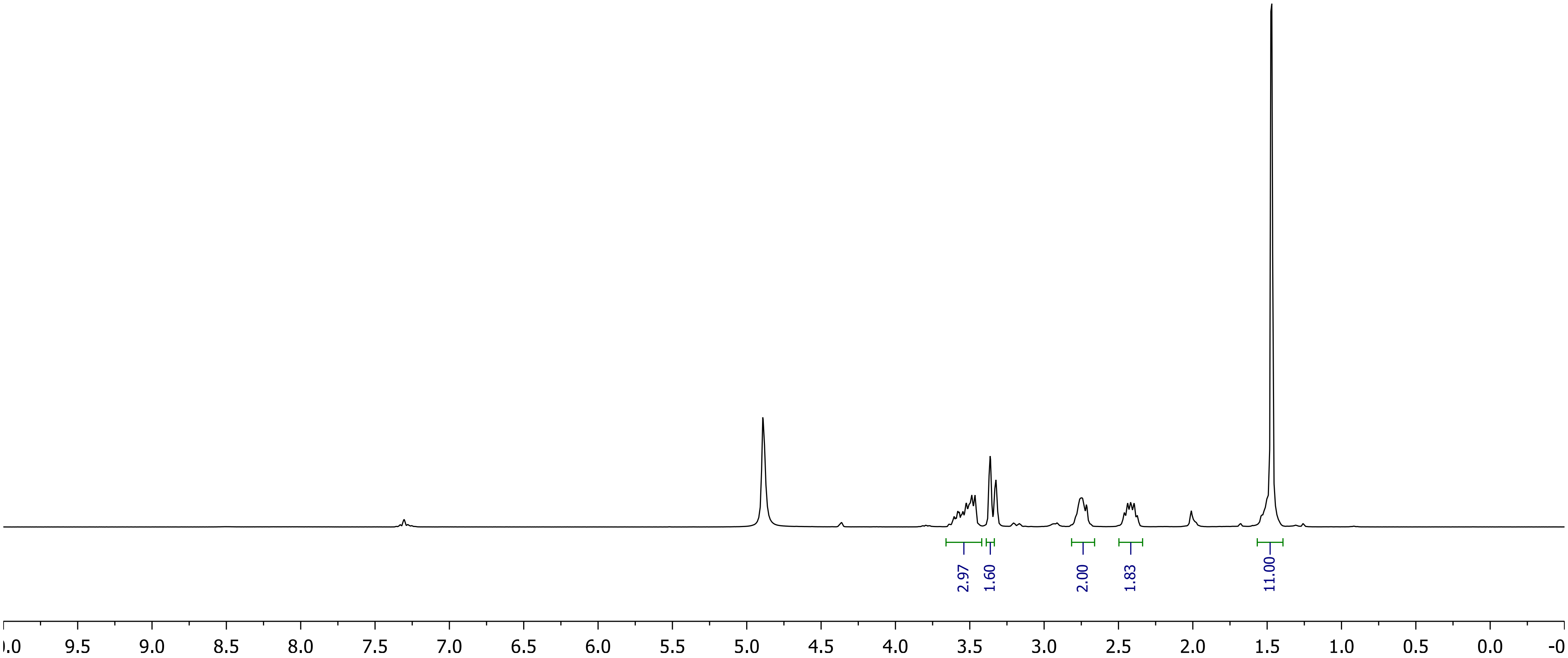
2.97
1.60
2.00
1.83
11.00
.0
9.5
9.0
8.5
8.0
7.5
7.0
6.5
6.0
5.5
5.0
4.5
4.0
3.5
3.0
2.5
2.0
1.5
1.0
0.5
0.0
-0

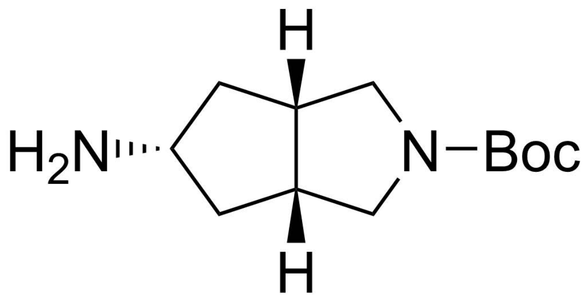
H
H2N
N
Boc
H

7

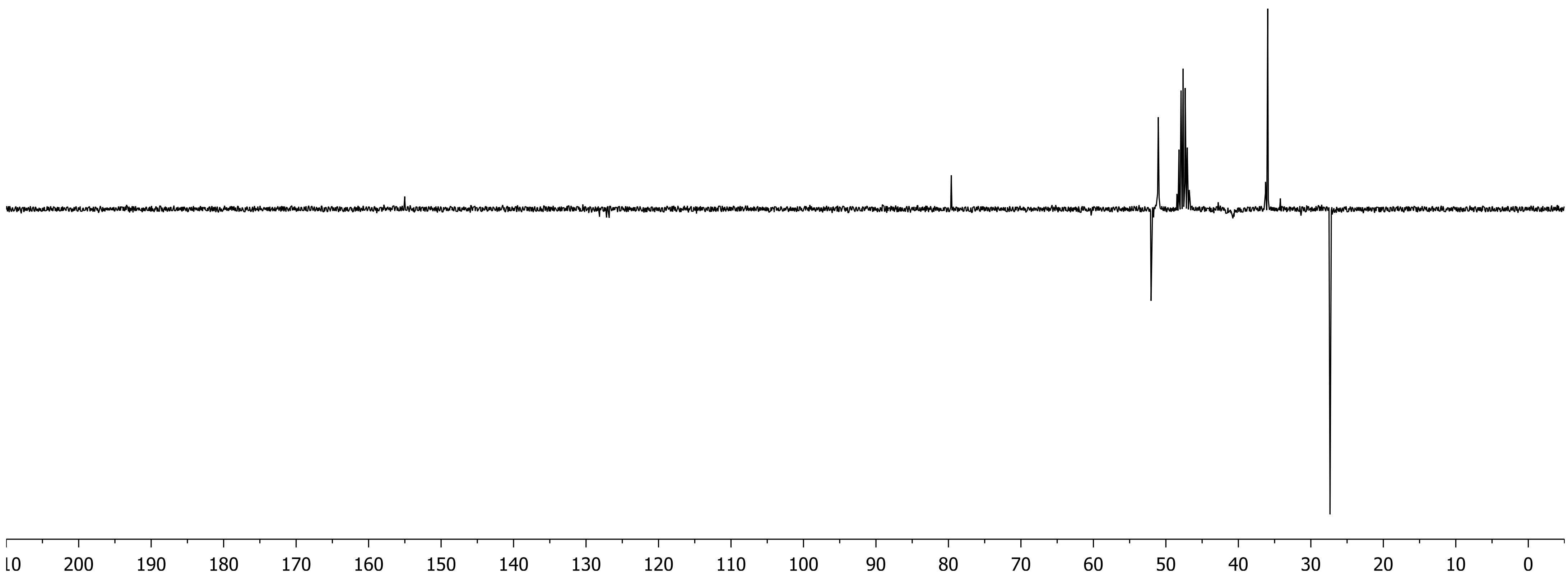
10 200 190 180 170 160 150 140 130 120 110 100 90 80 70 60 50 40 30 20 10 0

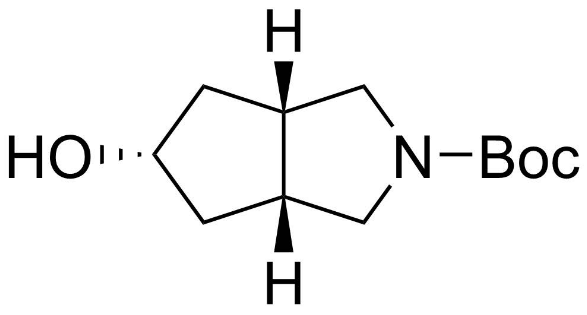

8

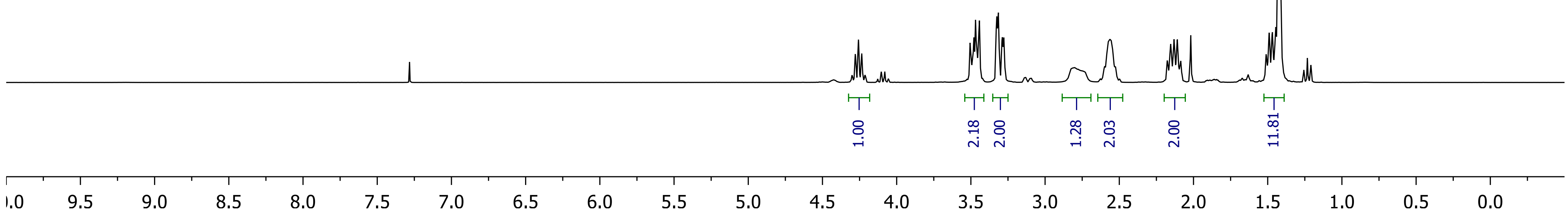
1.00
2.18
2.00
1.28
2.03
2.00
11.81
.0
9.5
9.0
8.5
8.0
7.5
7.0
6.5
6.0
5.5
5.0
4.5
4.0
3.5
3.0
2.5
2.0
1.5
1.0
0.5
0.0

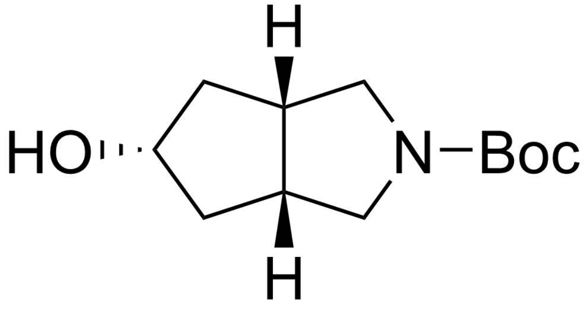
H
HO
N
Boc
H

8

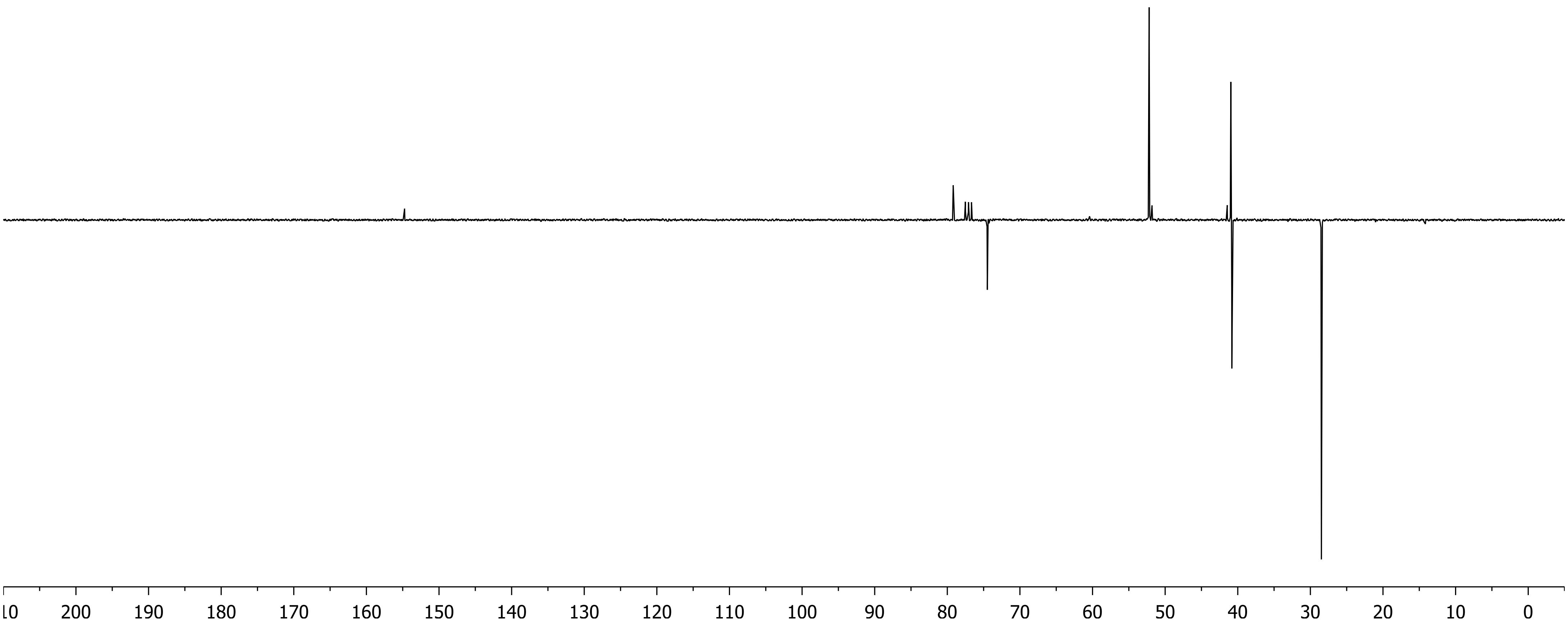
10 200 190 180 170 160 150 140 130 120 110 100 90 80 70 60 50 40 30 20 10 0

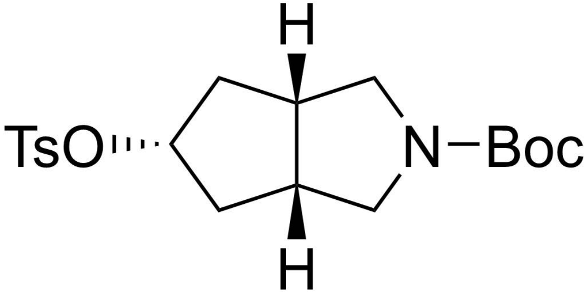
H
TsO
N—Boc
H

9

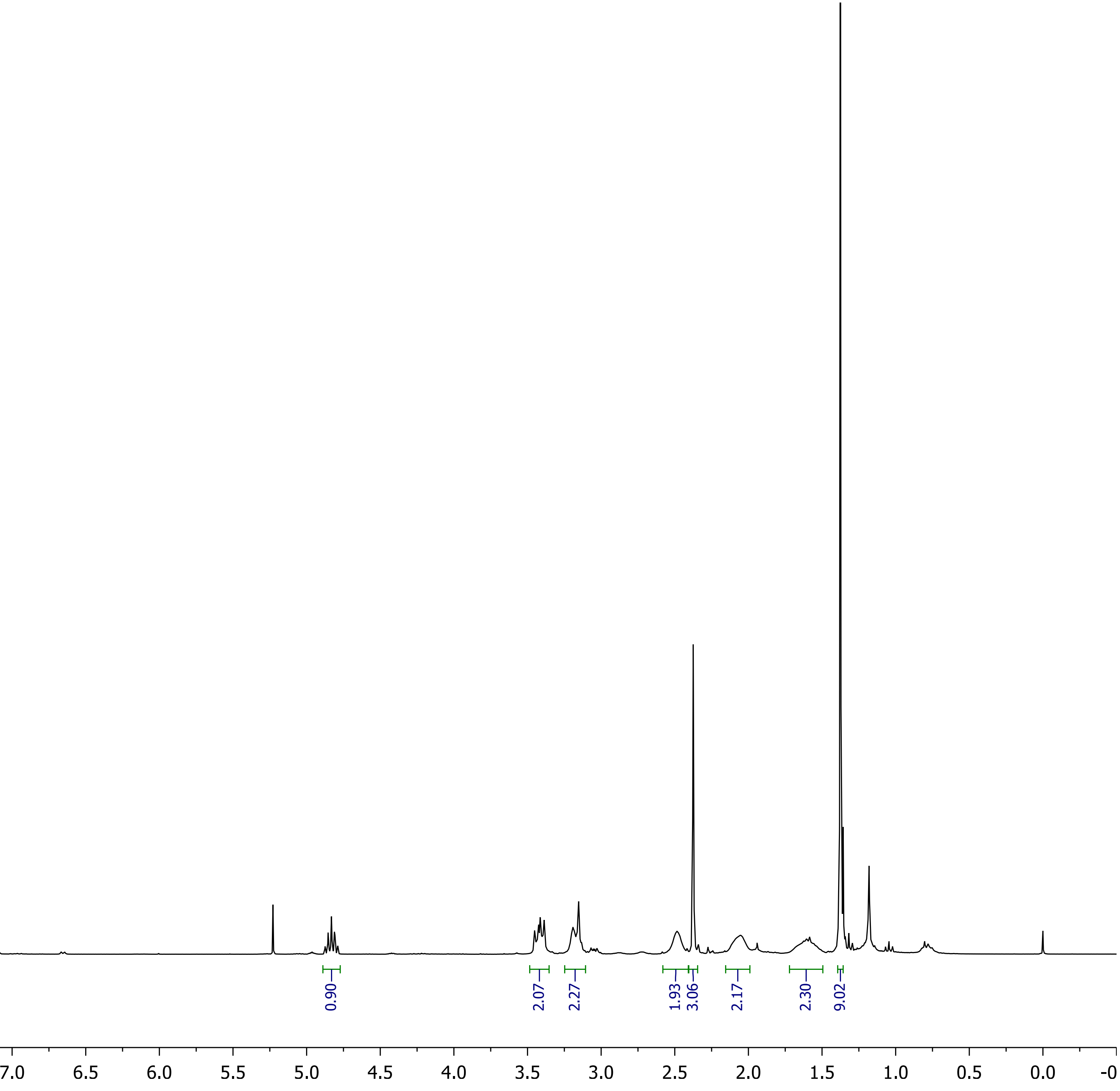
2.00
2.05
0.90
2.07
2.27
1.93
3.06
2.17
2.30
9.02
).0 9.5 9.0 8.5 8.0 7.5 7.0 6.5 6.0 5.5 5.0 4.5 4.0 3.5 3.0 2.5 2.0 1.5 1.0 0.5 0.0 -0

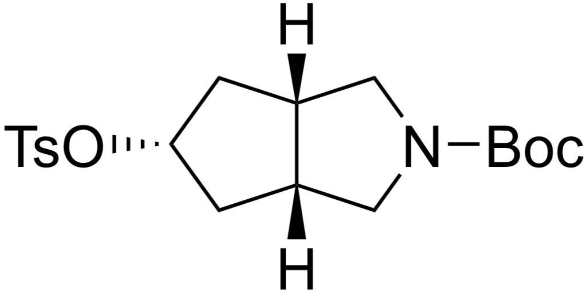
H
TsO
N—Boc
H

9

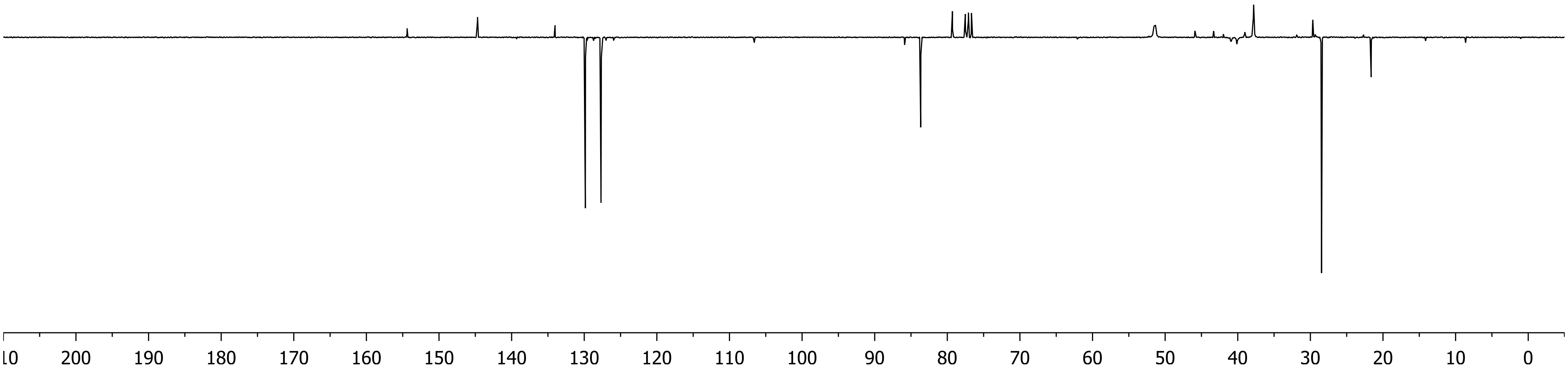
200 190 180 170 160 150 140 130 120 110 100 90 80 70 60 50 40 30 20 10 0

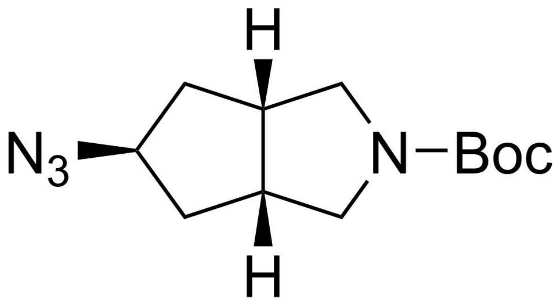
H
N3
N
Boc
H

10

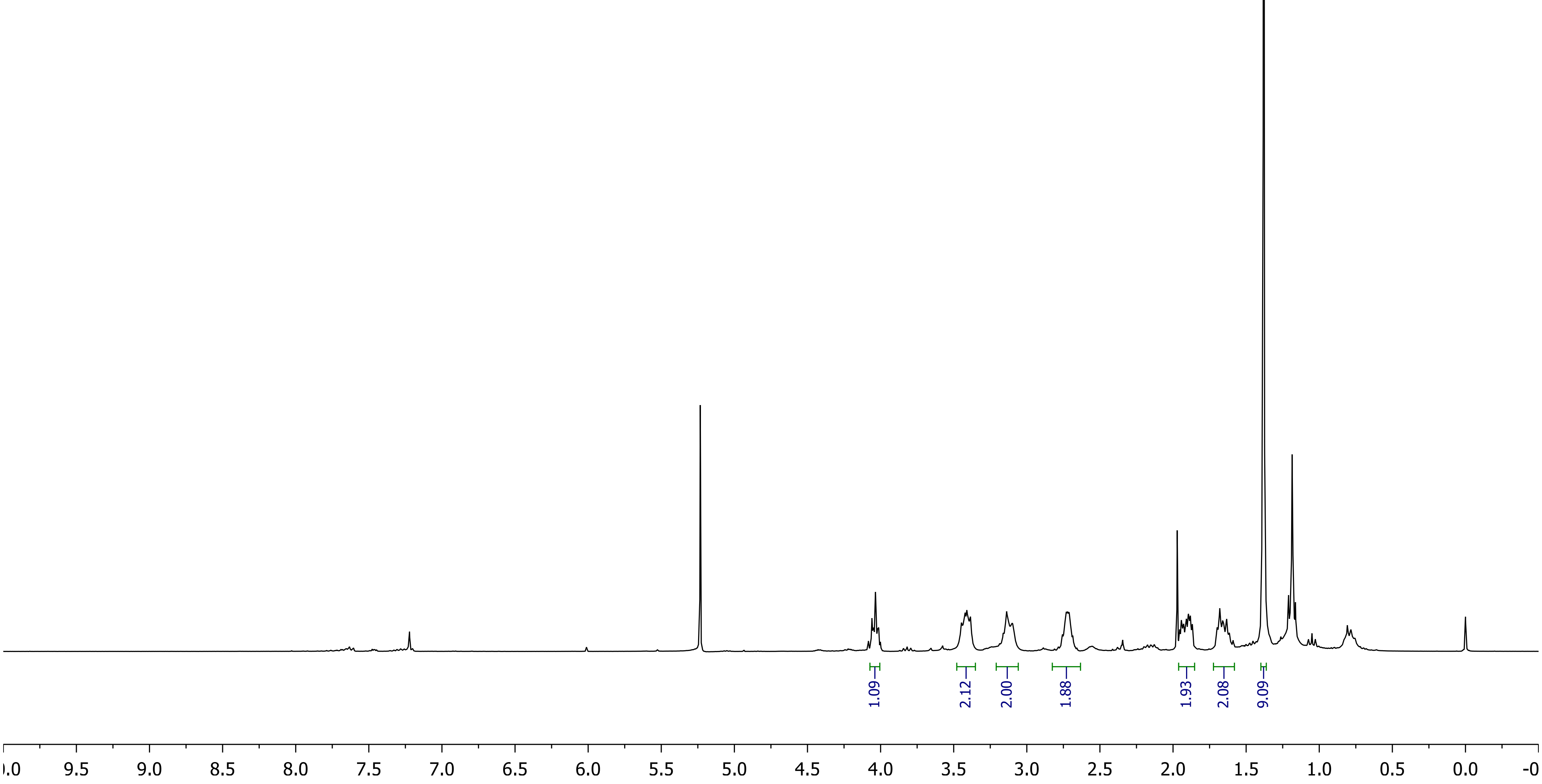
1.09
2.12
2.00
1.88
1.93
2.08
9.09
9.5
9.0
8.5
8.0
7.5
7.0
6.5
6.0
5.5
5.0
4.5
4.0
3.5
3.0
2.5
2.0
1.5
1.0
0.5
0.0

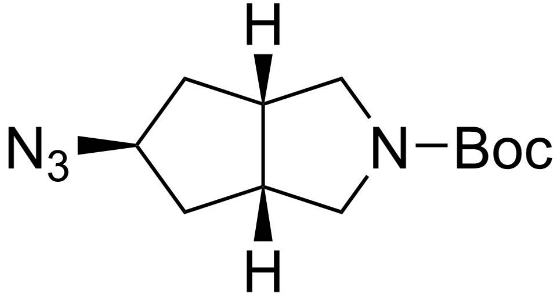
H
N_3
N
Boc
H

10

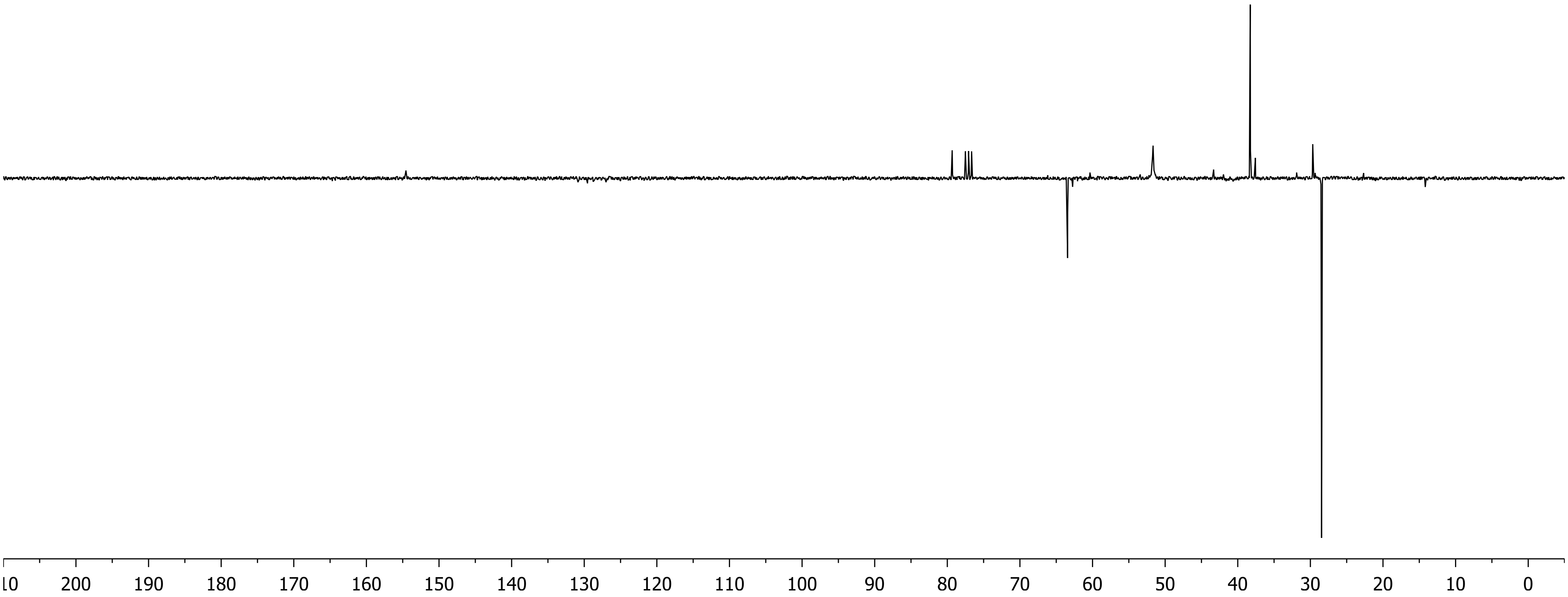
10
200
190
180
170
160
150
140
130
120
110
100
90
80
70
60
50
40
30
20
10
0

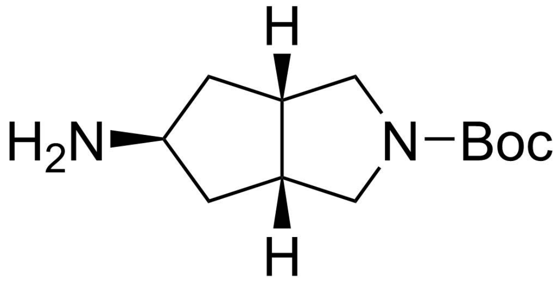
H
H
H2N
N
Boc

11

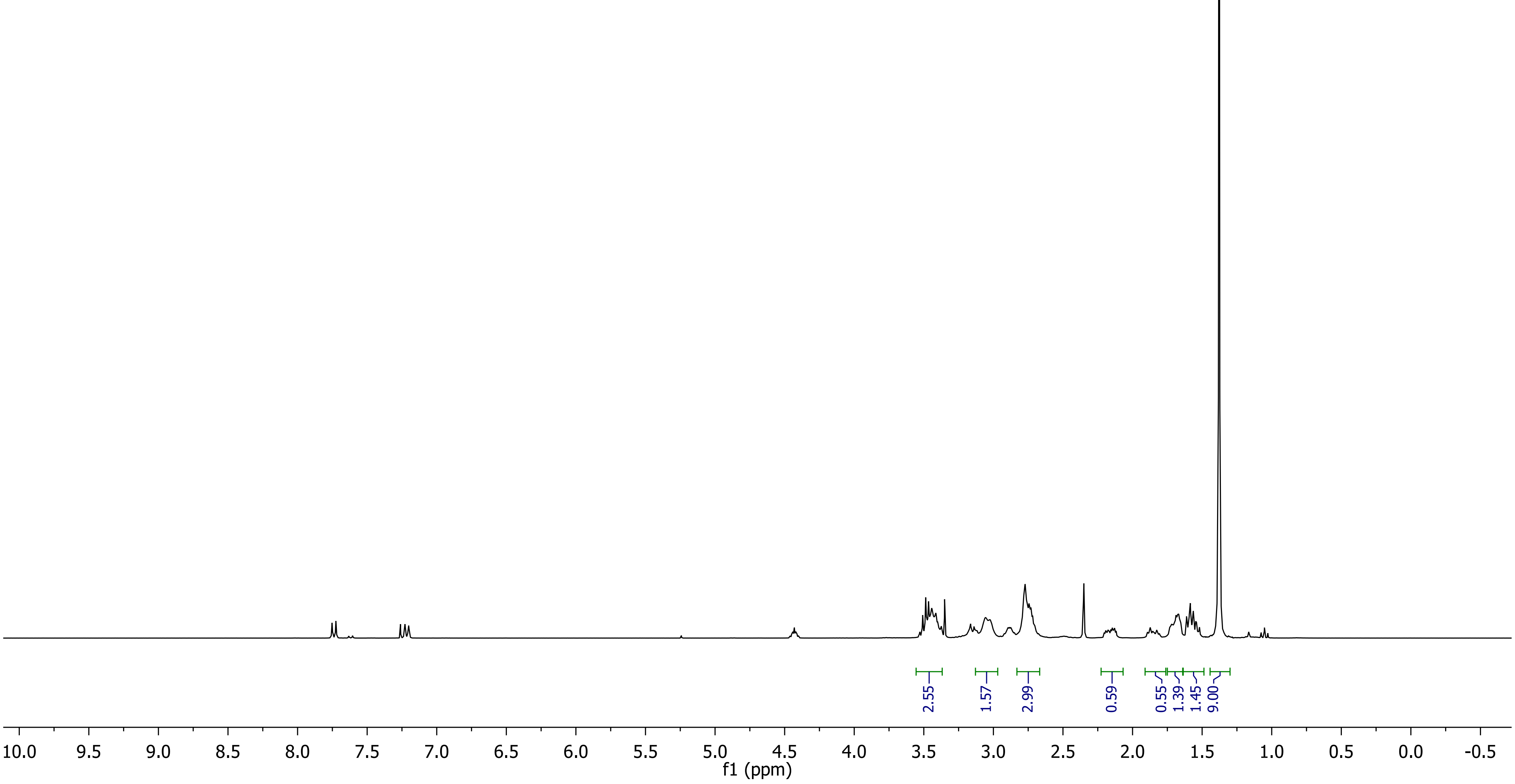
2.55
1.57
2.99
0.59
0.55
1.39
1.45
9.00
10.0
9.5
9.0
8.5
8.0
7.5
7.0
6.5
6.0
5.5
5.0
4.5
4.0
3.5
3.0
2.5
2.0
1.5
1.0
0.5
0.0
-0.5
f1 (ppm)

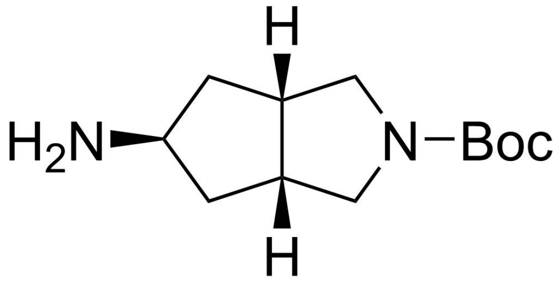
H
H₂N
N
Boc
H

11

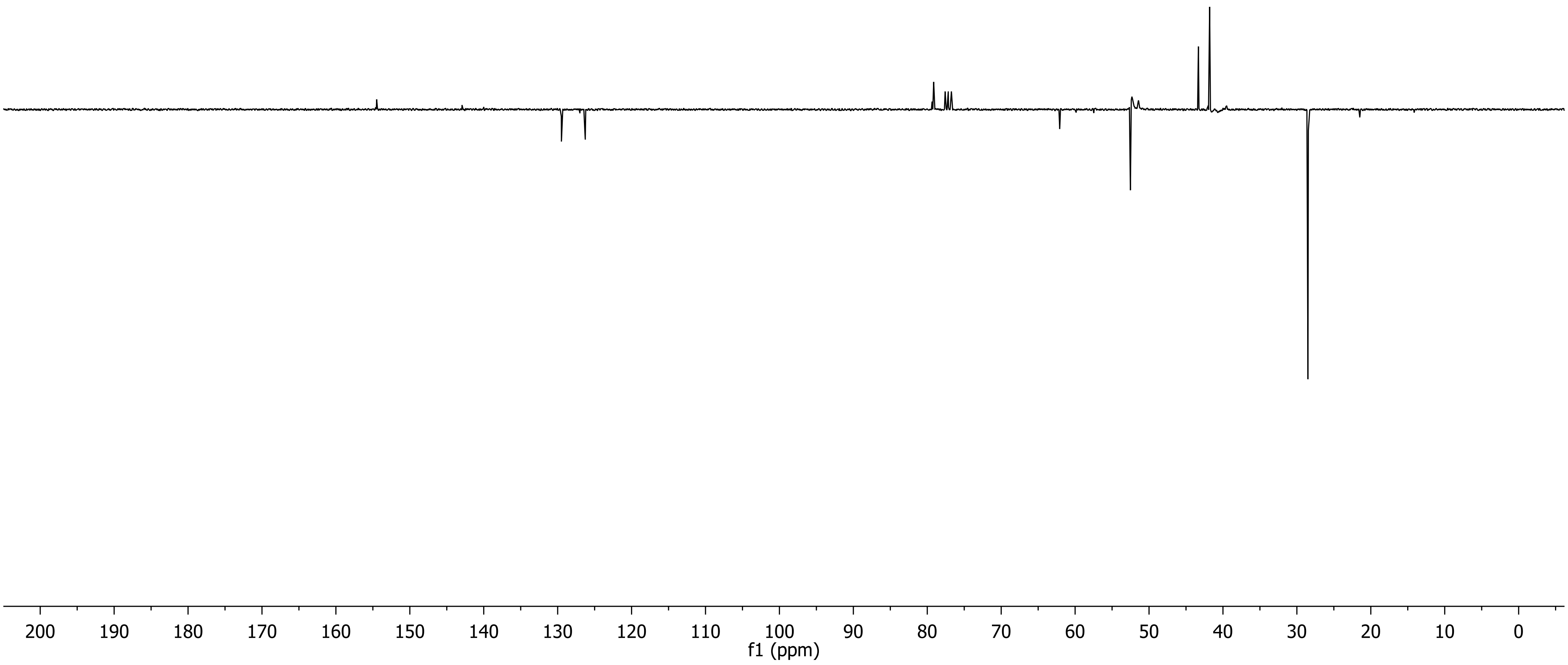
200
190
180
170
160
150
140
130
120
110
100
90
80
70
60
50
40
30
20
10
0
f1 (ppm)

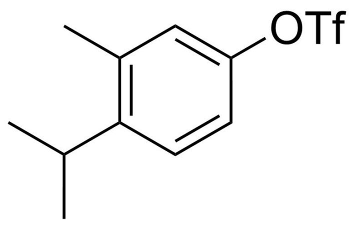
OTf

13

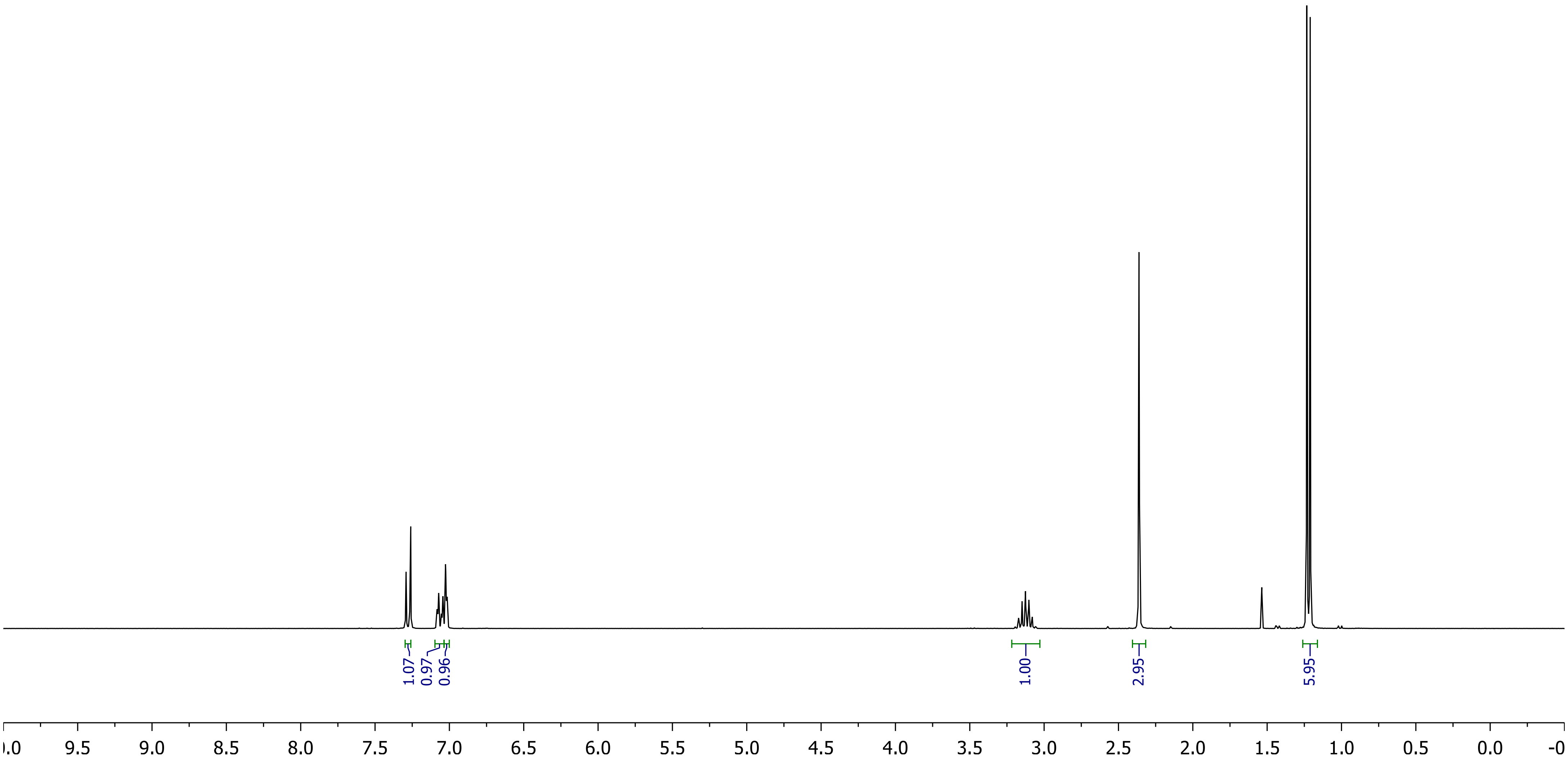
1.07
0.97
0.96
1.00
2.95
5.95
9.5
9.0
8.5
8.0
7.5
7.0
6.5
6.0
5.5
5.0
4.5
4.0
3.5
3.0
2.5
2.0
1.5
1.0
0.5
0.0

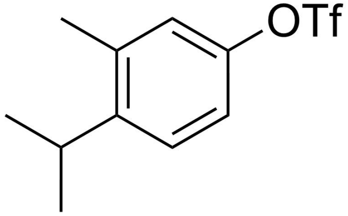
OTf

13

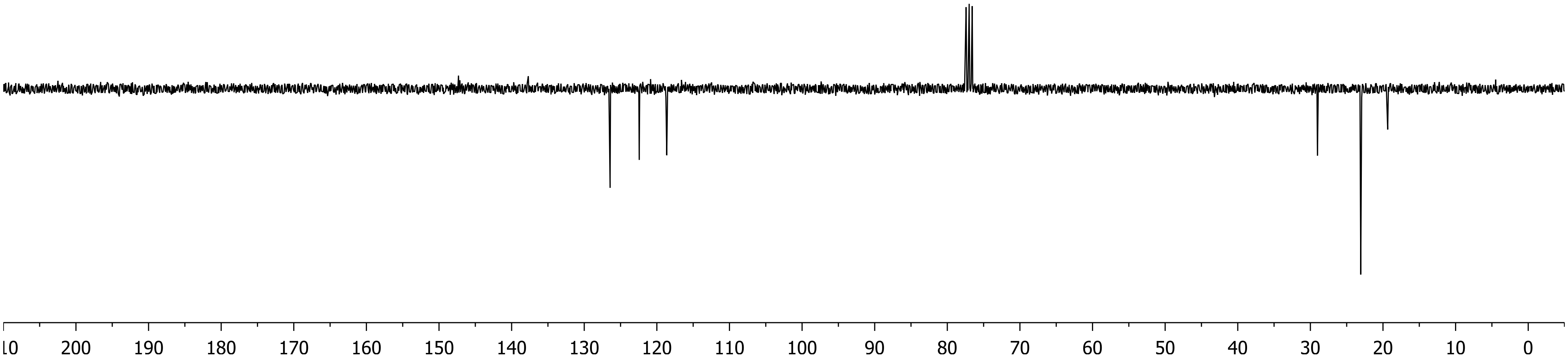
10
200
190
180
170
160
150
140
130
120
110
100
90
80
70
60
50
40
30
20
10
0

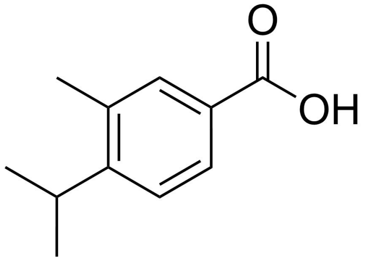
O
OH

14

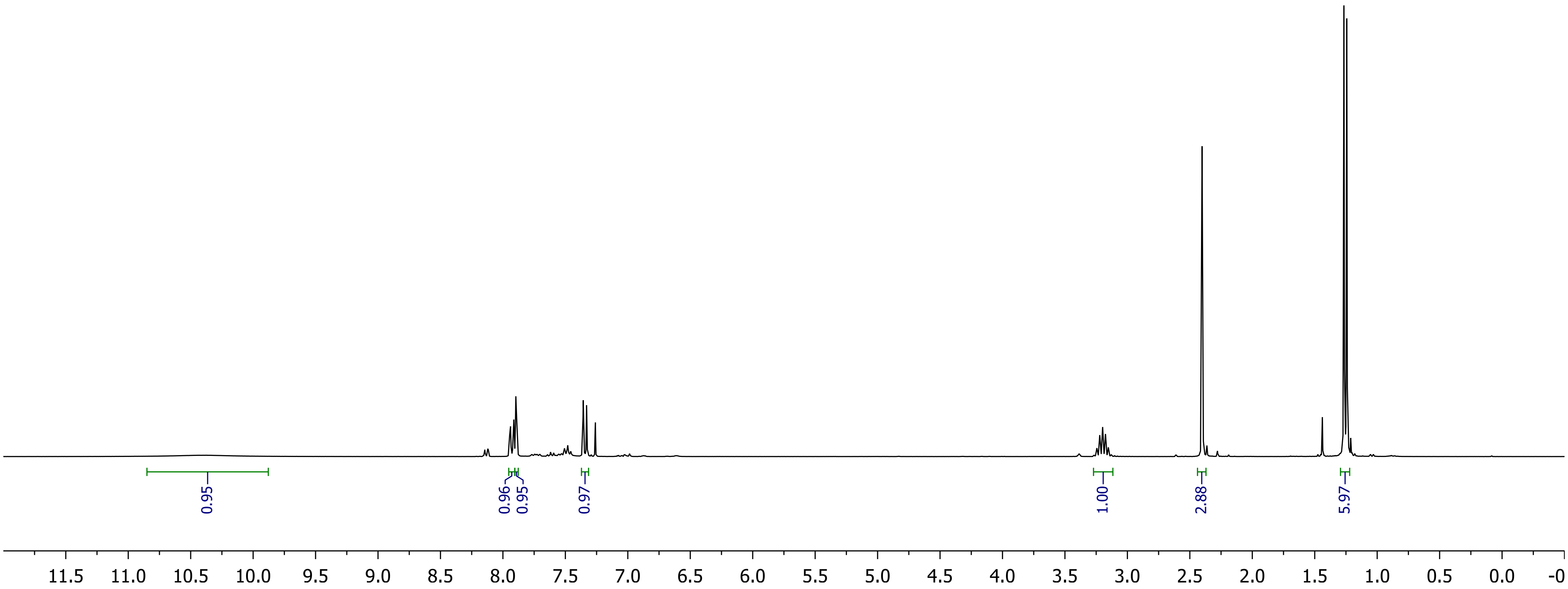
0.95
0.96
0.95
0.97
1.00
2.88
5.97
11.5 11.0 10.5 10.0 9.5 9.0 8.5 8.0 7.5 7.0 6.5 6.0 5.5 5.0 4.5 4.0 3.5 3.0 2.5 2.0 1.5 1.0 0.5 0.0 -0

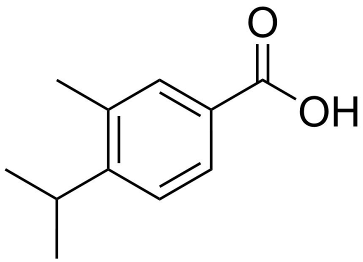
O
OH

14

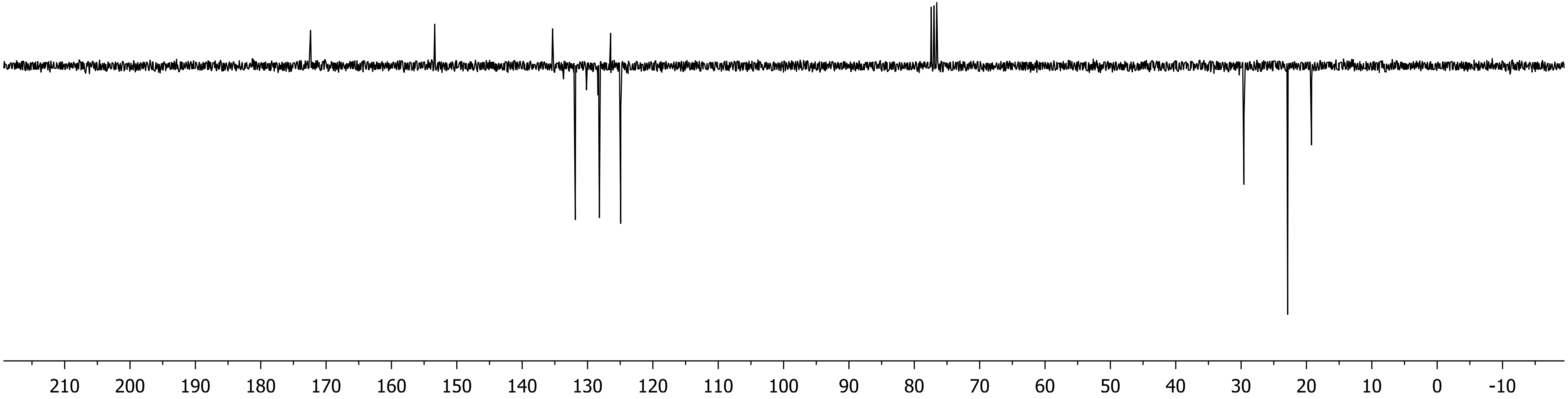
210 200 190 180 170 160 150 140 130 120 110 100 90 80 70 60 50 40 30 20 10 0 -10

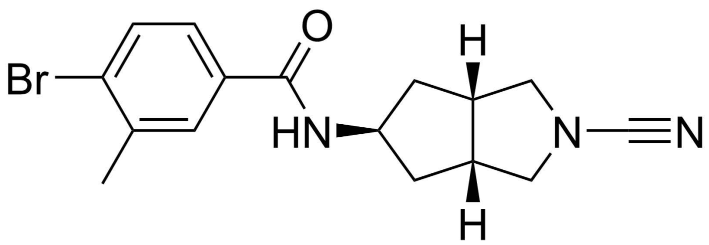

BB07CA28

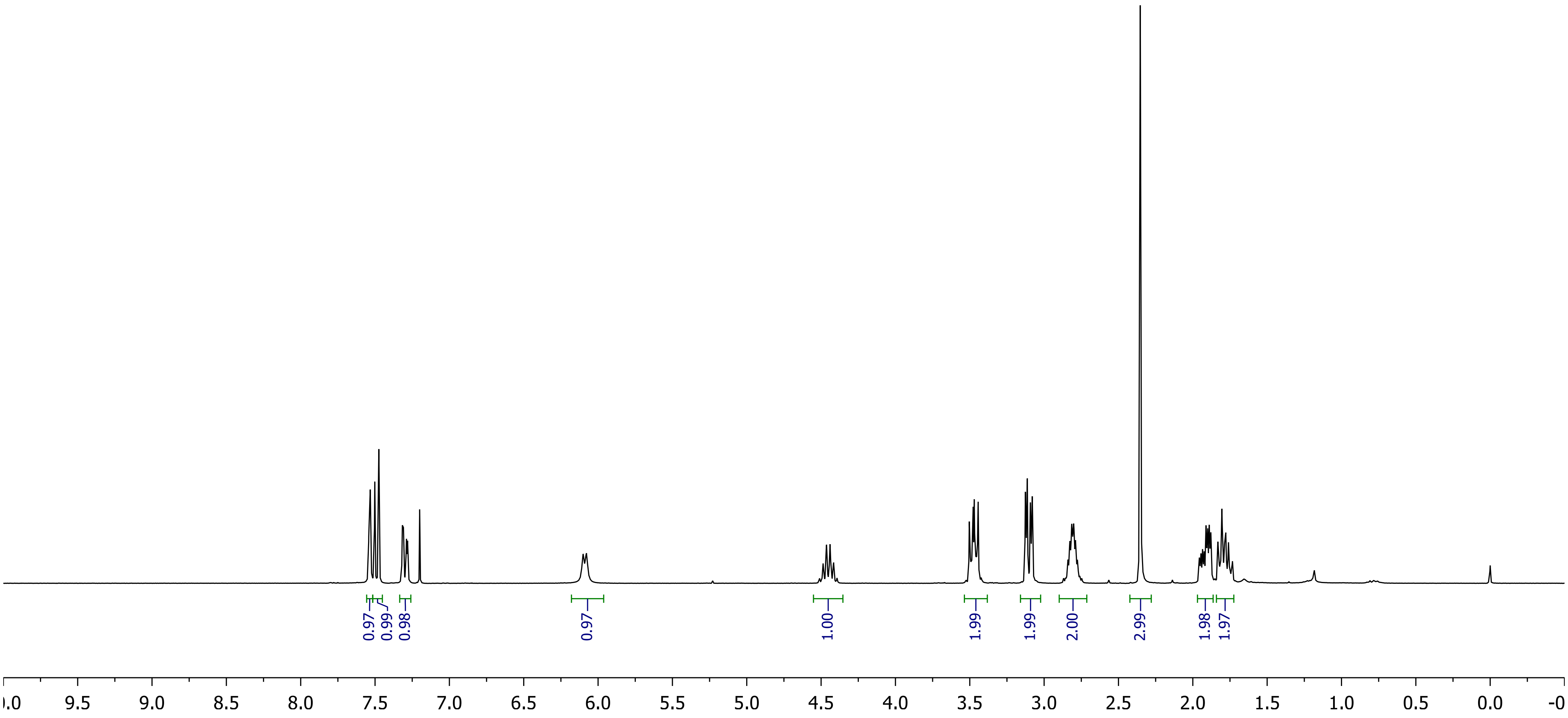
0.97
0.99
0.98
0.97
1.00
1.99
1.99
2.00
2.99
1.98
1.97
9.5
9.0
8.5
8.0
7.5
7.0
6.5
6.0
5.5
5.0
4.5
4.0
3.5
3.0
2.5
2.0
1.5
1.0
0.5
0.0

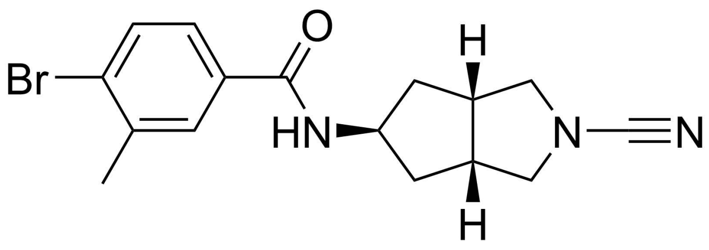

BB07CA28

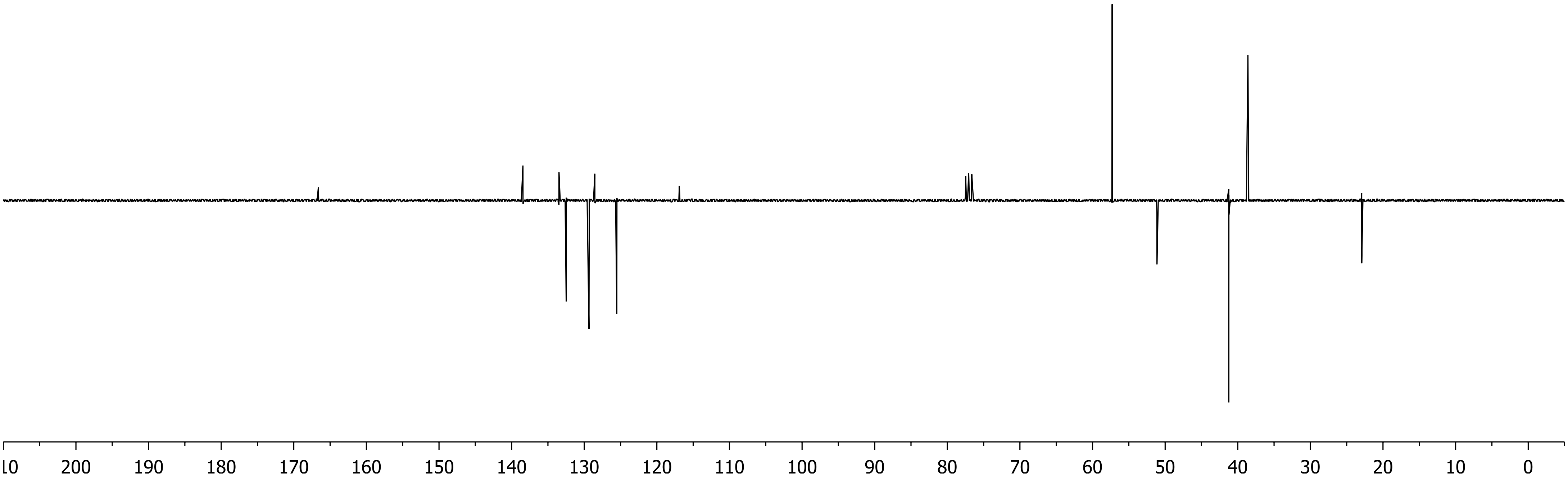

10 200 190 180 170 160 150 140 130 120 110 100 90 80 70 60 50 40 30 20 10 0

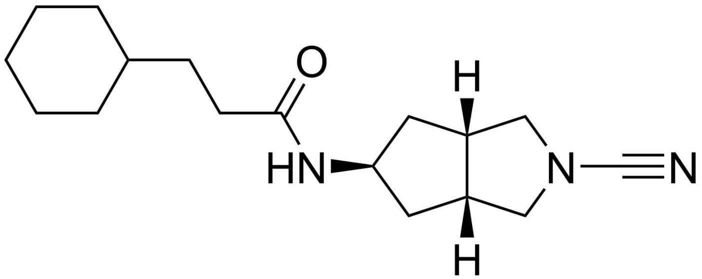

BB07CA48

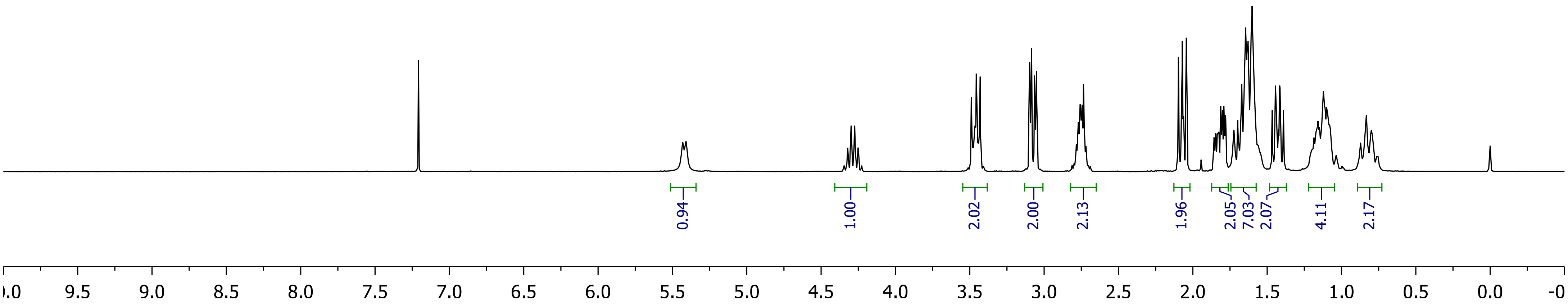
0.94
1.00
2.02
2.00
2.13
1.96
2.05
7.03
2.07
4.11
2.17
10.0
9.5
9.0
8.5
8.0
7.5
7.0
6.5
6.0
5.5
5.0
4.5
4.0
3.5
3.0
2.5
2.0
1.5
1.0
0.5
0.0
-0

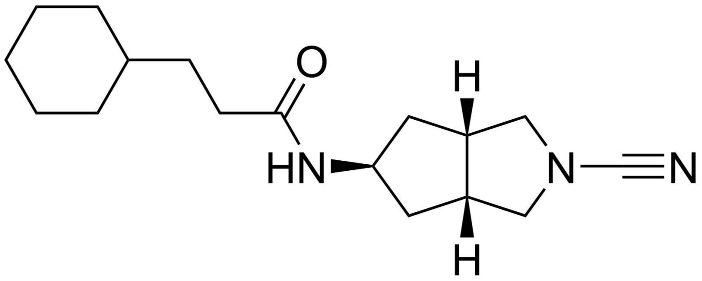

BB07CA48

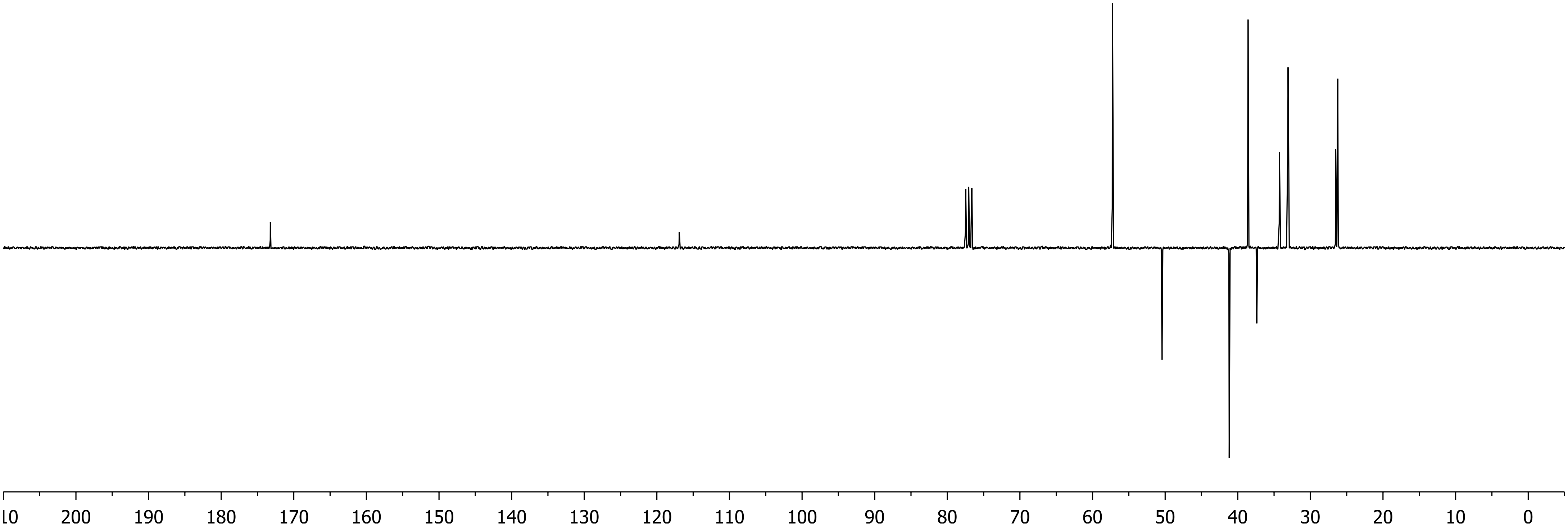
200
190
180
170
160
150
140
130
120
110
100
90
80
70
60
50
40
30
20
10
0

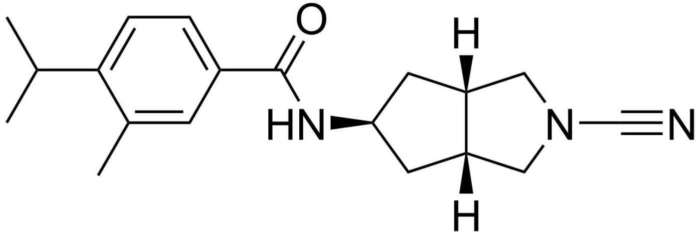
O
HN
H
H
N
N

BB07CA902

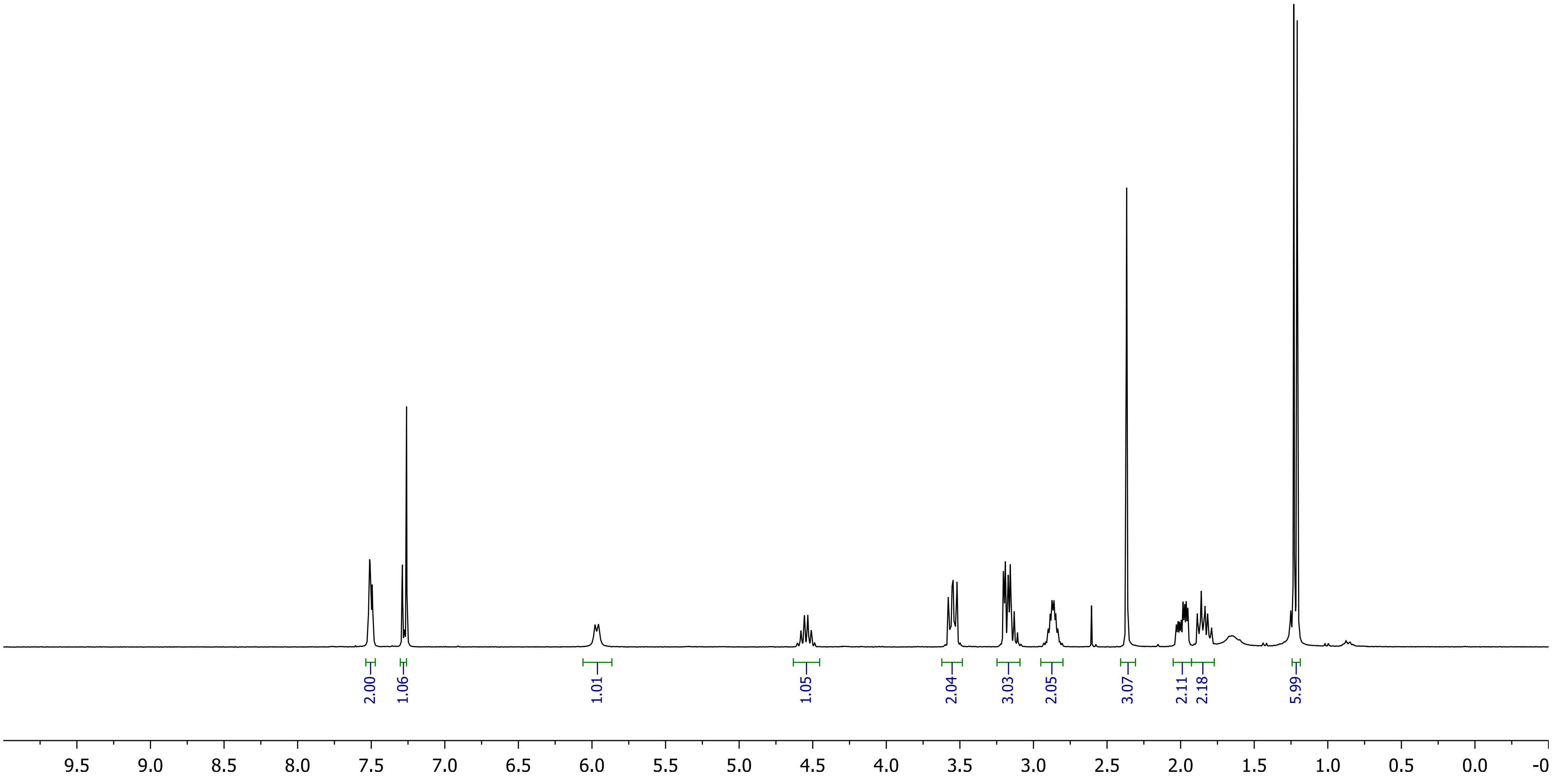
2.00
1.06
1.01
1.05
2.04
3.03
2.05
3.07
2.11
2.18
5.99
9.5
9.0
8.5
8.0
7.5
7.0
6.5
6.0
5.5
5.0
4.5
4.0
3.5
3.0
2.5
2.0
1.5
1.0
0.5
0.0
-0

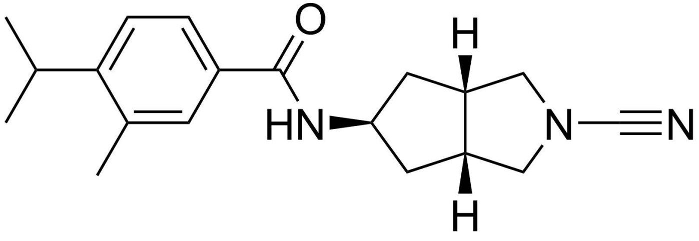

BB07CA902

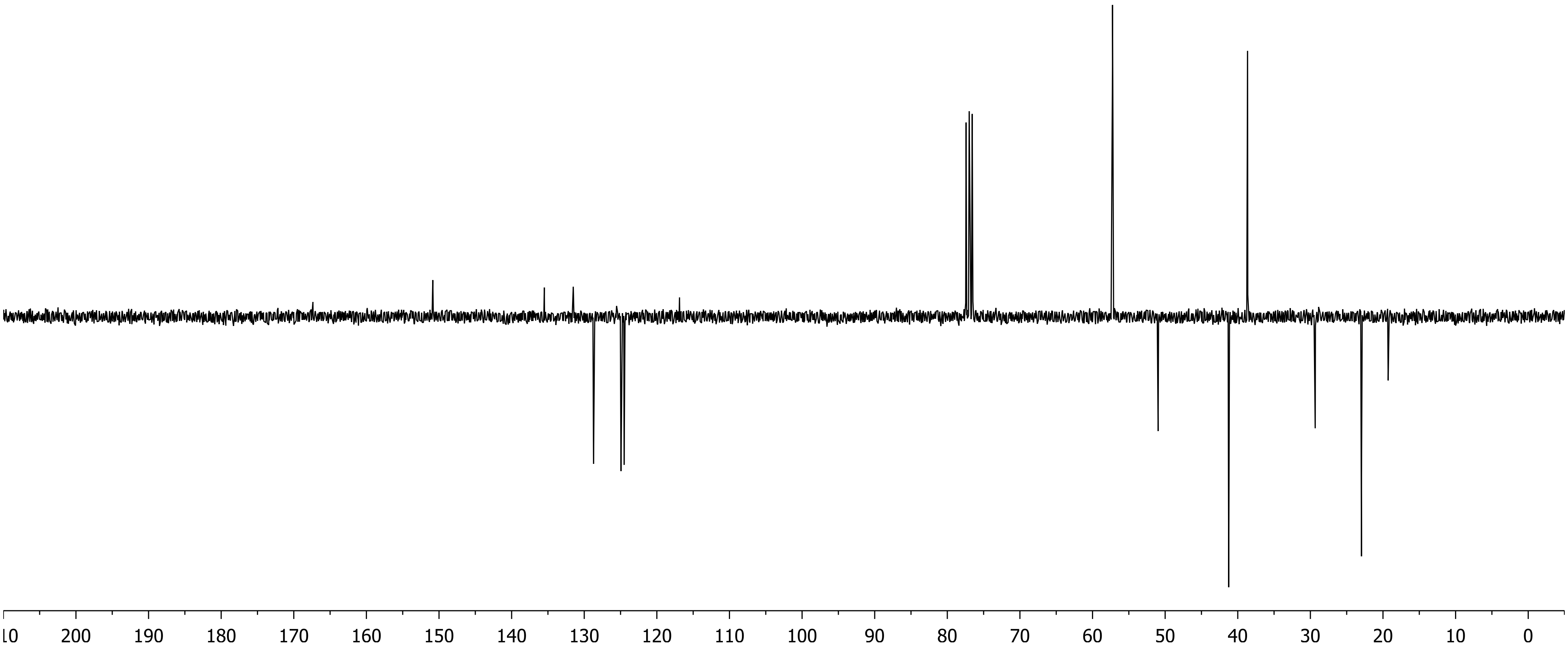
10
200
190
180
170
160
150
140
130
120
110
100
90
80
70
60
50
40
30
20
10
0

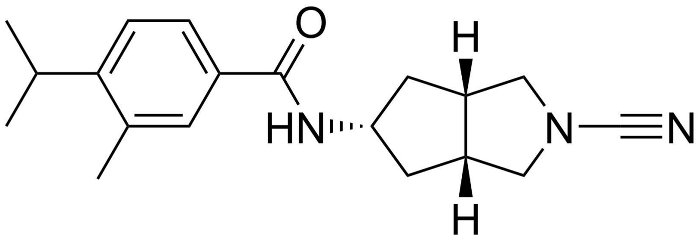

BB06CA902

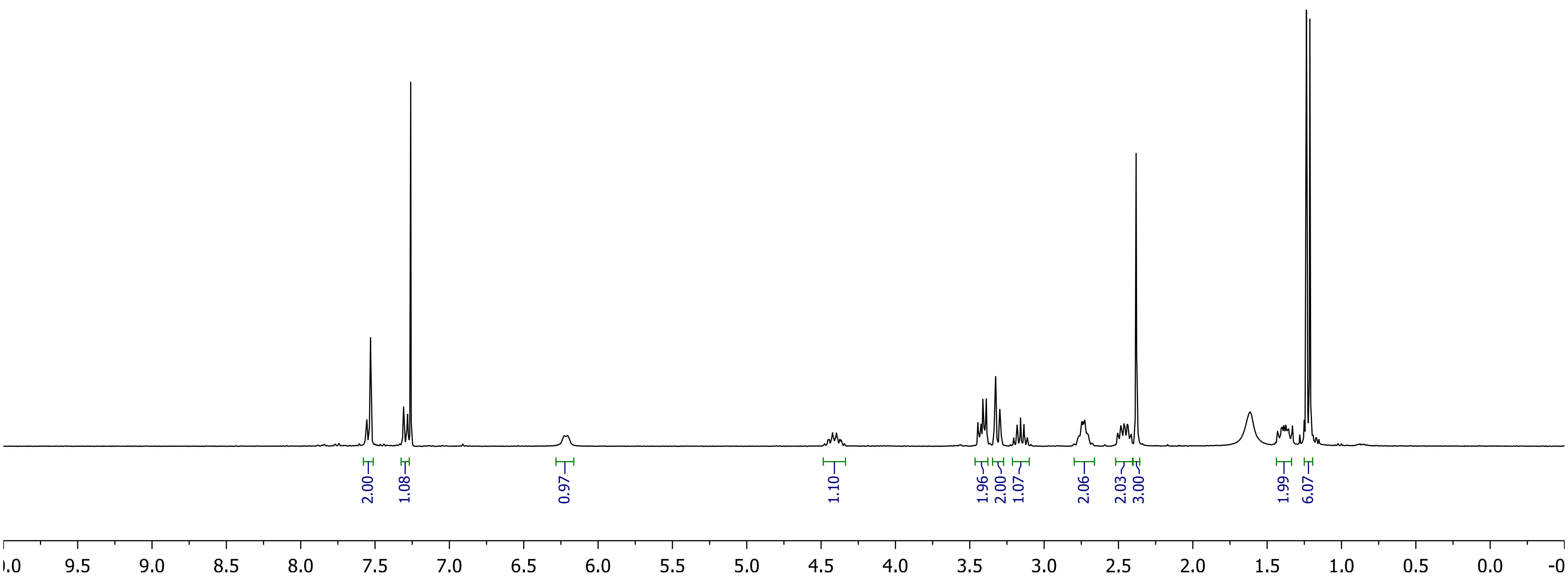

2.00
1.08
0.97
1.10
1.96
2.00
1.07
2.06
2.03
3.00
1.99
6.07
.0
9.5
9.0
8.5
8.0
7.5
7.0
6.5
6.0
5.5
5.0
4.5
4.0
3.5
3.0
2.5
2.0
1.5
1.0
0.5
0.0
-0

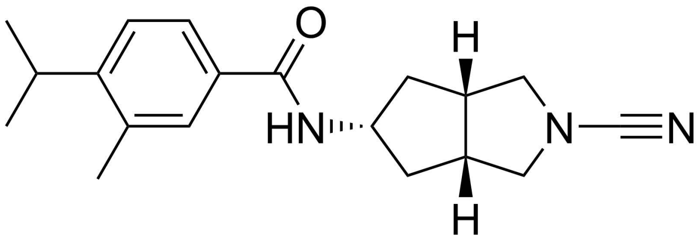
O
H
HN
N
N
H

BB06CA902

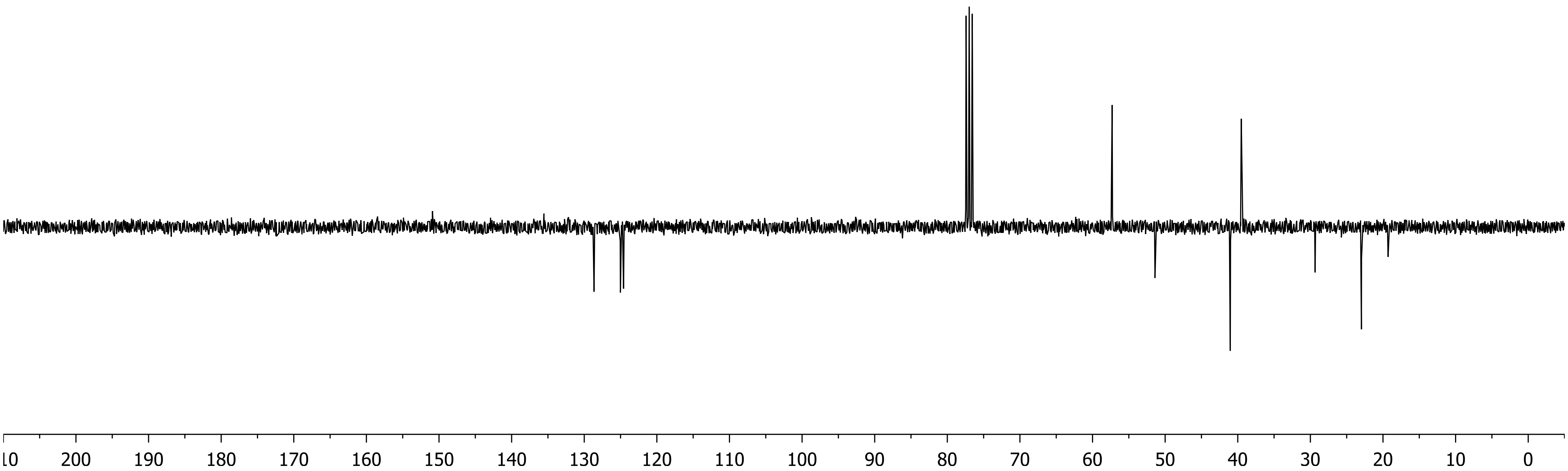
200
190
180
170
160
150
140
130
120
110
100
90
80
70
60
50
40
30
20
10
0

ISG15$_{CT}$-Rho-MP

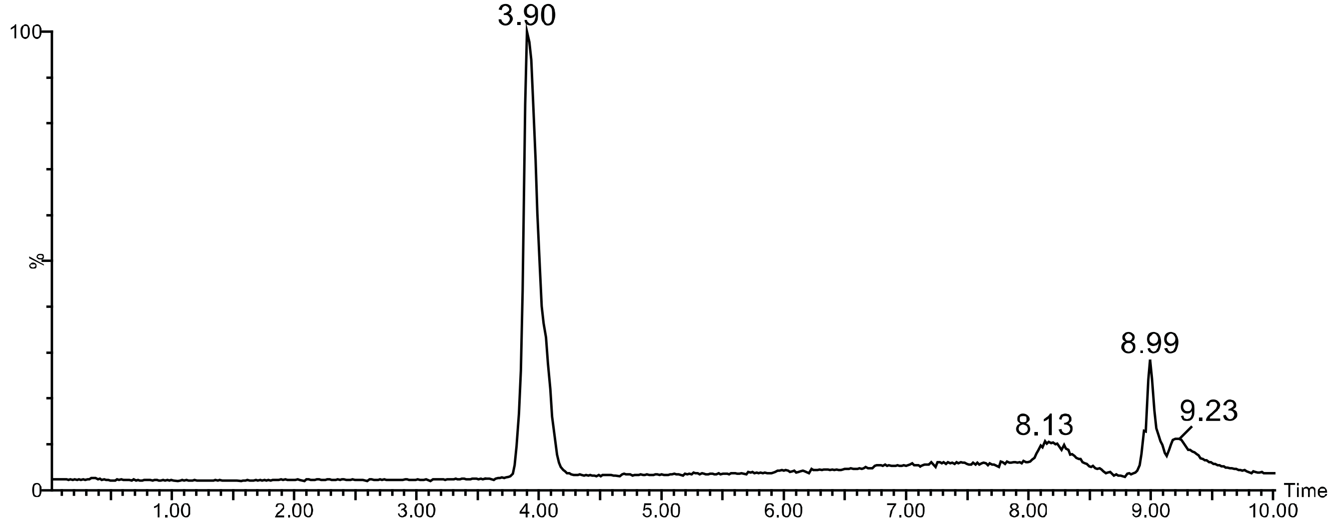

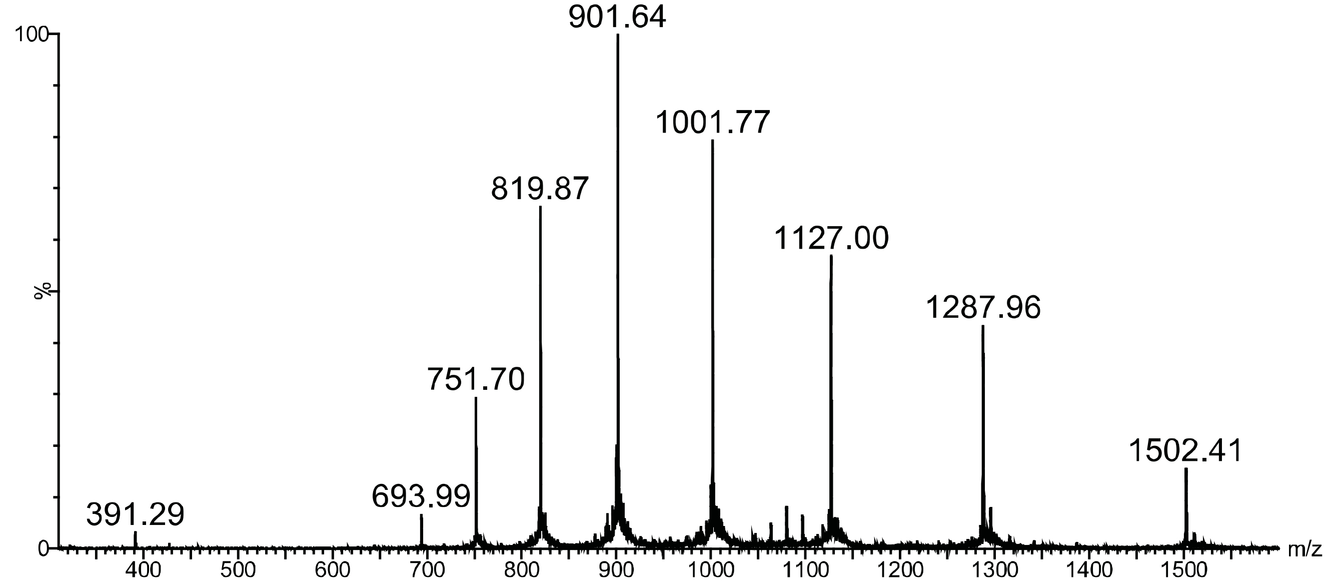

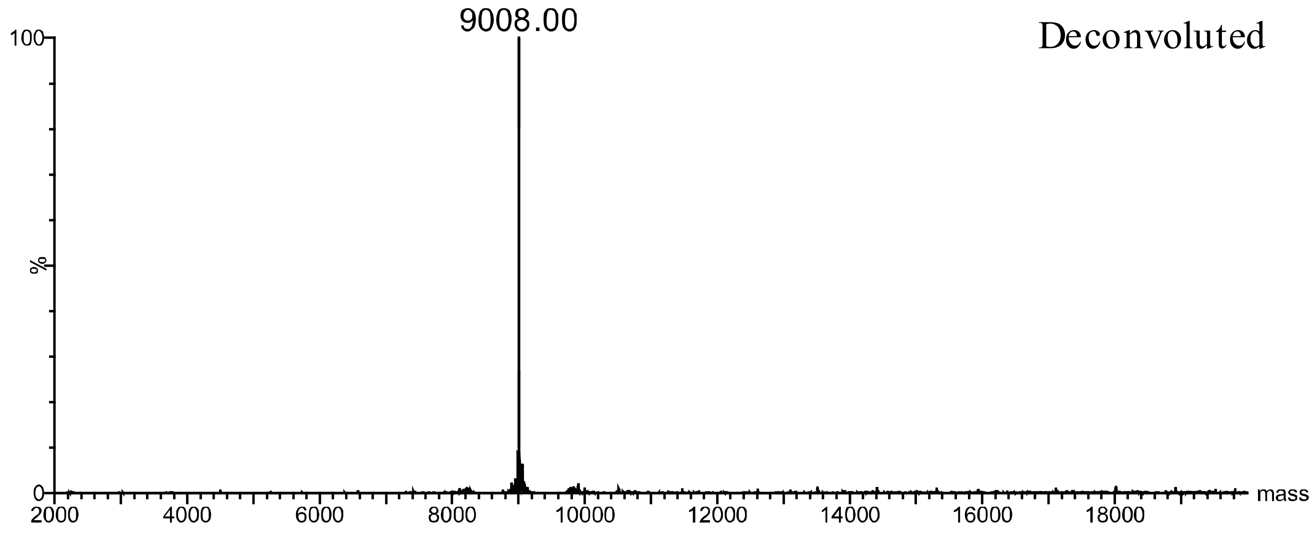

Compound 1

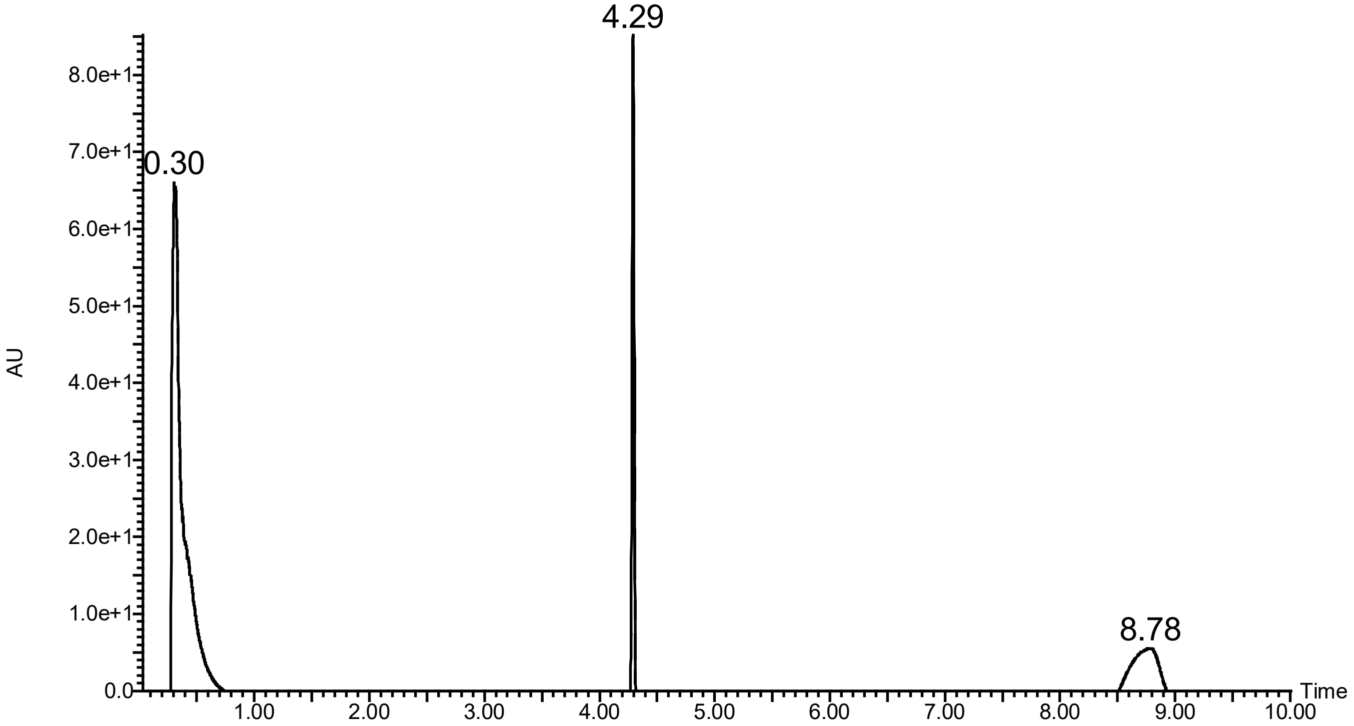
4.29
0.30
8.78
AU
8.0e+1
7.0e+1
6.0e+1
5.0e+1
4.0e+1
3.0e+1
2.0e+1
1.0e+1
0.0
Time
1.00
2.00
3.00
4.00
5.00
6.00
7.00
8.00
9.00
10.00

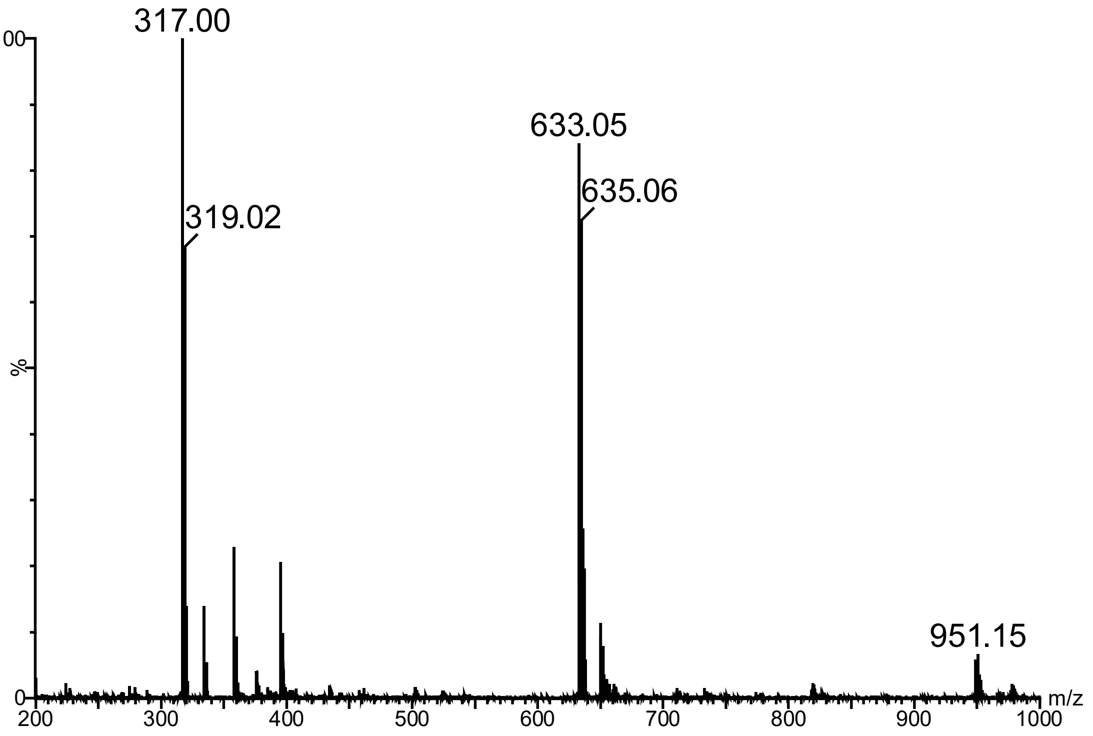
317.00
319.02
633.05
635.06
951.15
100
%
0
m/z
200
300
400
500
600
700
800
900
1000

Compound 2

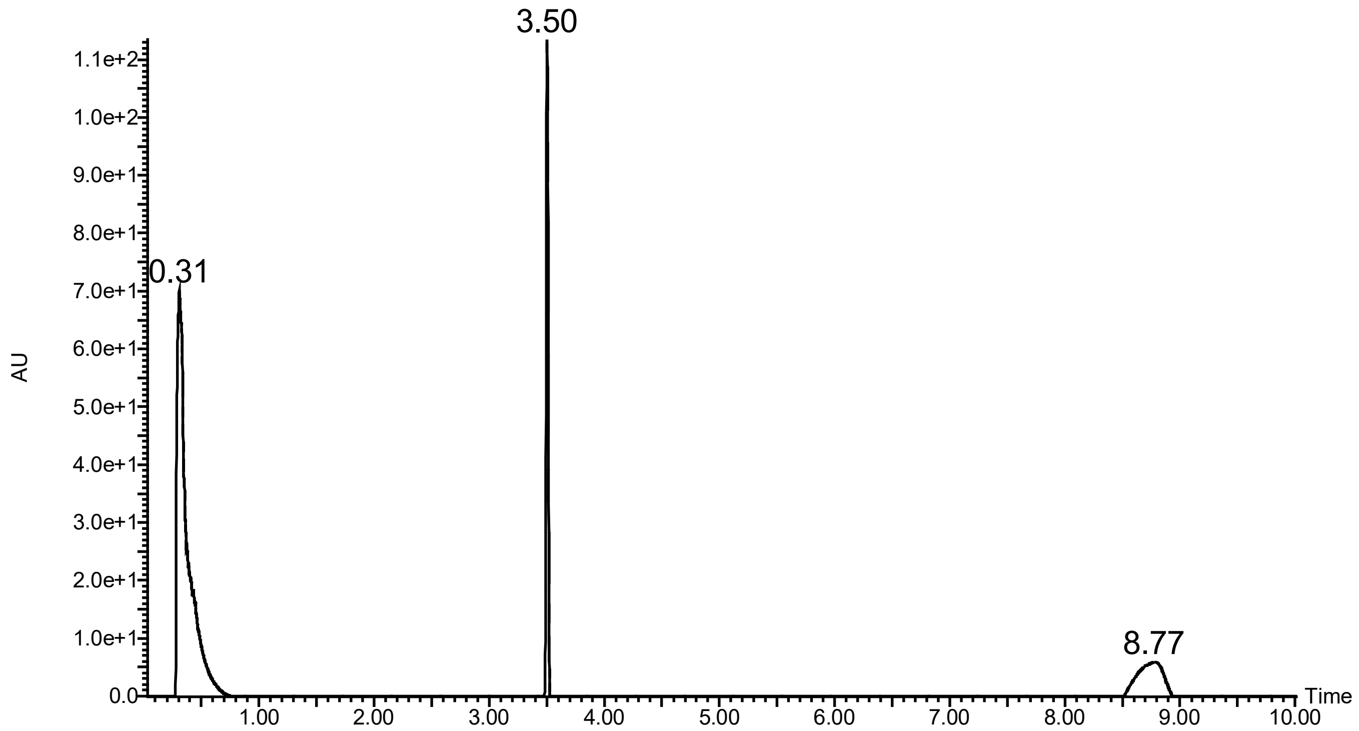
3.50
0.31
8.77
AU
1.1e+2
1.0e+2
9.0e+1
8.0e+1
7.0e+1
6.0e+1
5.0e+1
4.0e+1
3.0e+1
2.0e+1
1.0e+1
0.0
1.00
2.00
3.00
4.00
5.00
6.00
7.00
8.00
9.00
10.00
Time

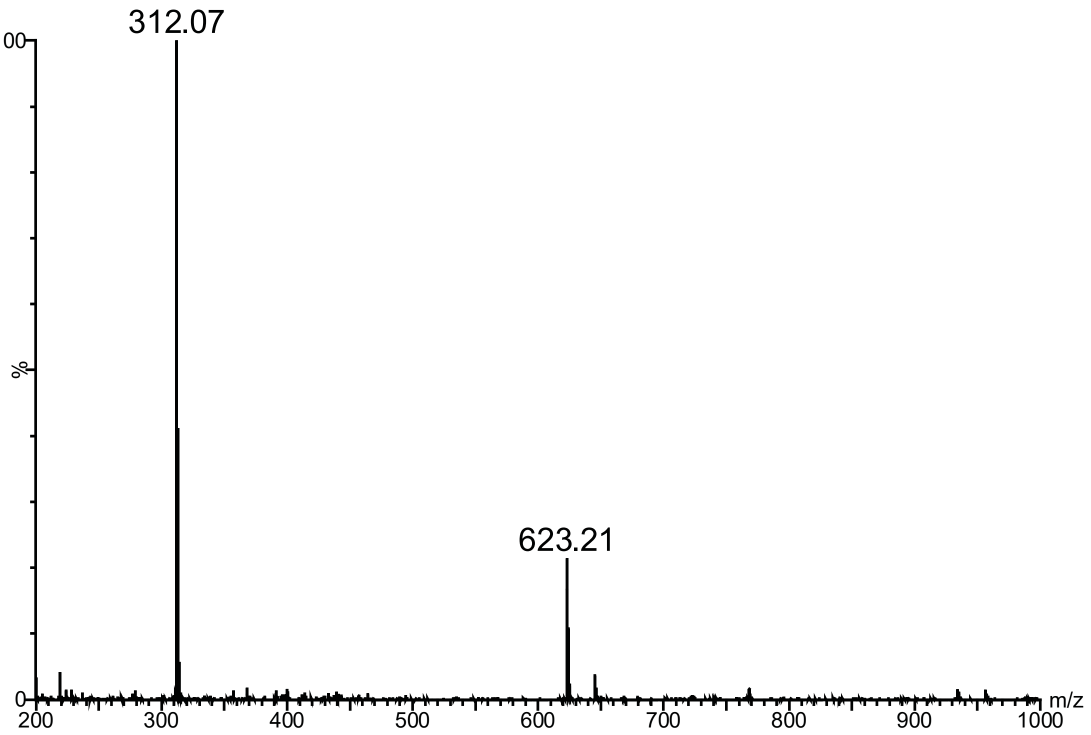
312.07
623.21
100
%
0
200
300
400
500
600
700
800
900
1000
m/z

Compound 3

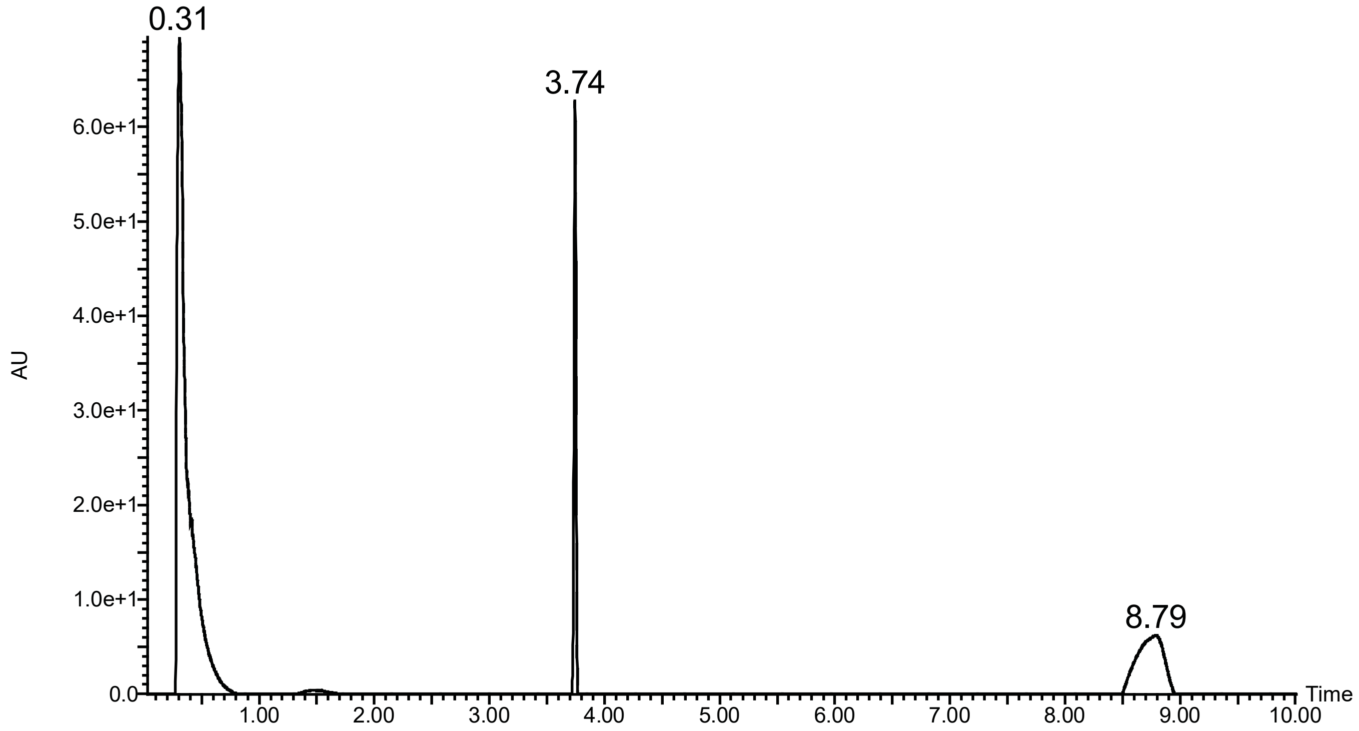
0.31
3.74
8.79
6.0e+1
5.0e+1
4.0e+1
3.0e+1
2.0e+1
1.0e+1
0.0
AU
1.00
2.00
3.00
4.00
5.00
6.00
7.00
8.00
9.00
10.00
Time

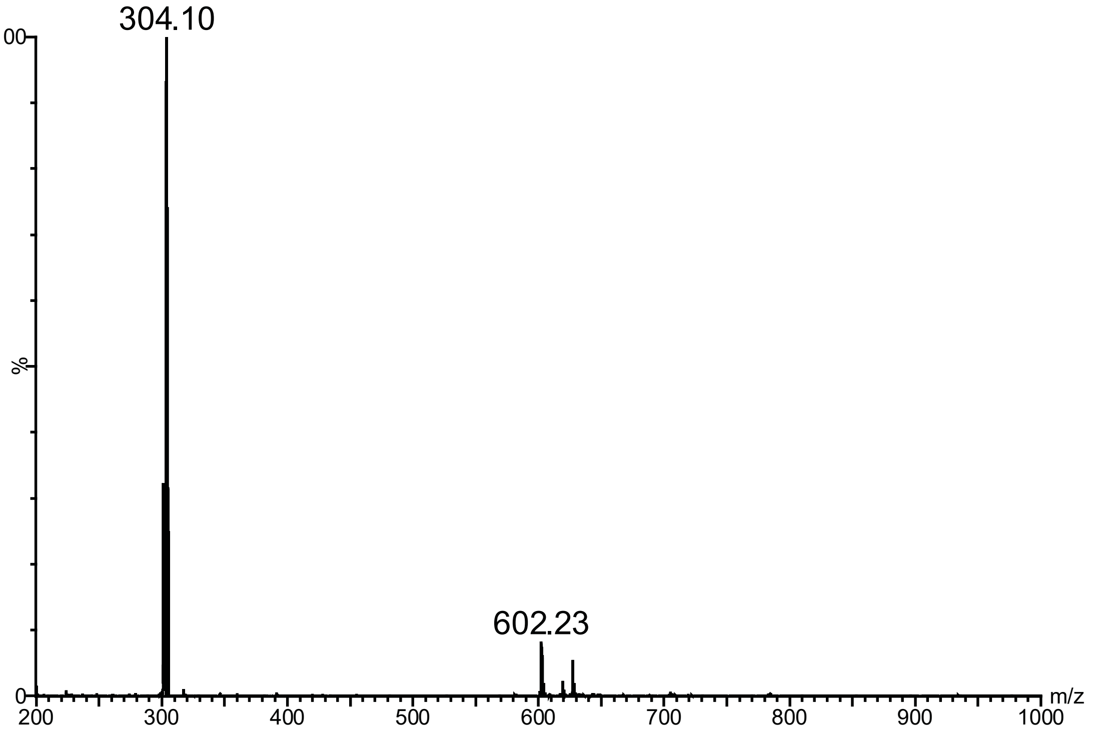
304.10
602.23
100
%
0
200
300
400
500
600
700
800
900
1000
m/z

Compound 4

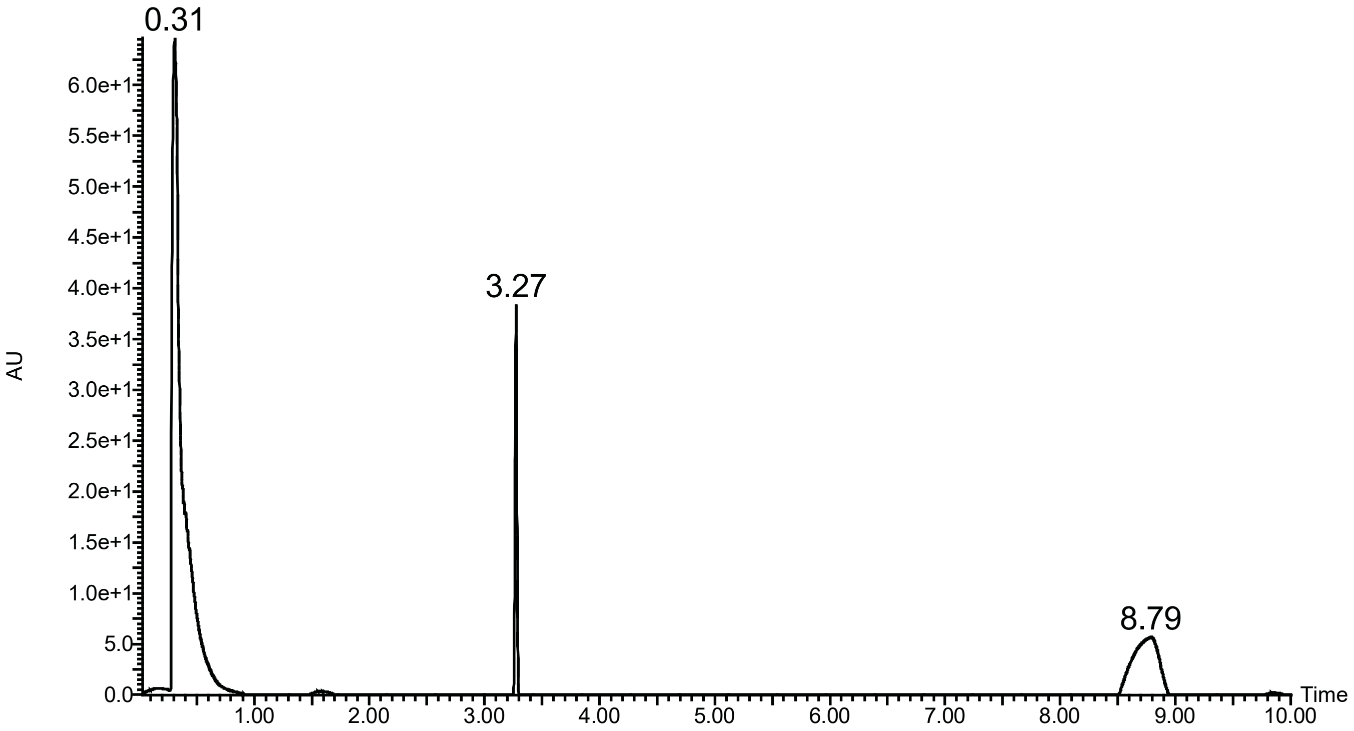

0.31
3.27
8.79
AU
6.0e+1
5.5e+1
5.0e+1
4.5e+1
4.0e+1
3.5e+1
3.0e+1
2.5e+1
2.0e+1
1.5e+1
1.0e+1
5.0
0.0
1.00
2.00
3.00
4.00
5.00
6.00
7.00
8.00
9.00
10.00
Time

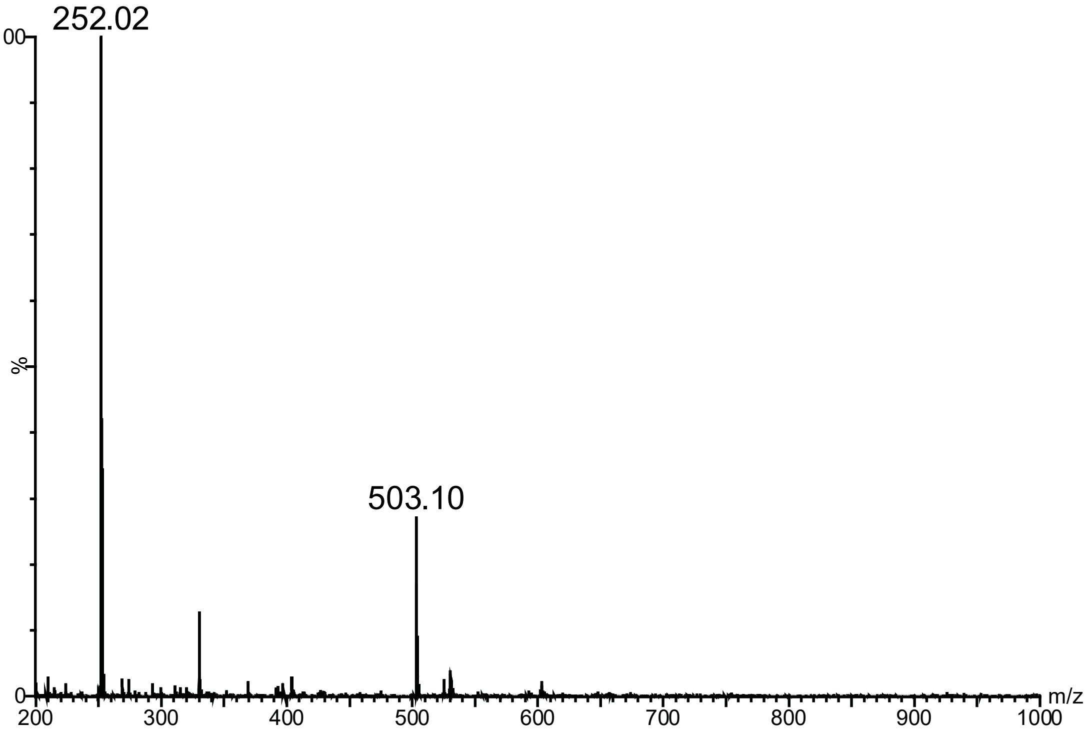

252.02
503.10
100
%
0
200
300
400
500
600
700
800
900
1000
m/z

Compound 5

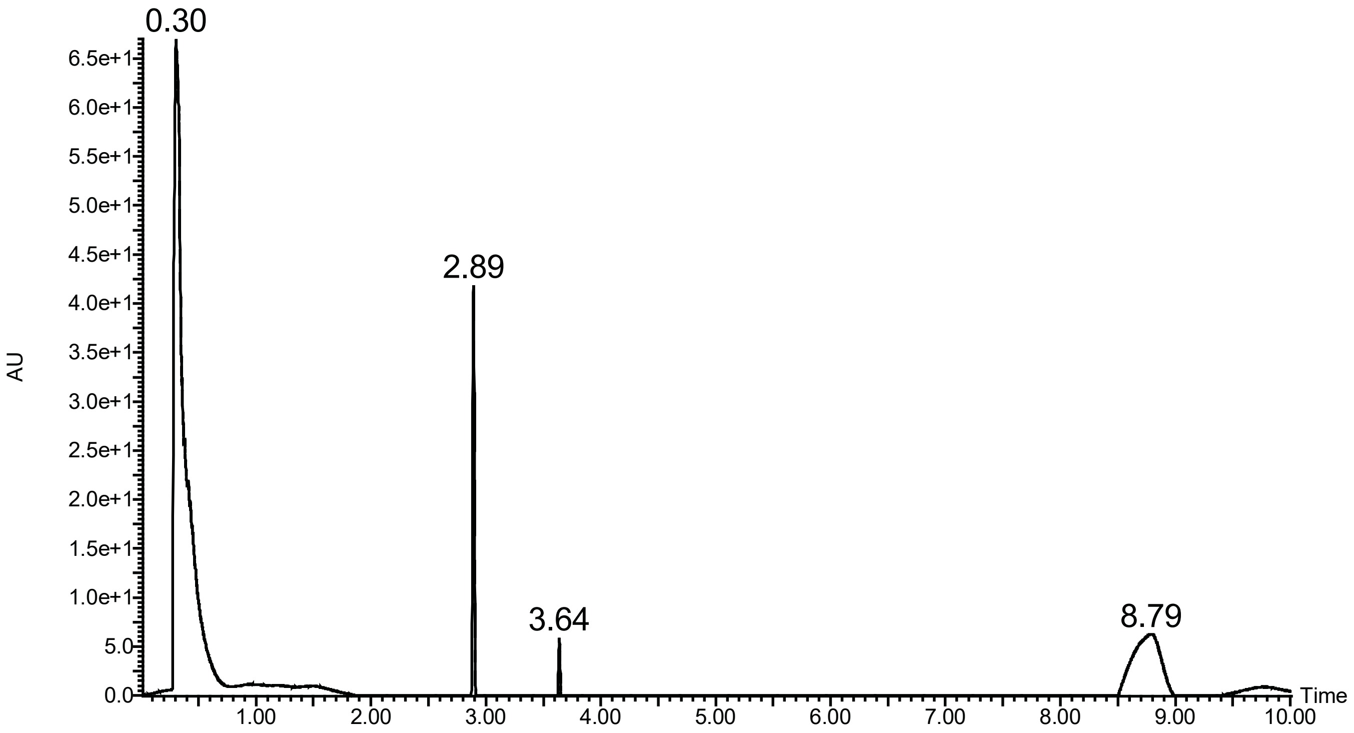

0.30
2.89
3.64
8.79
AU
6.5e+1
6.0e+1
5.5e+1
5.0e+1
4.5e+1
4.0e+1
3.5e+1
3.0e+1
2.5e+1
2.0e+1
1.5e+1
1.0e+1
5.0
0.0
1.00
2.00
3.00
4.00
5.00
6.00
7.00
8.00
9.00
10.00
Time

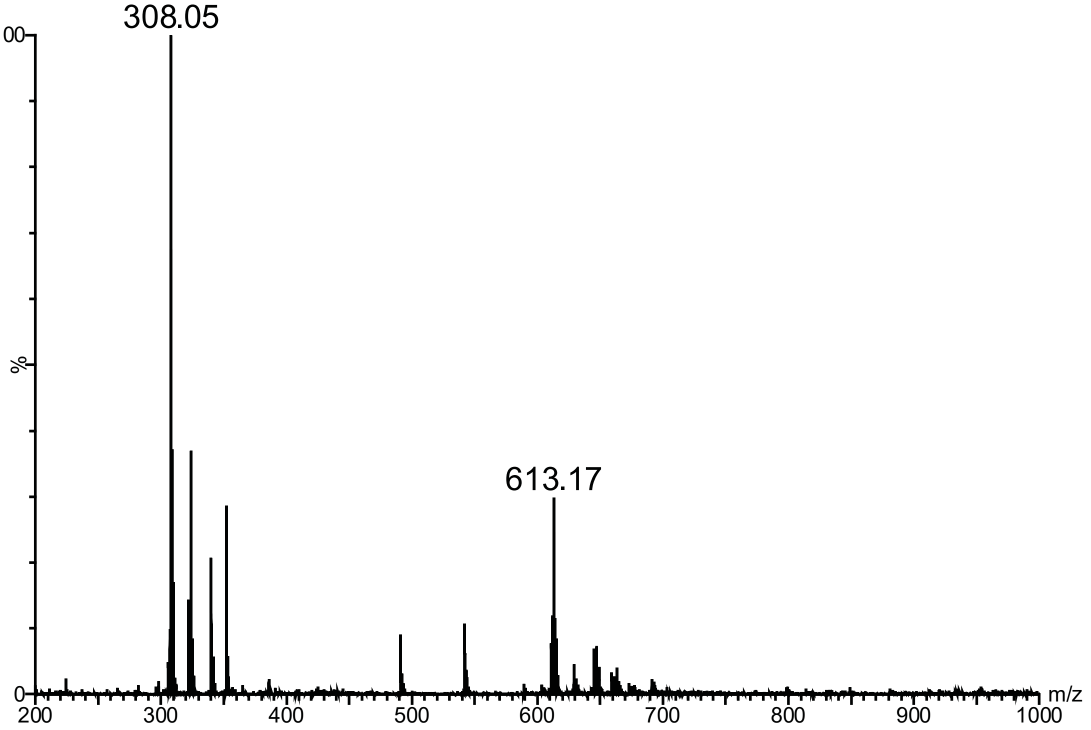

308.05
613.17
100
%
0
200
300
400
500
600
700
800
900
1000
m/z

BB07CA28

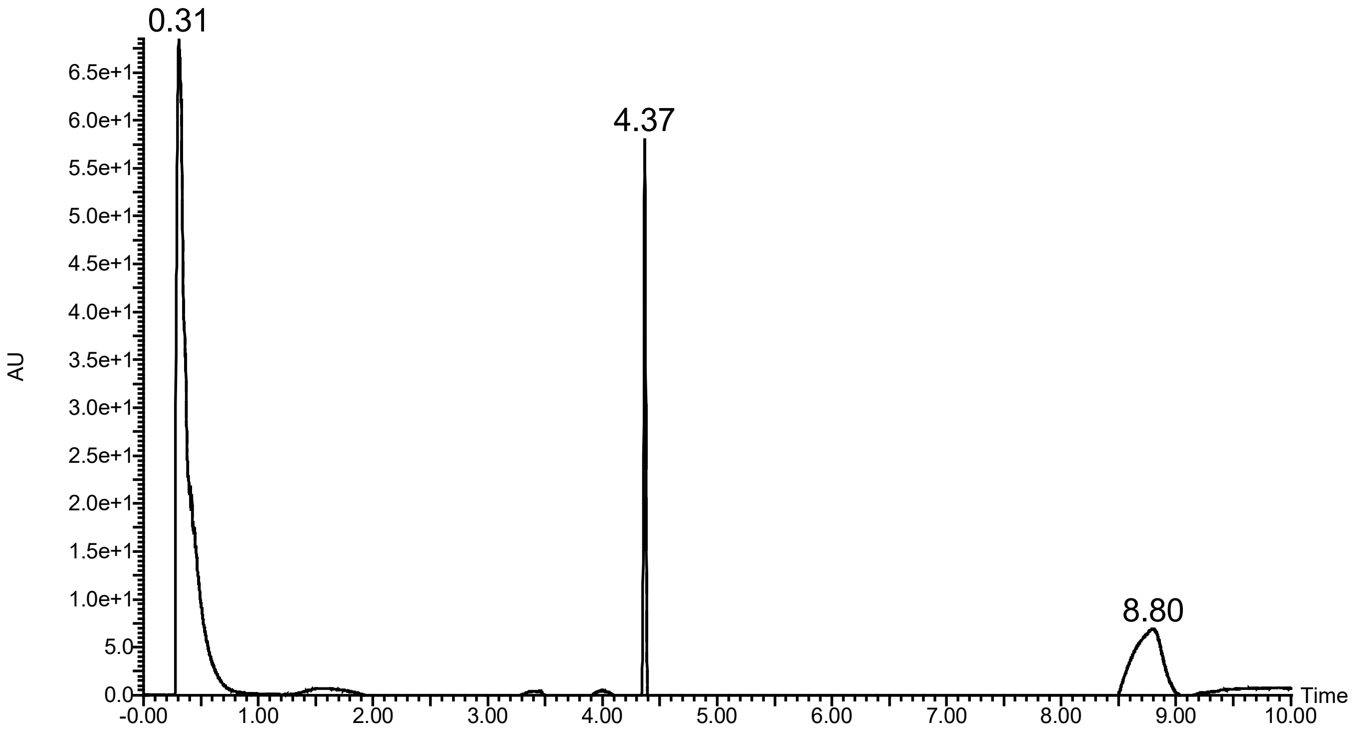
0.31
4.37
8.80
AU
6.5e+1
6.0e+1
5.5e+1
5.0e+1
4.5e+1
4.0e+1
3.5e+1
3.0e+1
2.5e+1
2.0e+1
1.5e+1
1.0e+1
5.0
0.0
Time
-0.00
1.00
2.00
3.00
4.00
5.00
6.00
7.00
8.00
9.00
10.00

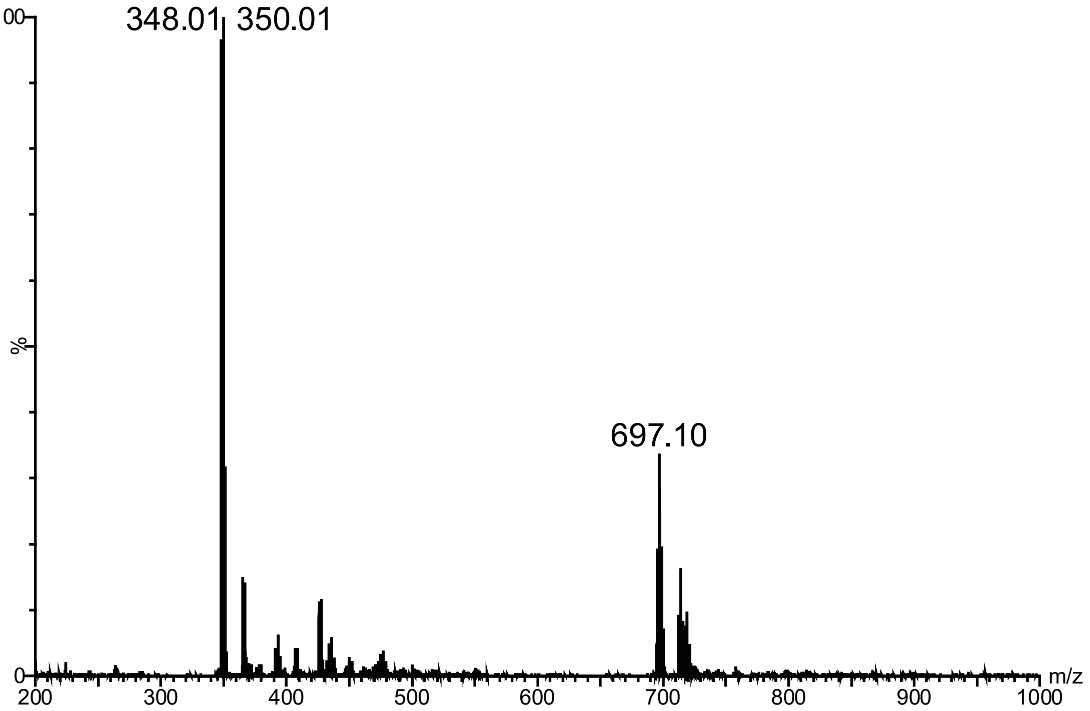
348.01
350.01
697.10
100
%
0
m/z
200
300
400
500
600
700
800
900
1000

BB07CA48

(not UV active)

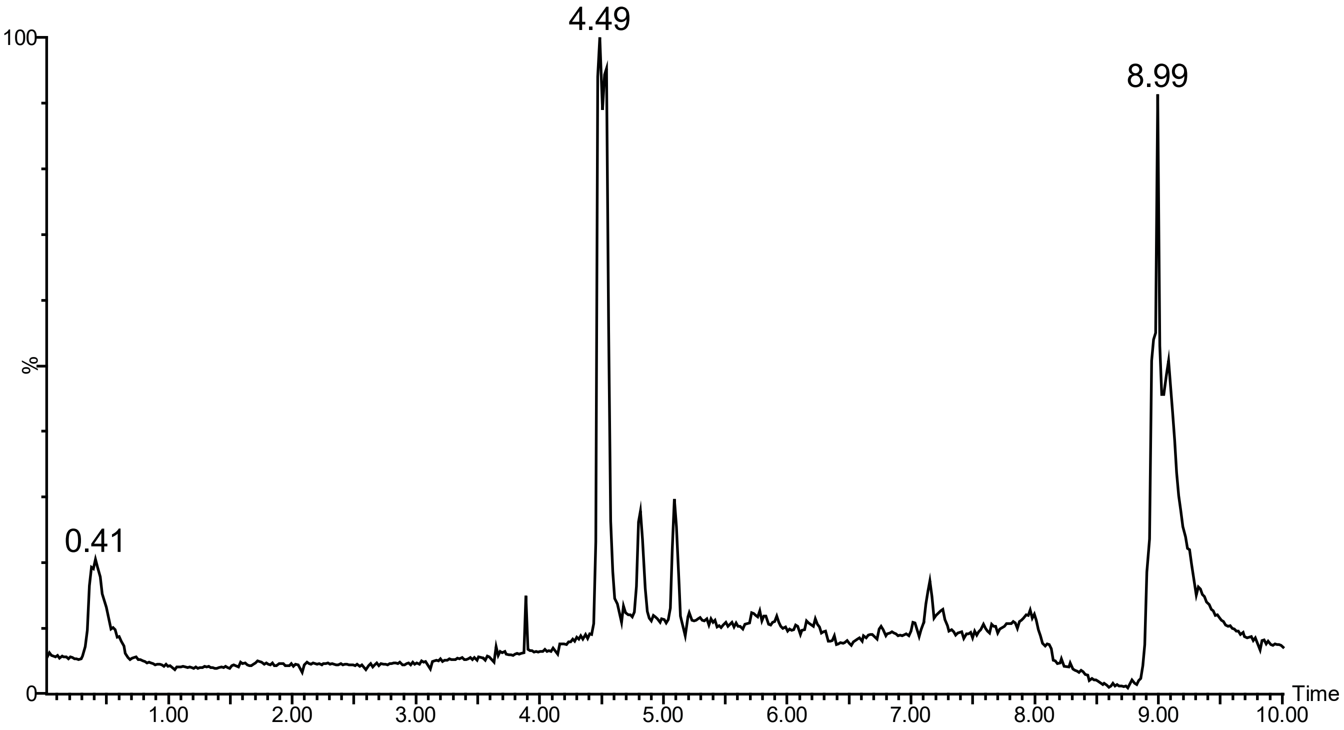

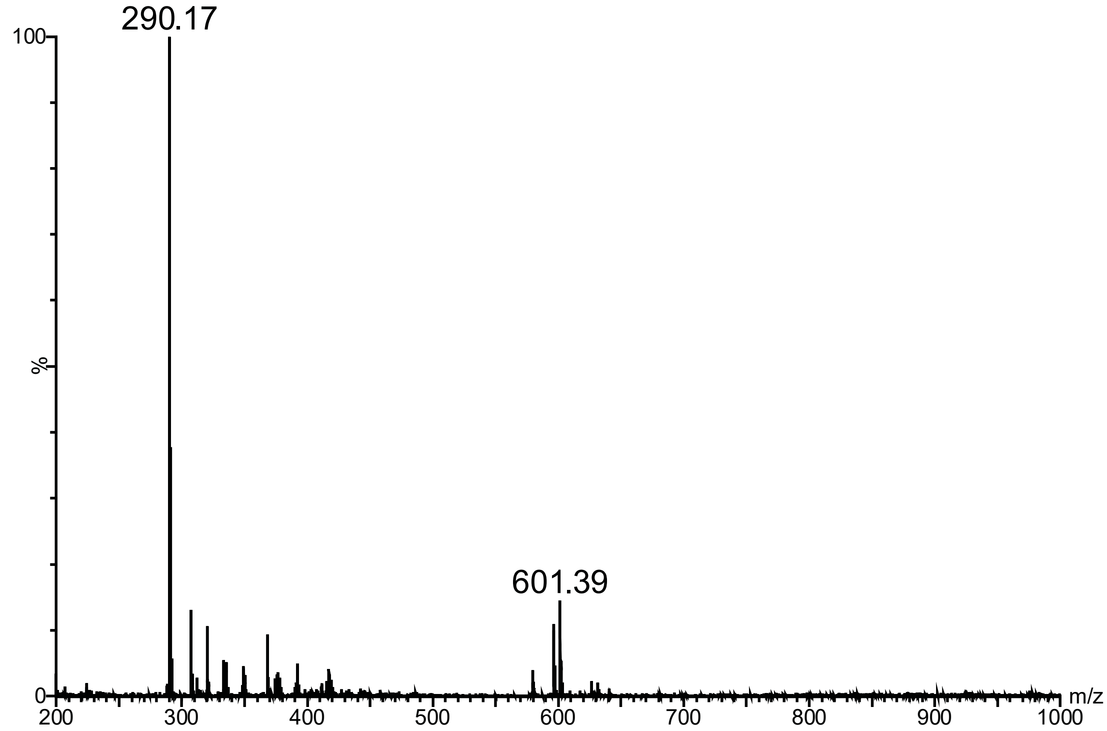

BB06CA902

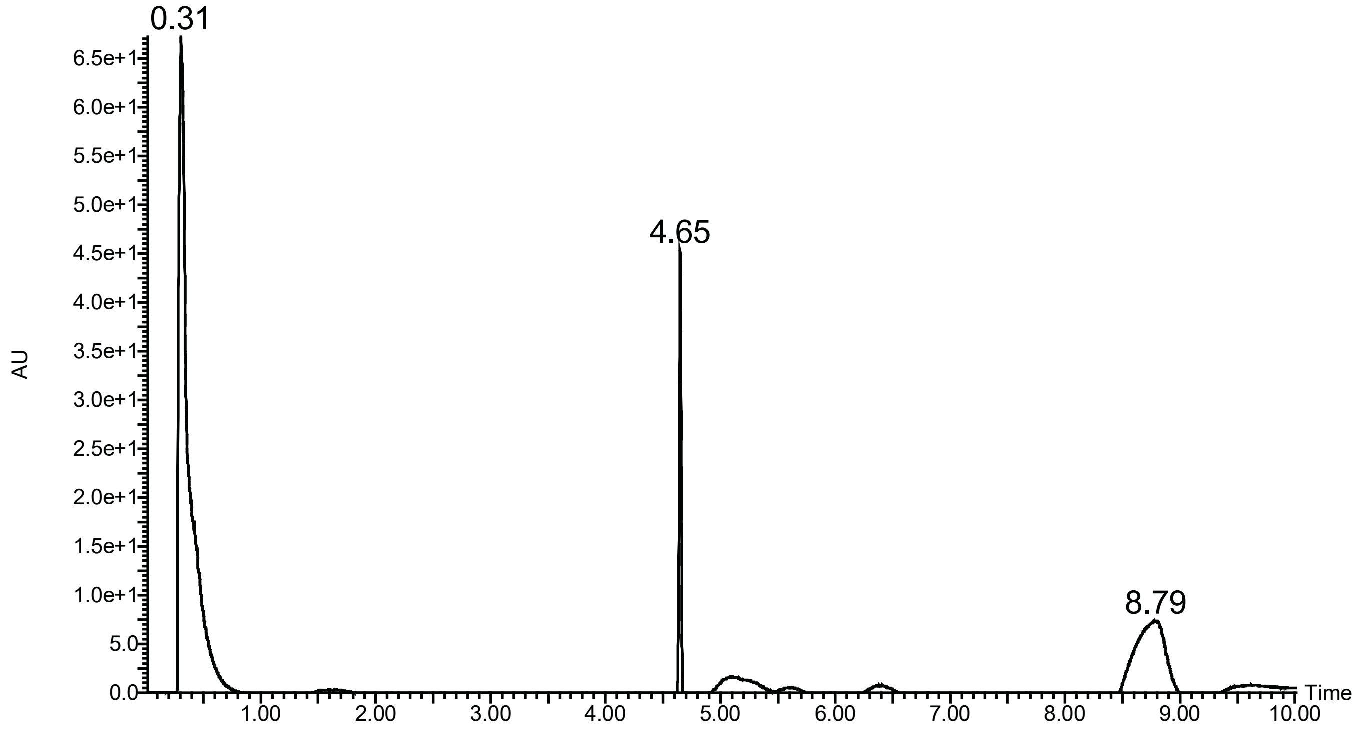

0.31
4.65
8.79
AU
6.5e+1
6.0e+1
5.5e+1
5.0e+1
4.5e+1
4.0e+1
3.5e+1
3.0e+1
2.5e+1
2.0e+1
1.5e+1
1.0e+1
5.0
0.0
1.00
2.00
3.00
4.00
5.00
6.00
7.00
8.00
9.00
10.00
Time

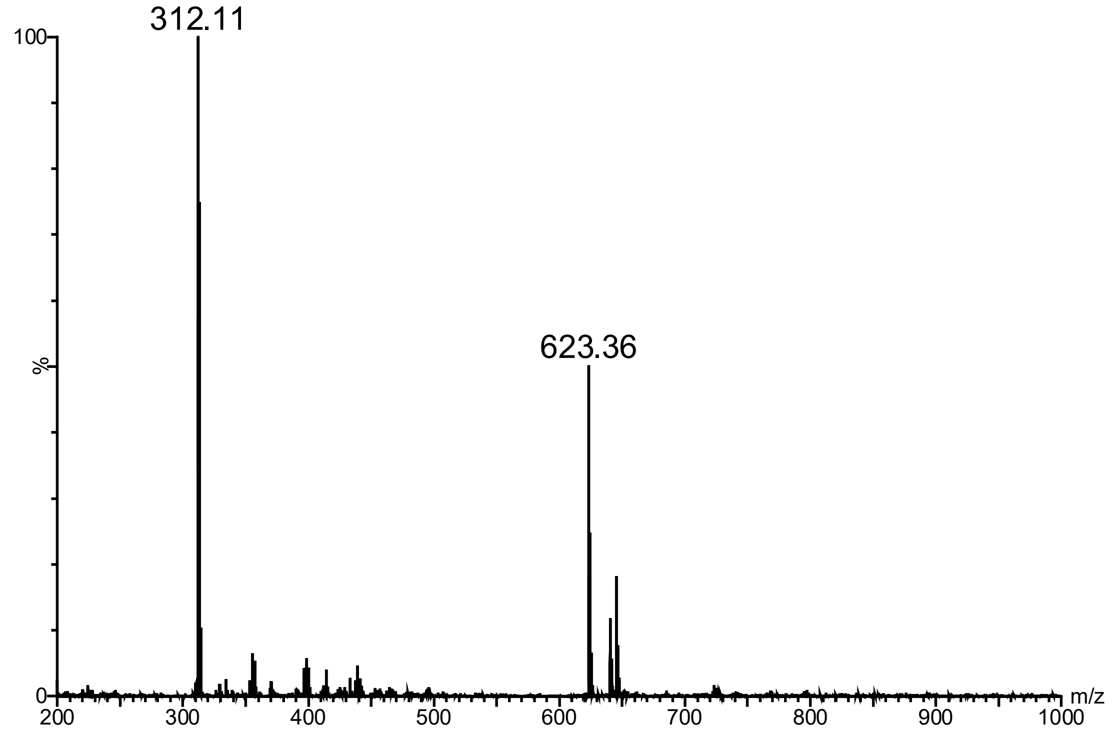

312.11
623.36
100
%
0
200
300
400
500
600
700
800
900
1000
m/z

BB07CA902

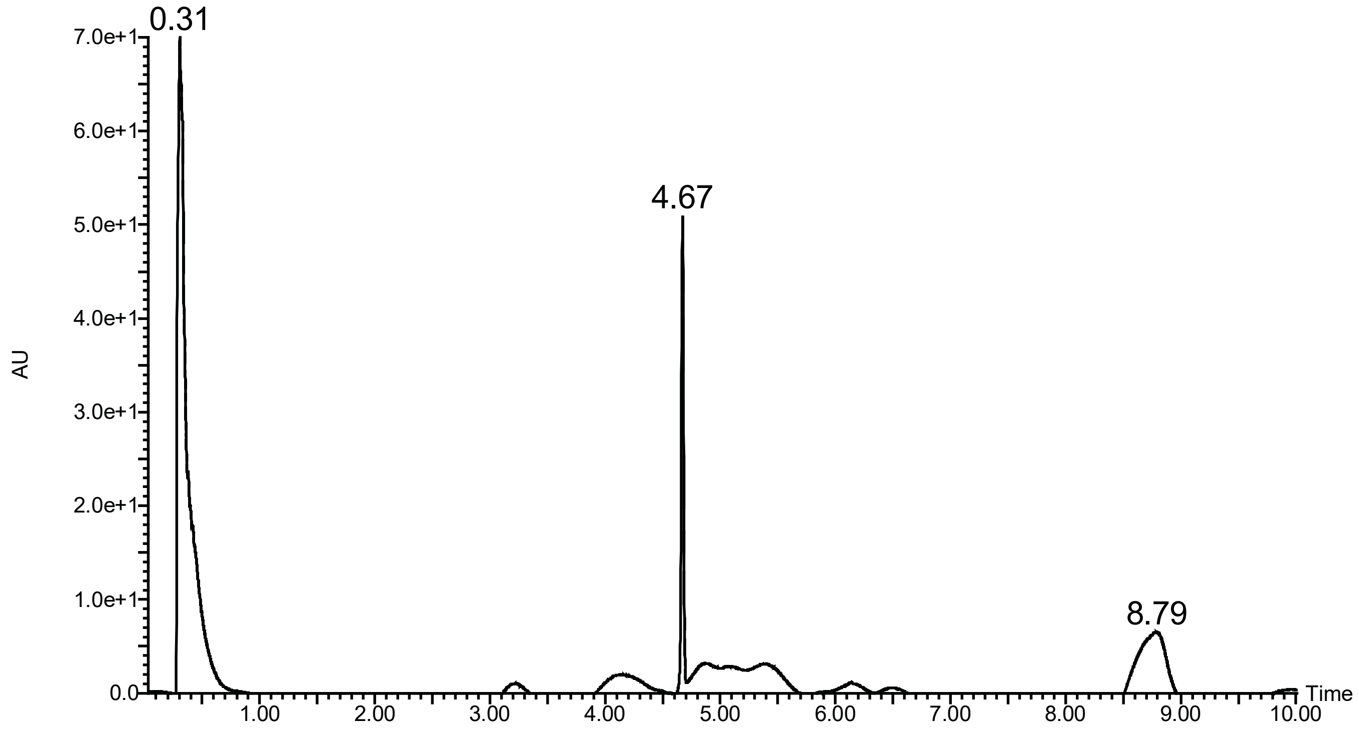
0.31
4.67
8.79
AU
7.0e+1
6.0e+1
5.0e+1
4.0e+1
3.0e+1
2.0e+1
1.0e+1
0.0
1.00
2.00
3.00
4.00
5.00
6.00
7.00
8.00
9.00
10.00
Time

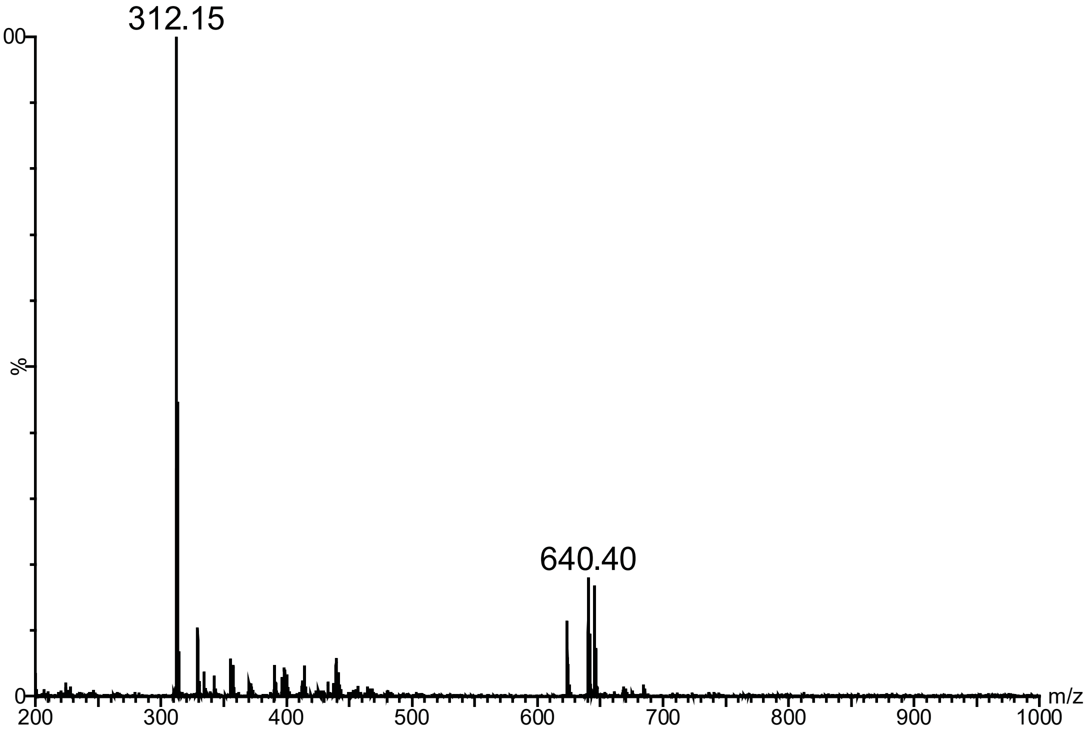
312.15
640.40
100
%
0
200
300
400
500
600
700
800
900
1000
m/z
